# Impact of a nurse anesthetist student–led training program on perioperative pain management in total knee replacement: A prospective before and after study

**DOI:** 10.1016/j.ijnsa.2026.100495

**Published:** 2026-01-27

**Authors:** Elie Guillen, Matthieu Jabaudon, Marc Garnier, Cathy Paulet, Cécile Vermeersch

**Affiliations:** a School of Nursing Anesthesia, CHU Clermont-Ferrand and Université Clermont Auvergne, Clermont-Ferrand, France; b Department of perioperative medicine, CHU Clermont-Ferrand, Clermont-Ferrand, France; c iGReD, CNRS, INSERM, Université Clermont Auvergne, Clermont-Ferrand, France

**Keywords:** Pain, Pain measurement, Surgery, Orthopedics, Training, Nurse anesthetists

## Abstract

**Background:**

Effective perioperative pain management is essential for patients undergoing total knee replacement. This study assessed professional practices related to perioperative pain evaluation and management at a French university hospital and evaluated the impact of a training program led by nurse anesthetist students.

**Methods:**

We conducted a prospective, single-center, observational before-after study (convenience sampling) from September 2022 to May 2023 at Clermont-Ferrand university hospital, France. Data were collected from paper medical records and a structured questionnaire administered to paramedical professionals. Pain assessment practices (traceability of numerical rating scale scores) and analgesic management were evaluated across four phases: two practice-assessment phases separated by a two-step training program.

**Results:**

Data from 51 to 85 patient medical records before the training (Phase 1) and 85 after the training (Phase 4) were analyzed. A total of 74 % of operating room staff and 58 % of orthopedic-ward staff completed the questionnaire used to develop the training program. Numerical rating scale traceability on orthopedic-ward admission improved from 29.6 % to 48.2 % (*P* = 0.03). Documentation of pain at post-anesthesia care unit discharge remained stable (78.4 % vs. 82.2 %; *P* = 0.61). Morphine administration and subsequent pain reassessment in the post-anesthesia care unit increased after training. Patient satisfaction with pain management remained high in both periods (90 % vs. 80 %).

**Conclusions:**

The nurse anesthetist student–led training program improved several aspects of perioperative pain evaluation and management in total knee replacement patients, particularly documentation and analgesic practices. These results highlight the value of involving nurse anesthetist students in quality-improvement initiatives and identify remaining gaps for future targeted interventions.

**What is already known**

• Pain assessment and management are essential after total knee replacement.
• Nursing documentation of pain remains inconsistent despite existing guidelines.
• Evidence on nurse-led education improving documented pain practices remains limited.

Alt-text: Unlabelled box dummy alt text

**What this paper adds**

• Brief nurse anesthetist student...led training improved pain assessment traceability.
• Improvements were greatest in the orthopedic ward; other areas were limited by record systems.
• Nurse anesthetist students can contribute to perioperative quality improvement.

Alt-text: Unlabelled box dummy alt text

## 1 Background

Pain is defined as *"an unpleasant sensory and emotional experience associated with, or resembling that associated with, actual or potential tissue damage"* (Raja et al., 2020)*.* In surgical settings, effective perioperative pain assessment and management are key determinants of patient recovery, satisfaction, and overall quality of care, and they represent a recognized public health priority (Aubrun et al., 2019). In France, pain management is also a legal requirement for healthcare professionals. The French Public Health Code (Articles L1110–5, Articles R4311–2, 5 and 12) mandates the prevention, evaluation, and treatment of pain as part of standard nursing practice. Nurse anesthetists (IADEs) play a central role in perioperative analgesia by performing advanced anesthesia and analgesic techniques under the medical supervision of an anesthesiologist, as defined in their national scope of practice.

Orthopedic surgery is associated with significant perioperative pain, particularly total knee replacement, where moderate-to-severe postoperative pain remains common despite multimodal analgesia strategies (Aubrun et al., 2019; Liu et al., 2012). Regional anesthesia techniques – such as femoral nerve block, adductor canal block, or periarticular infiltration – are increasingly used in total knee replacement for opioid-sparing pain control, though their effectiveness may vary depending on technique, timing, and postoperative monitoring.

The aims of this study were to evaluate professional practices regarding perioperative pain assessment traceability and management in total knee replacement at a French university hospital to determine whether a nurse anesthetists-led training program could improve these documentation practices and associated care processes.

## 2 Methods

### 2.1 Study design and hypothesis

We conducted a prospective, observational before-after, single-center study designed and conducted by faculty and students from the Nursing Anesthesia school at Clermont-Ferrand university hospital, a French university hospital. Approved by the local Ethics Committee (number 2022-CF-073; IRB00013412, “*CHU de Clermont Ferrand* IRB #1″), the study waived informed consent requirements. The primary hypothesis was that a targeted training program led by nurse anesthetist students would improve traceability of pain assessment and related perioperative pain-management practices in total knee replacement patients.

### 2.2 Sampling method

A convenience sampling strategy was use for both patient charts and participating healthcare professionals. The sample size was based of the recruitment feasibility within the predefined educational timeframe rather than on a formal power calculation.

### 2.3 Inclusion criteria for patients (Phases 1 and 4)

• Adults ≥18 years
• Undergoing primary unilateral total knee replacement
• Available medical record covering the full perioperative period at Clermont-Ferrand university hospital

**Exclusion criteria**

• revision knee arthroplasty
• trauma-related or tumor-related knee surgery
• incomplete or unavailable perioperative documentation

### 2.4 Healthcare professional participants (Phase 2 and 3)

Nurse anesthetists, post-anesthesia care unit nurses, and orthopedic-ward nurses involved in perioperative care of patients were eligible. Participation was voluntary.

**Fig. 1:**
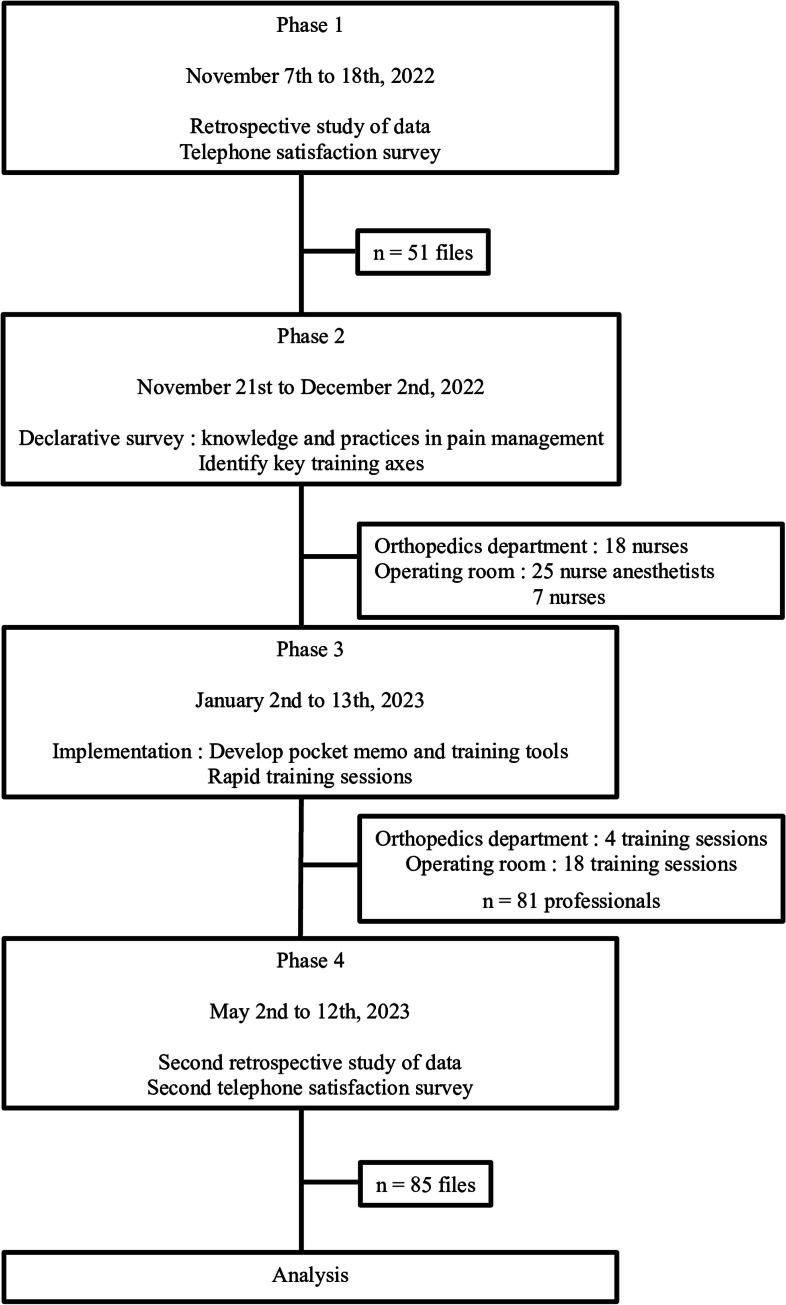
Study flowchart.

This study was conducted in four consecutive phases (Fig. 1).

Phase 1 was conducted from November 7, 2022 to November 18, 2022. It consisted of a retrospective study of data from 51 patients’ health medical records and a telephone satisfaction survey with each patient to assess their satisfaction regarding pain management.

Phase 2 (from November 21, 2022 to December 2, 2022) consisted of a declarative survey distributed to nurse anesthetists, post-anesthesia care unit nurses, and nurses working in the orthopedic surgery ward, to evaluate their knowledge regarding pain assessment and traceability, their analgesic administration practices, and their level of satisfaction with acute pain management with patients undergoing total knee replacement surgery. The results from this survey were used to identify the key training axes to be developed in the next phase.

In Phase 3 (from January 2, 2023 to January 13, 2022), a pocket memo and two rapid-training tools were developed to deliver key messages on pain management during total knee replacement surgery. One of the two training tools was designed for operating room nurses, nurse anesthetists, and post-anesthesia care unit nurses, while the other was developed for orthopedic ward nurses. In addition to the pocket memo and two rapid-training tools which were distributed to nurses, rapid training sessions (approximately 10 min) were organized for all nurses working during the day or night in the operating room, post-anesthesia care unit, and surgery ward.

From May 2, 2023 to May 12, 2022, Phase 4 consisted of a new retrospective study on clinical data from health medical records from 85 patients, along with telephone satisfaction surveys, to assess whether the training program developed in Phase 3 had any effect on pain assessment, traceability, and management or on patient satisfaction.

### 2.5 Training program

Phases 1 and 2 highlighted several areas of potential improvement in pain management and traceability assessment. In the orthopedic surgery ward, the traceability of pain assessment was the main element on which it seemed mandatory to improve practices, especially at ward admission (before surgery) and after administration of an analgesic treatment in the postoperative period (to evaluate efficacy).

In the operating room, we identified two main improvement areas: to make traceability of pain assessment more systematic, even in the absence of pain, and to better fill the patient’s health record with more details on regional anesthesia when used in association with systemic analgesia, such as the regional anesthesia techniques being used and evaluation of sensory and motor blocks over time.

The lack of systematic administration of (at least some) analgesic treatments in the postoperative period was another area of potential improvement. Therefore, an objective was to raise awareness among post-anesthesia care unit and surgical ward nurses on the mechanisms of pain and the action of each analgesic treatment.

The pocket-memo and rapid-training tools developed based on these areas of potential improvement are available in the **Appendix**.

### 2.6 Data collection tools

Clinical data were obtained from electronic medical records using REDCap electronic data capture tools (Harris et al., 2009). Variables included : past medical history, baseline demographics, routine clinico-biological variables, traceability of pain assessment, pain management and perioperative analgesia. They were obtained and anonymously collected from their health medical records before, during, and after surgery. Investigators completed a structured questionnaire created for the study including multiple-choice and Likert-scale items. Telephone questionnaires used standardized items to assess patient satisfaction regarding perioperative pain management.

### 2.7 Study outcomes

The primary study outcome measure was traceability of pain assessment, using the numeric rating scale for pain (which ranges from 0 to 10, with higher values indicating more severe pain), upon admission to the hospital (namely, to the orthopedic surgery ward). This outcome was chosen as pain assessment at baseline is a recognized marker and an objective of quality of care at our institution and in many others.

Secondary outcome measures included: traceability of pain assessment (using the numerical rating scale) upon arrival to the operating room (before surgery), in the post-anesthesia care unit, and in the surgical ward after surgery; modalities of administration of analgesics in the perioperative period; traceability of pain assessment and reassessment after administration of analgesics; and patient satisfaction with regards to pain management.

### 2.8 Statistical analysis

Statistical analysis was performed using *jamovi* (version 2.5, the jamovi project, Sidney, Australia) and Stata (version 15, StataCorp, College Station, USA) softwares. Statistical significance was established by a P value of <0.05 using two-sided hypothesis tests. We did not correct for multiple comparisons and results should be interpreted as exploratory. No missing data were imputed.

The number of patients was not formally estimated based on power calculation, but rather estimated based on the recruitment capacity within the allotted time, as this work was part of an educational program developed in 2022–2023 by the Nursing Anesthesia school at Clermont-Ferrand university hospital.

Categorical data were expressed as frequencies and associated percentages, and continuous data as means ± standard deviations (SD) or medians [interquartile ranges (IQR)], depending on their distribution.

Outcome measures were compared between Phase 1 and Phase 4: Student’s and Mann-Whitney tests were considered for quantitative parameters according to t-test assumptions (normality assumption and homoscedasticity using Shapiro-Wilk and Fisher-Snedecor tests, respectively), and categorical data were compared using chi-square or Fisher exact tests. All IC95 % were established using the Wilson IC method.

## 3 Development and implementation of the training program

### 3.1 Declarative survey of nurses

During Phase 2, 32 operating room nurses and nurse anesthetists (74 % of total staff) and 18 nurses working in the orthopedic ward (58 % of total staff) were surveyed (overall response rate of 68 %). Among operating room nurses, 78 % were nurse anesthetists and 22 % were general nurses. Overall, previous professional experience was >10 years in 53 % of respondents.

In the orthopedic surgery war, nurses reported that they evaluated the numerical rating scale for pain in 83 % of cases upon ward admission and that they traced it in 100 % of cases. The numerical rating scale threshold to administer an analgesic was reported as 3/10 by 54 % of nurses. For 70 % of them, analgesics were not administered to patients if they were not painful in the preoperative period.

In the operating room, 20 nurses (62.5 %) declared that they assessed preoperative pain, although traceability was only found in 17.6 % of medical records.

Only 2 (6 %) nurses knew about all the five regional anesthesia techniques which can be potentially used for total knee replacement surgery (femoral block, sciatic nerve block, adductor canal block, infiltration between the popliteal artery and capsule of the knee [IPACK] block, and obturator nerve block). 96 % of respondents declared they traced regional anesthesia modalities in the patient’s health record. Regarding surgical infiltration, they were 80 %, 76 %, and 48 % to answer that the local anesthetic drug, the volume administered, and the precise site, respectively, are traced in the patient’s record.

Pain was immediately evaluated upon post-anesthesia care unit arrival by 29 (91 %) professionals, 100 % of whom formally traced it. For 29 (91 %) respondents, intravenous morphine titration was performed based on a formal (either oral or written) medical prescription; however, written prescription alone was only reported by 6 (19 %) respondents. In this setting, pain was reassessed 5 min after morphine titration for 17 (53 %) respondents. Among nurse anesthetists with >10 years of experience, 8 (50 %) reassessed pain 10 min after morphine titration. Among all professionals 27 (84 %) traced it in the patient’s record. Pain was ultimately evaluated before discharge from the PACU by 31 (97 %) professionals.

Regarding the postoperative period in the surgical ward, 5 (28 %) respondents were unaware of the existence of an institutional pain protocol. Ward nurses reported that they carried out pain assessment at multiple stages: when the patient is admitted to the ward after surgery (100 % assessment rates declared), at each systematic monitoring visit when clinical and vital signs are collected (100 %), before (94 %) and after (100 %) administration of analgesics, before each mobilization (61 %), and before physiotherapy sessions (28 %). Pain assessment traceability was declared by 7 (39 %) nurses overall and in 31 % after administration of analgesics.

Overall, 11 (61 %) nurses in the ward declared they encountered difficulties when managing pain in patients, especially when regional anesthesia was performed. The level of nurse satisfaction with pain management was 7.6/10, with similar results among nurses working in the operating room, the post-anesthesia care unit or the ward.

### 3.2 Rapid training

Phase 3 was dedicated to deployment of rapid training sessions for nurses from the operating room, the post-anesthesia care unit, and the surgical ward. A total of 22 sessions were conducted for the day and night shift teams, including four sessions in the surgical ward (two during day shifts and two during night shifts) and 18 in the operating room.

Twenty-five (25) nurses participated in sessions in the surgical ward (seven nursing assistants, and one nurse student) and 33 nurse anesthetists, one medical doctor in anesthetist, and four student nurse anesthetists participated in sessions in the operating room.

## 4 Results

### 4.1 Patient population

A total of 51 patients were included during Phase 1 (median age 71 years [IQR 66 – 76], 30 women (59 %), median BMI 30.2 kg/m² [22.2 – 42.0]) and 85 patients during Phase 4 (median age 73 years [IQR 65–77], 54 women (64 %), median BMI 29.4 kg/m² [25.9 – 33.6]). Non-response rates for the satisfaction survey were similar (4/51, 8 % in Phase 1; 7/85, 8 % in Phase 4).

### 4.2 Study outcomes

All the results presented below are summarized in Table 1.

**Table 1:** Main and secondary outcomes.

Primary outcome				
		Phase 1 *n* = 51	Phase 4 *n* = 85	
**Recording of the numerical rating scale at the admission**	YES	15 (29.4 %)	41 (48.2 %)	*p* = 0.03 IC95 %: +3.7 – +34 %
	NO	36 (70.6 %)	44 (51.8 %)	
Secondary outcomes				
		Phase 1 *n* = 51	Phase 4 *n* = 85	
**Recording of the numerical rating scale upon arrival in the operating room**	YES	9 (17.6 %)	10 (11.8 %)	*p* = 0.34 IC95 %: −18.9 – +7.3 %
	NO	42 (82.4 %)	75 (88.2 %)	
**Recording of the numerical rating scale upon arrival in the post-anesthesia care unit**	YES	33 (64.7 %)	43 (50.6 %)	*p* = 0.11 IC95 %: −31.1 – +2.9 %
	NO	18 (35.3 %)	42 (49.4 %)	
**Median dose of morphine used in post-anesthesia care unit**		6[6;10]mg	9[5;10]mg	*p* = 0.119*
**Reassessment of pain after an analgesic administration in post-anesthesia care unit**	YES	21 (72.4 %)	38 (86.4 %)	*p* < 0.001 IC95 %: −4.2 – +32.2 %
	NO	8 (27.6 %)	6 (13.6 %)	
**Recording of the numerical rating scale before post-anesthesia care unit discharge**	YES	40 (78.4 %)	67 (78.8 %)	*p* = 0.96 IC95 %: −14.1 – +14.9 %
	NO	11 (21.6 %)	18 (21.2 %)	
**Traceability of on-demand administration of analgesics triggered by a traced numerical rating scale >3/10 during H24**	YES	32 (62.7 %)	75 (88.2 %)	*p* < 0.001 IC95 %: +11.2 – +39.8 %
	NO	19 (37.3 %)	10 (11.8 %)	
**Reassessment of pain after an analgesic administration during first 24 h**	YES	6 (19.4 %)	10 (25 %)	*p* = 0.57 IC95 %: −13.4 – +24.6 %
	NO	25 (80.7 %)	30 (75 %)	
**Patient’s satisfaction score**	0 to 10 scale	9[8;10]	8[8;10]	*p* = 0.249

⁎ Note Ha μ1 < μ4.

### 4.3 Primary outcome

Recording of numerical rating scale assessment at admission to the orthopedic ward statistically significantly increased from 15/51 (29.4 %) in Phase 1 to 41/85 (48.2 %) in Phase 4 (*p* = 0.03). This represents an absolute increase of +18.8 percentage points. IC95 % [+3,7 – 34 %]

### 4.4 Secondary outcomes

At arrival in the operating room, recording of the numerical rating scale remained low and did not differ significantly between Phase 1 (9/51, 17.6 %) and Phase 4 (10/85, 11.8 %; absolute difference −5.8 percentage points, 95 % CI [−18.9 to +7.3]; *p* = 0.34).

Similarly, numerical rating scale traceability upon arrival in the post-anesthesia care unit showed a non-significant decrease from Phase 1 (33/51, 64.7 %) to Phase 4 (43/85, 50.6 %; absolute difference −14.1 percentage points, 95 % CI [−31.1 to +2.9]; *p* = 0.11). Recording of the numerical rating scale before post-anesthesia care unit discharge remained stable between phases (40/51, 78.4 % vs. 67/85, 78.8 %; absolute difference +0.4 percentage points, 95 % CI [−14.1 to +14.9]; *p* = 0.96).

The median dose of morphine administered in the post-anesthesia care unit showed a non-significant increase between Phase 1 (6 mg [6;10]) and Phase 4 (9 mg [5;10]; *p* = 0.119).

During the first 24 postoperative hours, traceability of on-demand analgesic administration triggered by a documented numerical rating scale >3/10 increased from 32/51 (62.7 %) in Phase 1 to 75/85 (88.2 %) in Phase 4, representing an absolute increase of +25.5 percentage points (95 % CI [+11.2 to +39.8]; *p* < 0.001).

Pain reassessment after analgesic administration during the first 24 h remained unchanged (Phase 1: 6/31, 19.3 %; Phase 4: 10/40, 25.0 %; absolute difference +5.7 percentage points, 95 % CI [−13.4 to +24.6]; *p* = 0.57).

Median patient satisfaction scores remained high and did not differ significantly between Phase 1 (9 [8-10]) and Phase 4 (8 [8-10]; *p* = 0.22).

## 5 Discussion

This study assessed the impact of a nurse anesthetist student-led training program on perioperative pain management practices for patients undergoing total knee replacement surgery at a French university hospital. Overall, the intervention improved several aspects of pain assessment traceability and pain management practices, particularly in the surgical ward, while effects were more limited in the operating room and post-anesthesia care unit. These findings support the role of targeted, context-adapted educational interventions in enhancing perioperative pain care.

The primary outcome showed a meaningful improvement in pain assessment traceability upon admission to the orthopedic surgery ward. This suggests that ward nurses integrated the training effectively into their daily workflow, consistent with previous studies showing that structured educational interventions improve adherence to pain assessment protocols. Importantly, this improvement occurred despite unchanged staffing levels or documentation tools, reinforcing the idea that training alone can modify professional practices when barriers remain relatively low. This finding aligns with previous studies that have demonstrated the effectiveness of educational interventions in improving pain management practices (Grommi et al., 2023).

Conversely, preoperative pain assessment traceability in the operating room remained very low and did not improve after training. This likely reflects deeper structural barriers: notably, the absence of a clearly identified space for documenting preoperative pain in the operating room record. Similar findings have been described in the literature, where traceability is strongly dependent on the availability and usability of documentation interfaces rather than on staff knowledge alone. (Dalton et al., 2001; Azzolini et al., 2019; Ebbers et al., 2022) The post-anesthesia care unit showed a comparable pattern: although staff reported frequent assessments, documentation remained inconsistent – again suggesting system-level constraints rather than a lack of awareness or willingness to comply.

Beyond documentation, several indicators of pain management behaviors improved after training. Pain reassessment following analgesic administration increased, particularly in the post-anesthesia care unit, suggesting greater vigilance and adherence to good clinical practice. The higher morphine doses administered in Phase 4 could reflect more appropriate titration in response to documented pain, although over-treatment cannot be excluded. Without individual pain trajectories, it is difficult to determine whether this represents better responsiveness to patient needs or potential overtreatment. This highlights the need for future studies incorporating real-time pain intensity data and clinical context when interpreting analgesic use.

The increase in on-demand analgesic administration during the first postoperative day further suggests heightened attentiveness to patient-reported pain. However, the persistently low rates of pain reassessment after rescue analgesia on the ward indicate that some aspects of the protocol remain insufficiently integrated despite training. Similar gaps have been reported in multicenter audits, emphasizing that reinforcement strategies and system-level solutions – such as mandatory fields in medical records – may be necessary to sustain improvement. (Brant et al., 2017)

Patient satisfaction remained high, although a small decrease was observed after training. Several hypotheses may explain this finding. First, improved documentation and more systematic assessments may heighten patient expectations regarding pain relief. Second, the training program primarily targeted professional practices rather than patient communication, which is known to influence satisfaction. Prior studies have shown that patient education on analgesia plans and setting realistic expectations often has a strong impact on satisfaction, sometimes more than clinical process improvements alone. External factors such as case-mix variations may also have contributed. (Chou and Lin, 2011; Innis et al., 2004; Mankelow et al., 2022)

This study has limitations. Its single-center, before–after design without randomization exposes it to potential confounding factors and cannot fully rule out a Hawthorne effect : nurses aware of being observed may temporarily modify their behavior. The relatively small sample sizes also limit statistical power. Structural limitations of the documentation system may have constrained the potential impact of the training. Finally, the extremely low use of regional anesthesia in both phases (4 cases in Phase 1 and 4 in Phase 4) prevented any meaningful evaluation of regional anesthesia related practices or their influence on postoperative pain outcomes.

Overall, this study highlights that while targeted training can meaningfully improve perioperative pain management practices, especially in settings with modifiable behavioral barriers, sustained improvements in documentation and traceability likely require parallel structural changes in workflow organization and medical records.

## 6 Conclusion

This nurse anesthetist student–led training program improved traceability and postoperative pain management practices in the surgical ward, although its impact in the operating room and post-anesthesia care unit remained limited by documentation constraints. These improvements are clinically meaningful, as systematic pain assessment supports safer and more effective analgesia which could lead to a better patient’s satisfaction. The single-center before–after design and small sample size represent important limitations. Regular refresher training and structural improvements to medical records – such as the transition to digital patient records – are needed to sustain progress. Further multicenter studies should evaluate how combined educational and system-level interventions can enhance perioperative pain management.

## Ethics approval

The study was approved under number 2022-CF-073 by the local Ethics Committee at Clermont Ferrand university hospital (IRB00013412, “CHU de Clermont Ferrand IRB #1″), which waived informed consent requirements.

## Consent for publication

Not applicable.

## Data availability

The research data are available upon request to the corresponding author. Deidentified data will be available at time of publication to researchers who provide a methodologically sound and ethically approved proposal, for any purpose of analysis. A data use agreement will be required before the release of participant data and institutional review board approval as appropriate.

## Funding

None.

## Patient and public involvement

Patients and/or the public were not involved in the design, reporting, or dissemination plans of this research.

## CRediT authorship contribution statement

**Elie Guillen:** Writing – review & editing, Writing – original draft, Visualization, Validation, Investigation, Formal analysis, Data curation. **Matthieu Jabaudon:** Writing – review & editing, Writing – original draft, Supervision, Resources, Project administration, Methodology, Funding acquisition, Formal analysis, Conceptualization. **Marc Garnier:** Writing – review & editing, Visualization, Validation. **Cathy Paulet:** Supervision, Project administration, Methodology, Conceptualization. **Cécile Vermeersch:** Supervision, Resources, Project administration, Methodology, Conceptualization.

## Declaration of competing interest

The authors declare that they have no known competing financial interests or personal relationships that could have appeared to influence the work reported in this paper.

## Appendix Supplementary materials

Image, application 1
