# Supplementary material for: Impact of a nurse anesthetist student–led training program on perioperative pain management in total knee replacement: A prospective before and after study
Source: Int J Nurs Stud Adv. 2026 Jan 27;10:100495. doi: 10.1016/j.ijnsa.2026.100495 (PMC12925520; doi:10.1016/j.ijnsa.2026.100495)
Supplement: Supplementary file 1 [file mmc1.zip › CU - QuickFormationPainManagement.docx]

| 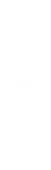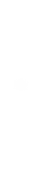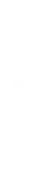  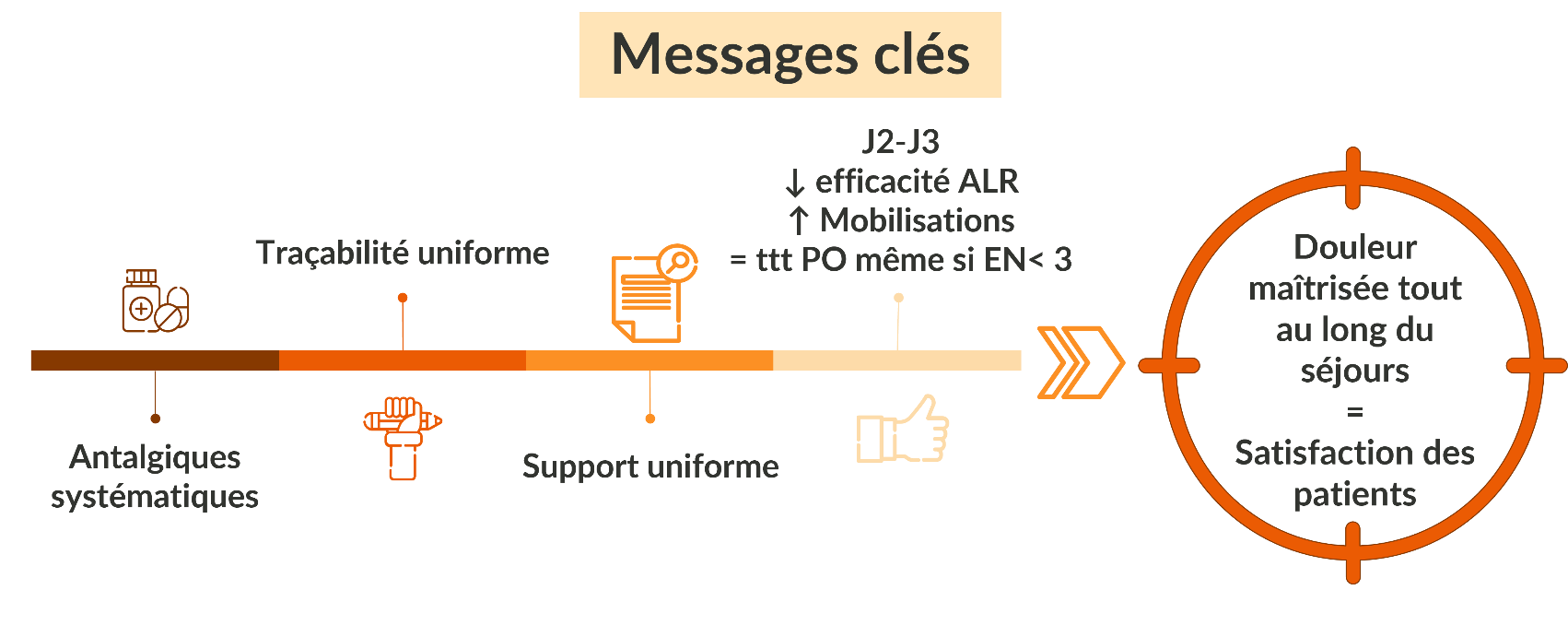  **J2/J3 ↓local anesthesia effects ↑mobilizations = systematics painkillers even if EN < 3**  **Take home messages**  **Unified record**  **Unified traceability**  **Systematics Painkillers** | 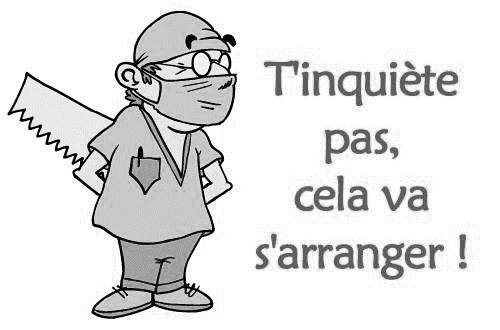  Relax ! Everything will be all right !  Thanks for your help | 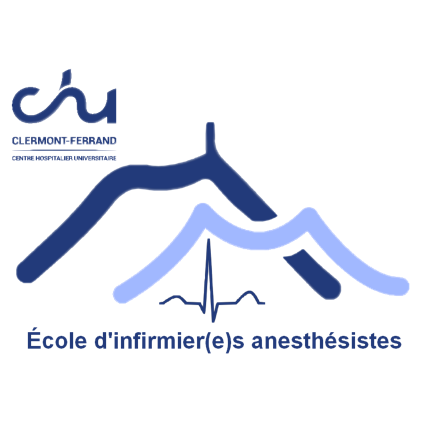  Orthopedic Surgery  Pain Memo (Care unit)  Nursing anesthetist students  Class of 2021-2023  Clermont-Ferrand University Hospital |
| --- | --- | --- |
| 1. **KNOW**   Postoperative pain is :   - Acute - Predictable - Foreseeable - **Quantifiable**      1. **EVALUATE**   **SELF-EVALUATION**  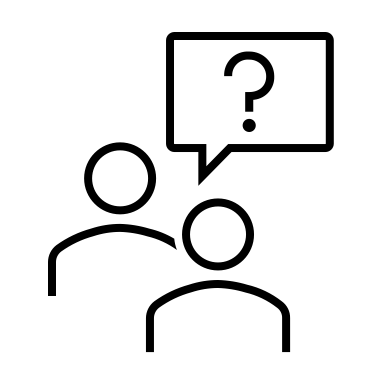   - Most used - Use EN in priority - For adults able to communicate   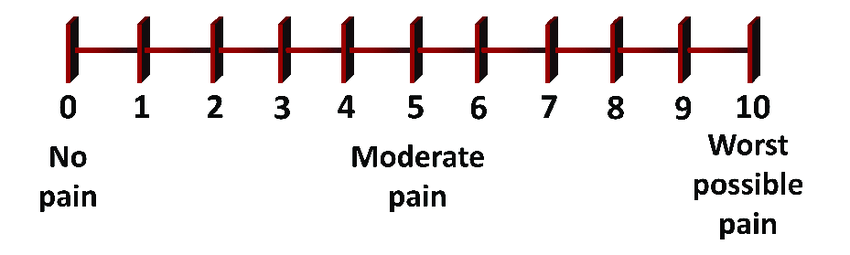  **HETERO-EVALUATION**   - When self-evaluation is not possible - « Algoplus® » scale - Use the rating grid - Always use the same rating scale | 1. **CURE**   **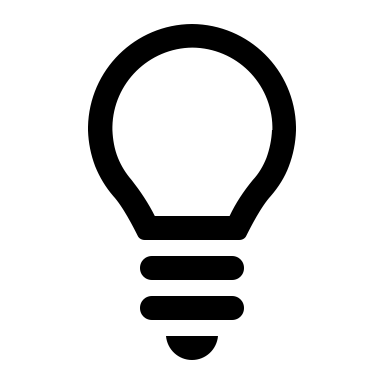**  **Watch for the protocol “PTG/PUC”**  **OBJECTIVE :**  EARLY MOBILISATION  MULTIMODAL ANALGESIA  PAINKILLERS  LOCAL ANESTHESIA   - **Everyday (unless contraindications)**   **Paracetamol IV :**   - Moderate power - Potentiates NSAIDs and opioids - Action within 20 to 30 min - Effective period : 4 to 6 hours - Hepatotoxicity   **Ketoprofen IV :**   - Powerful ++ on acute and traumatic pain - Action within 15 min - Effective period : 4 hours - Renal and gastroduodenal toxicity (PPI)   **Nefopam IV :**   - Moderate power - Potentiates NSAIDs and morphine - Action within 20 to 60 min - Effective period : 5 to 6 hours - Urinary retention, glaucoma, seizure - **In rescue**   **Actiskenan : Oral morphine**   - Powerful +++ - Intense pain, resistant to other analgesics - Action within 15 to 30 min - Effective period 4 to 6 hours - Respiratory depression, urinary retention, sedation | **4. MONITOR**  **TRACEABILITY +++**  **WHEN ?**   - Upon admission in the care unit - When leaving/returning from the OR - Before using any painkiller - **Reassess after analgesic administration** - Before/after each mobilization   **WHERE ?**   - Summary of care record (cf HAS) - Post-op monitoring sheet for 48 hours - Care plan diagram   **HOW ?**   - Name the used scale = EN - Graduation = …/10 - Localization - Time   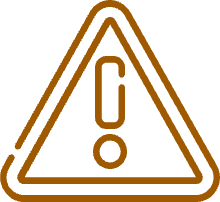  UNRECORDED = NOT DONE  **Local Anesthesia**   - Sensory bloc = cold test - no motor block or very mild (except in spinal anesthesia)   Sensory block effect = 48h max  Report any issues |
