# Supplementary material for: Impact of a nurse anesthetist student–led training program on perioperative pain management in total knee replacement: A prospective before and after study
Source: Int J Nurs Stud Adv. 2026 Jan 27;10:100495. doi: 10.1016/j.ijnsa.2026.100495 (PMC12925520; doi:10.1016/j.ijnsa.2026.100495)
Supplement: Supplementary file 1 [file mmc1.zip › OR - QuickFormationPainManagement.docx]

| 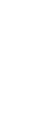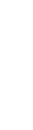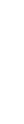  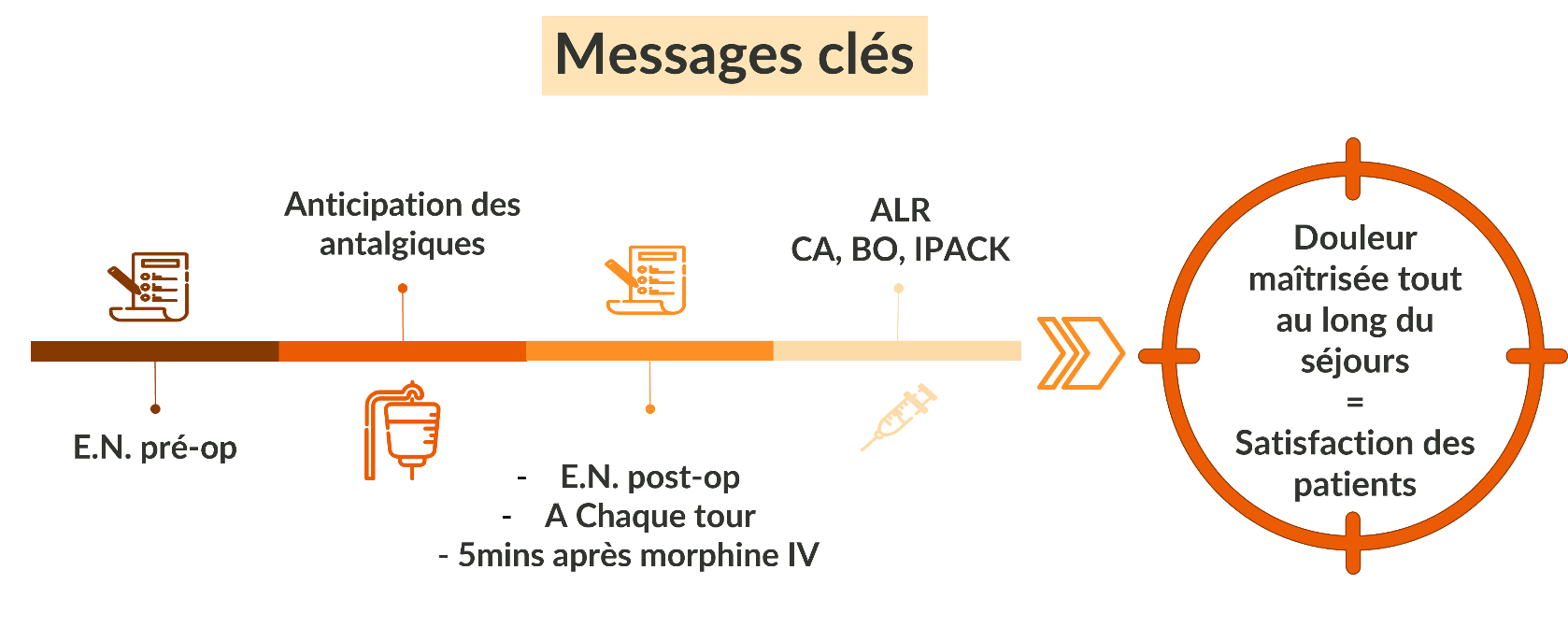  **Postoperative EN**  **At each monitoring**  **5min after morphine**  **Local anesthesia**  **AC, ONB, IPACK**  **Painkillers’ anticipation**  **Preoperative evaluation**  **Take home messages** | 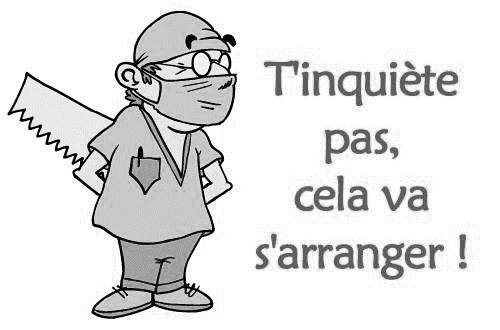  Relax ! Everything will be all right !  Thanks for your help ! | 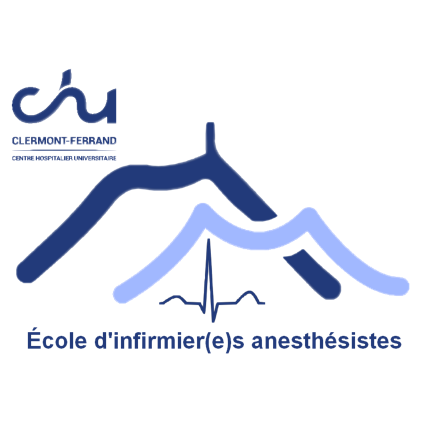  Orthopedic Surgery  Pain MEMO (operating theatre)  Nurse anesthetist students  Class of 2021-2023  Clermont-Ferrand University Hospital |
| --- | --- | --- |
| 1. **KNOW**   Postoperative pain is :   - Acute - Predictable - Foreseeable - **Quantifiable**      1. **EVALUATE**   **SELF-EVALUATION**  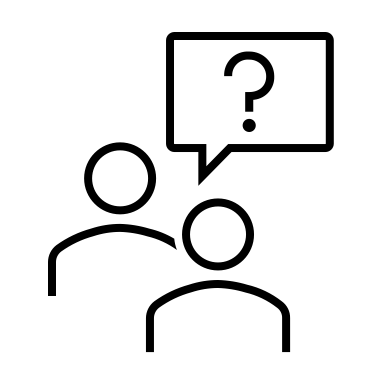   - Most used - Use EN in priority - For adults able to communicate   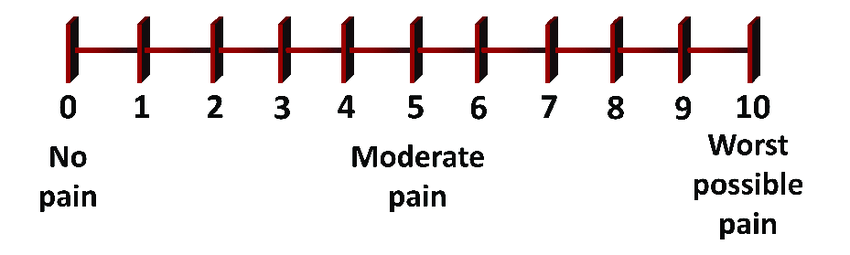  **HETERO-EVALUATION**   - When self-evaluation is not possible - « Algoplus® » scale - Use the rating grid - Always use the same rating scale | 1. **CURE**   **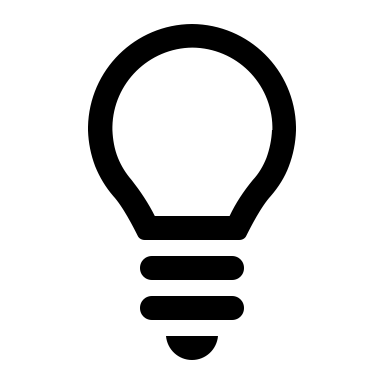**  **Watch for the protocol “PTG/PUC”**  **OBJECTIVE :**  EARLY MOBILISATION  MULTIMODAL ANALGESIA  PAINKILLERS  LOCAL ANESTHESIA   - **Everyday (unless contraindications)**   **Paracetamol IV :**   - Moderate power - Potentiates NSAIDs and opioids - Action within 20 to 30 min - Effective period : 4 to 6 hours - Hepatotoxicity   **Ketoprofen IV :**   - Powerful ++ on acute and traumatic pain - Action within 15 min - Effective period : 4 hours - Renal and gastroduodenal toxicity (PPI)   **Nefopam IV :**   - Moderate power - Potentiates NSAIDs and morphine - Action within 20 to 60 min - Effective period : 5 to 6 hours - Urinary retention, glaucoma, seizure - **In rescue**   **Morphine IV :**   - Powerful +++ - Intense pain, resistant to other analgesics - Action within 5min - Effective period : 3 to 4 hours - Respiratory depression, urinary retention, sedation | **4. MONITOR**  **TRACEABILITY +++**  **WHEN ?**   - Upon admission in the OR - Upon admission in recovery room - At each monitoring - **Reassess after analgesic administration** - When leaving recovery room   **WHERE ?**   - Anesthesia record sheets or software   **HOW ?**   - Name the used scale = EN - Graduation = …/10 - Localization - 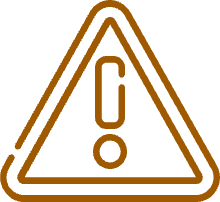Time   UNRECORDED = NOT DONE  **Local Anesthesia**   - In the CHU’s protocol : - Obturator Nerve block (ON) - Adductor Canal block (AC) - IPACK block - Traceability of local anesthesia + surgical infiltration - Objective : no motor block or very mild (except in spinal anesthesia) - Monitoring for signs of local anesthetic toxicity |
