# Supplementary material for: Impact of a nurse anesthetist student–led training program on perioperative pain management in total knee replacement: A prospective before and after study
Source: Int J Nurs Stud Adv. 2026 Jan 27;10:100495. doi: 10.1016/j.ijnsa.2026.100495 (PMC12925520; doi:10.1016/j.ijnsa.2026.100495)
Supplement: Supplementary file 1 [file mmc1.zip › Quick Formation Pain (Ward).pptx]

## Slide 1
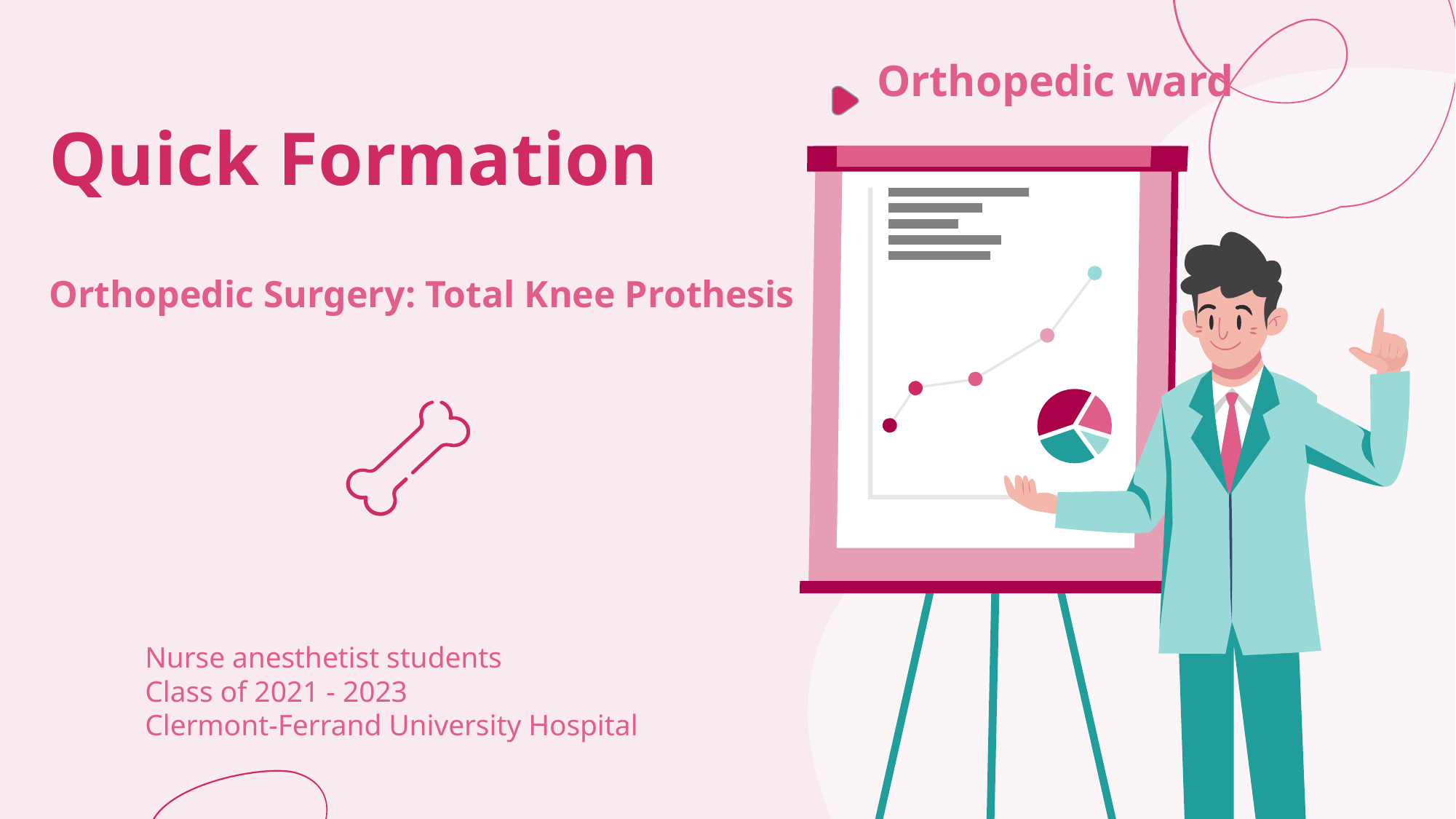

Orthopedic ward
# Quick Formation
Orthopedic Surgery: Total Knee Prothesis
Nurse anesthetist students
Class of 2021 - 2023
Clermont-Ferrand University Hospital

## Slide 2
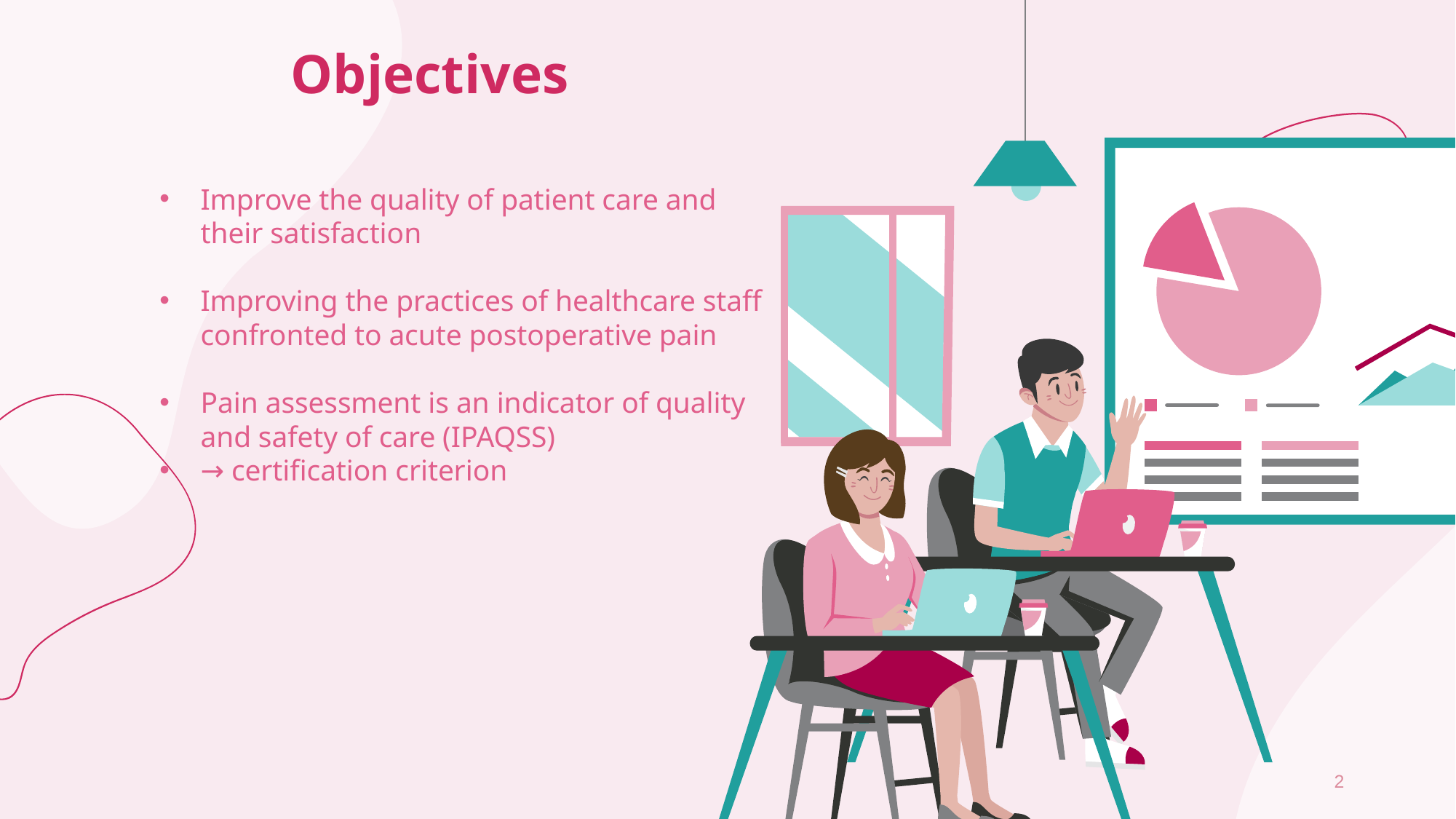

# Objectives
Improve the quality of patient care and their satisfaction
Improving the practices of healthcare staff confronted to acute postoperative pain
Pain assessment is an indicator of quality and safety of care (IPAQSS)
→ certification criterion
2

## Slide 3
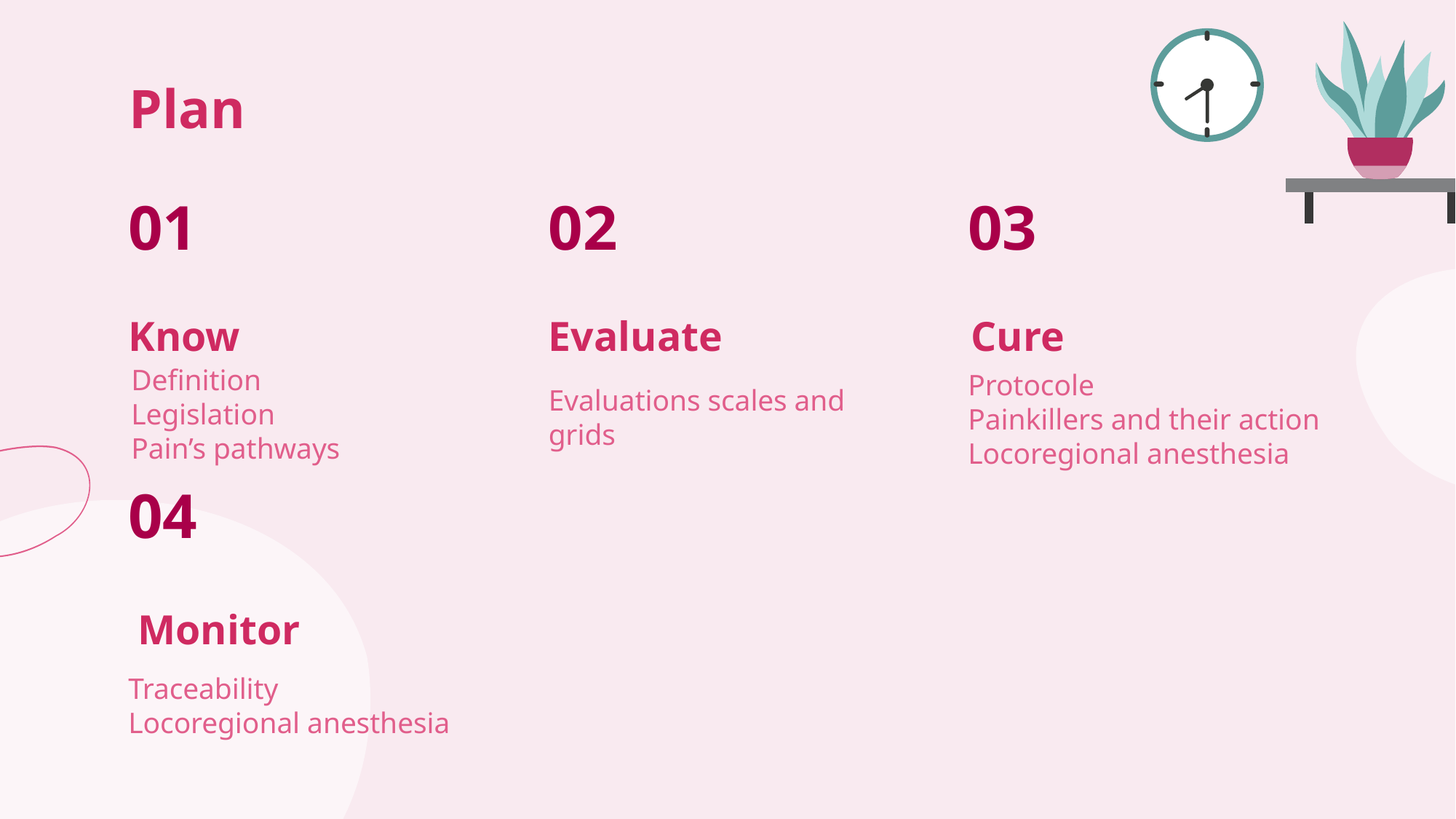

Plan
# 01
02
03
Know
Evaluate
Cure
Definition
Legislation
Pain’s pathways
Protocole
Painkillers and their action
Locoregional anesthesia
Evaluations scales and grids
04
Monitor
Traceability
Locoregional anesthesia

## Slide 4
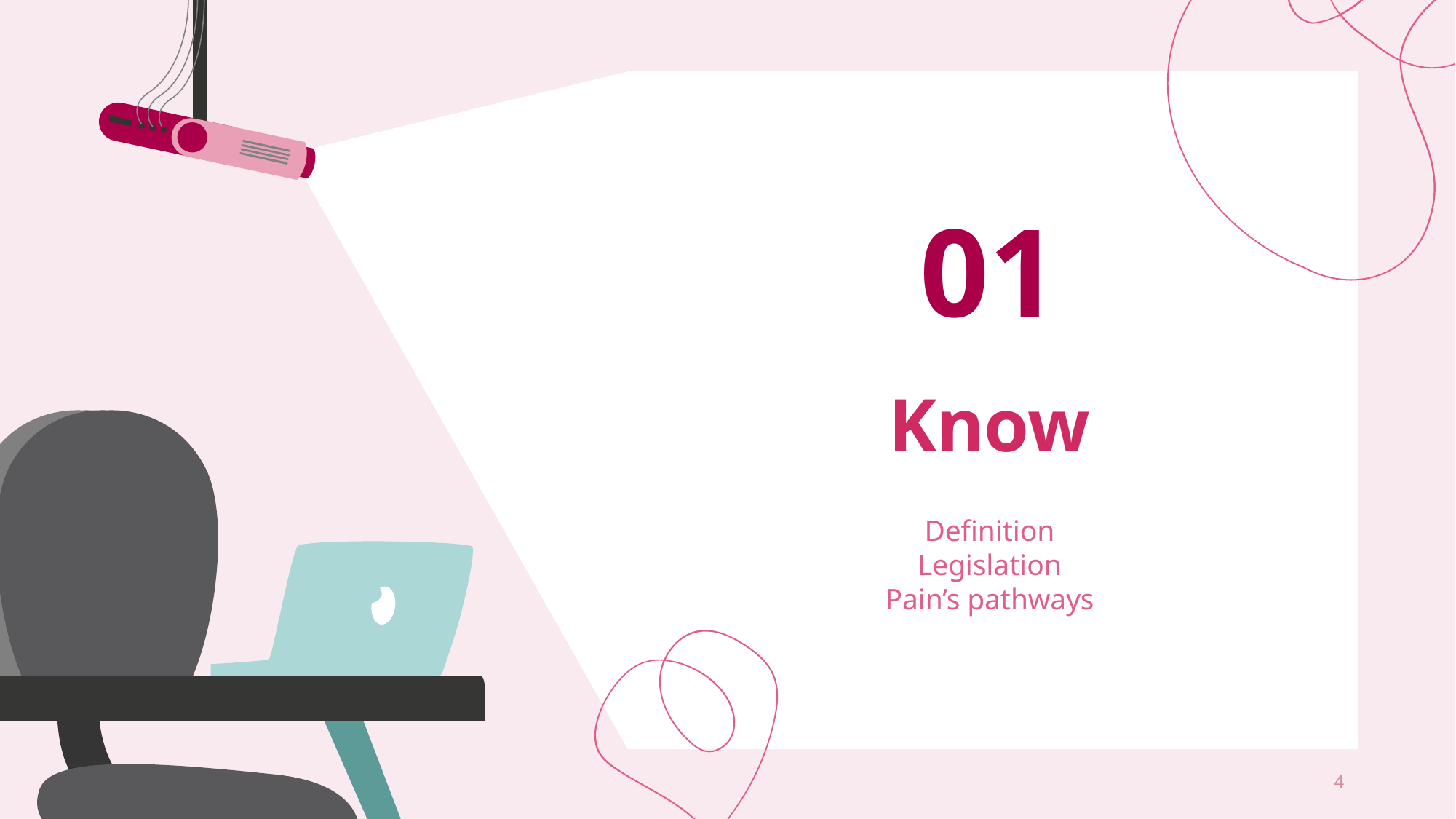

01
# Know
Definition
Legislation
Pain’s pathways
4

## Slide 5
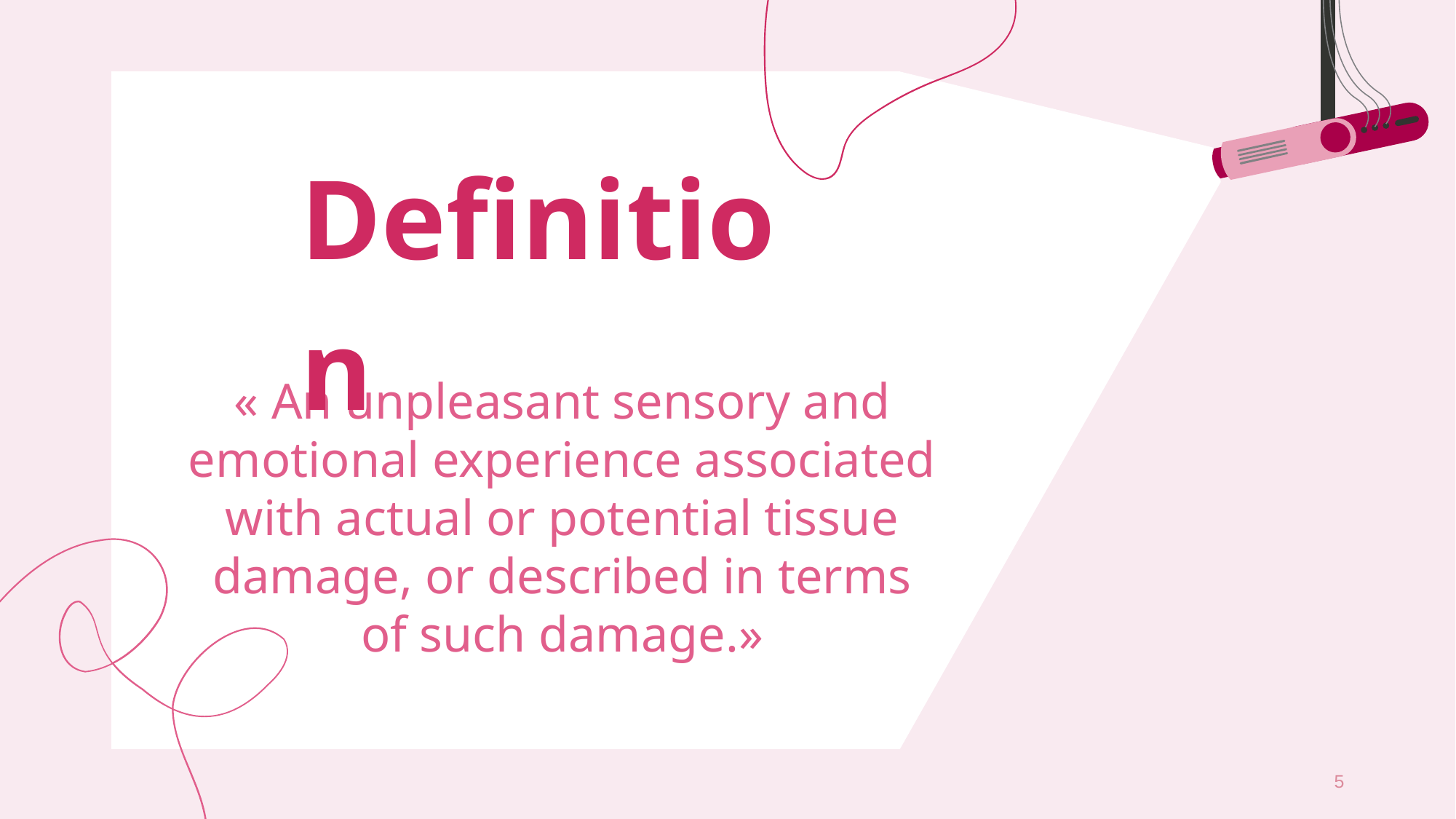

Definition
# « An unpleasant sensory and emotional experience associated with actual or potential tissue damage, or described in terms of such damage.»
5

## Slide 6
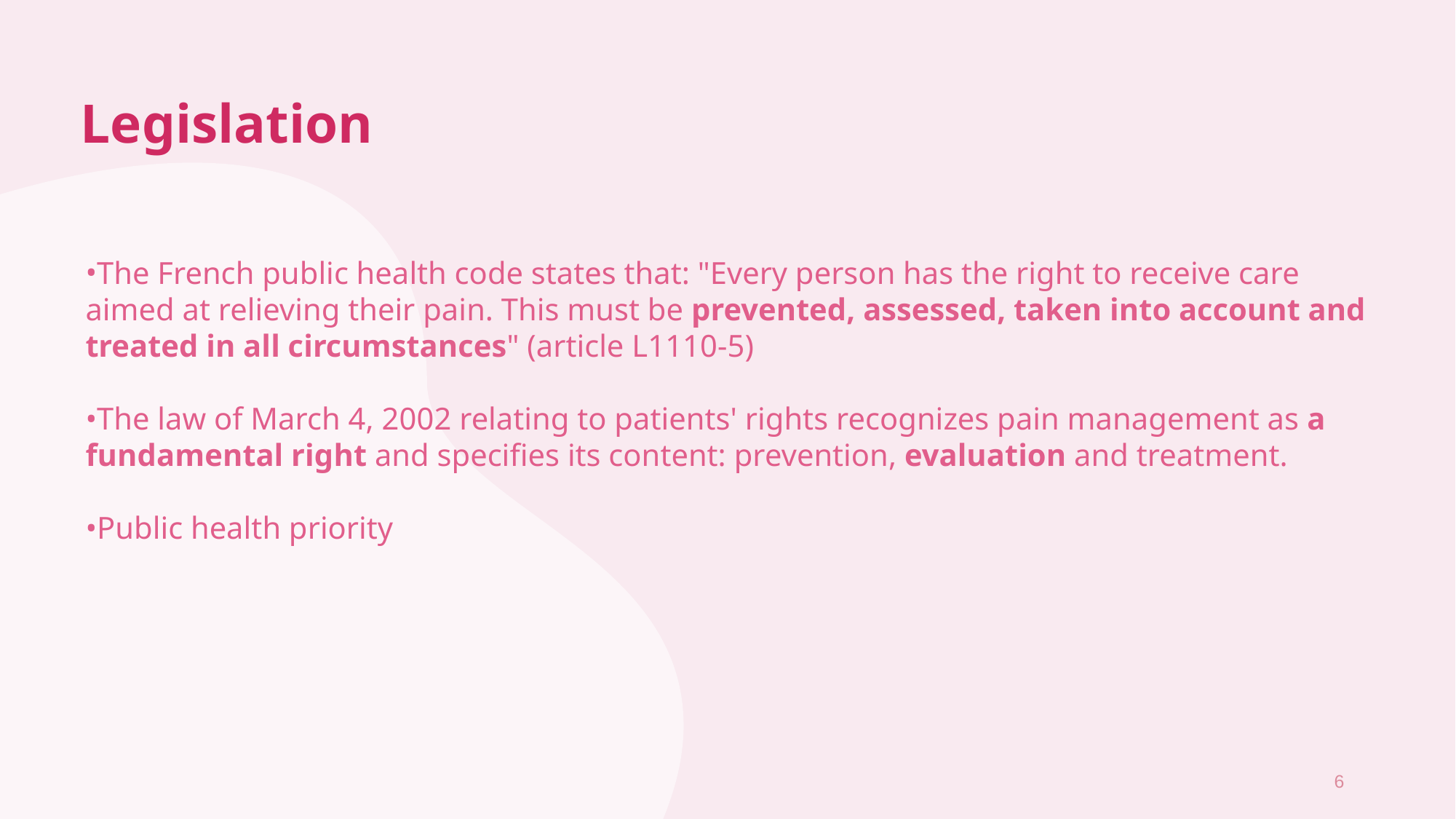

# Legislation
The French public health code states that: "Every person has the right to receive care aimed at relieving their pain. This must be prevented, assessed, taken into account and treated in all circumstances" (article L1110-5)
The law of March 4, 2002 relating to patients' rights recognizes pain management as a fundamental right and specifies its content: prevention, evaluation and treatment.
Public health priority
6

## Slide 7
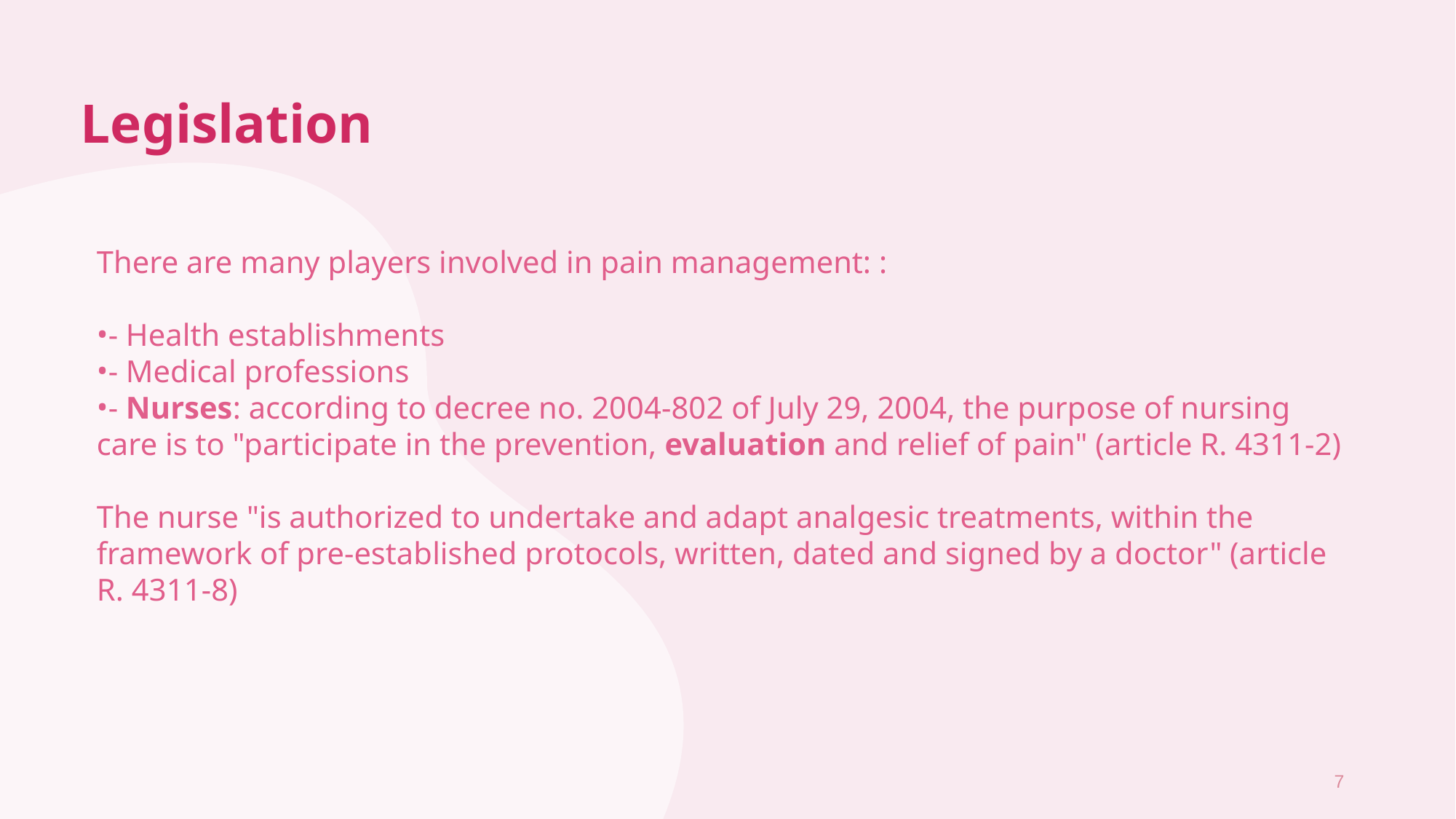

# Legislation
There are many players involved in pain management: :
- Health establishments
- Medical professions
- Nurses: according to decree no. 2004-802 of July 29, 2004, the purpose of nursing care is to "participate in the prevention, evaluation and relief of pain" (article R. 4311-2)
The nurse "is authorized to undertake and adapt analgesic treatments, within the framework of pre-established protocols, written, dated and signed by a doctor" (article R. 4311-8)
7

## Slide 8
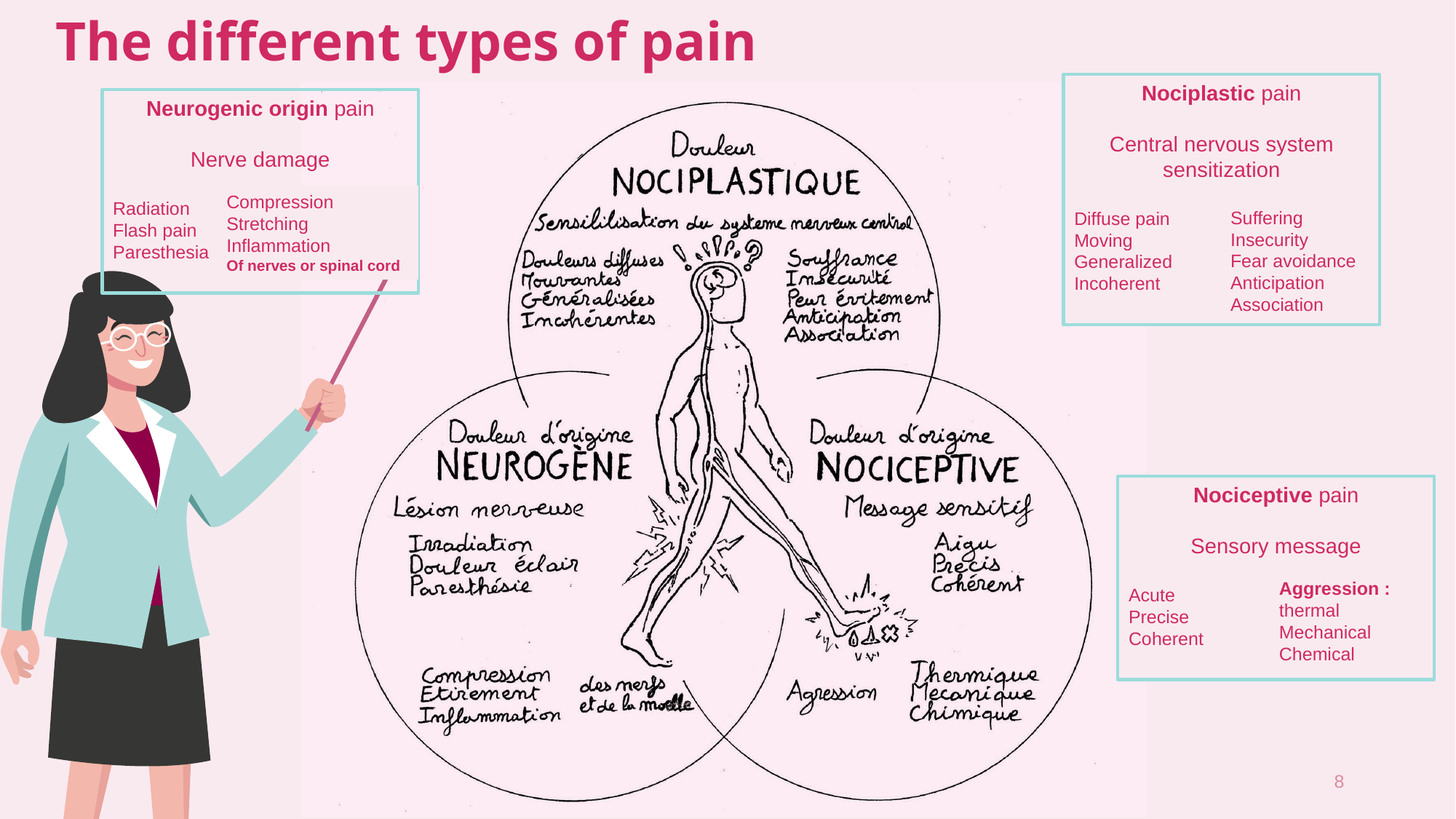

# The different types of pain
Nociplastic pain
Central nervous system sensitization
Diffuse pain
Moving
Generalized
Incoherent
Suffering
Insecurity
Fear avoidance
Anticipation
Association
Neurogenic origin pain
Nerve damage
Radiation
Flash pain
Paresthesia
Compression
Stretching
Inflammation
Of nerves or spinal cord
Nociceptive pain
Sensory message
Acute
Precise
Coherent
Aggression :
thermal
Mechanical
Chemical
8

## Slide 9
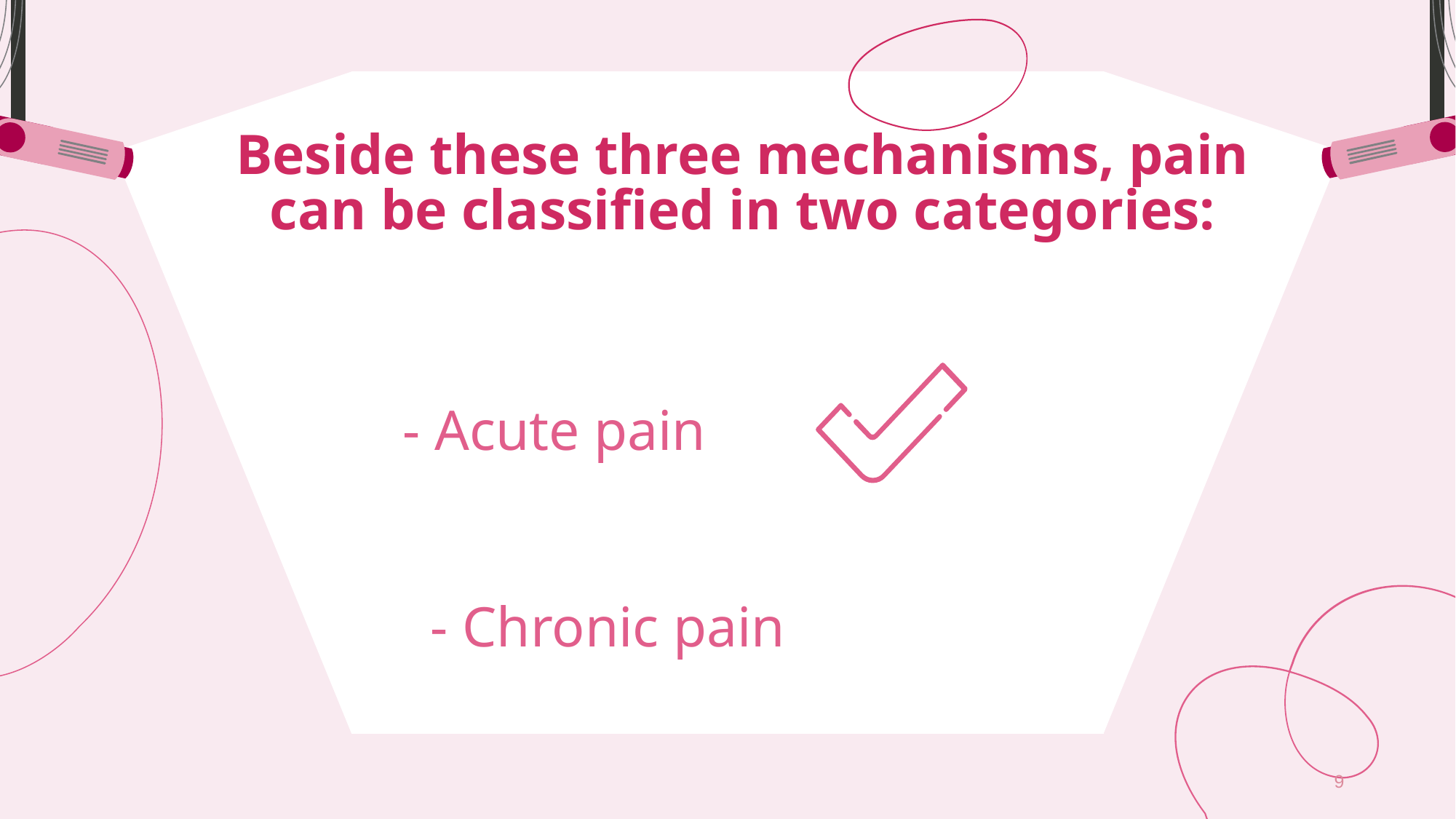

# Beside these three mechanisms, pain can be classified in two categories:
- Acute pain
- Chronic pain
9

## Slide 10
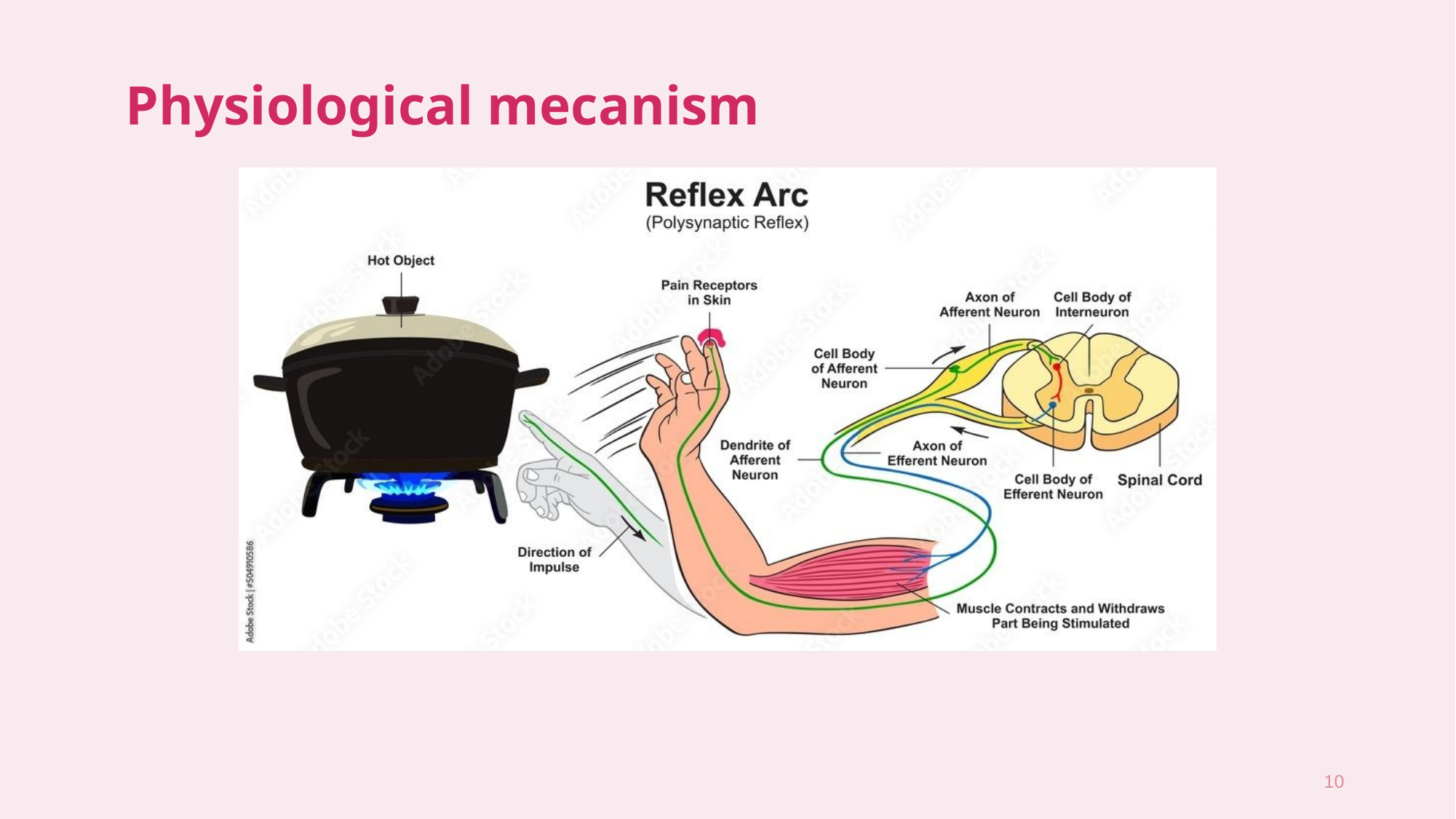

# Physiological mecanism
10

## Slide 11
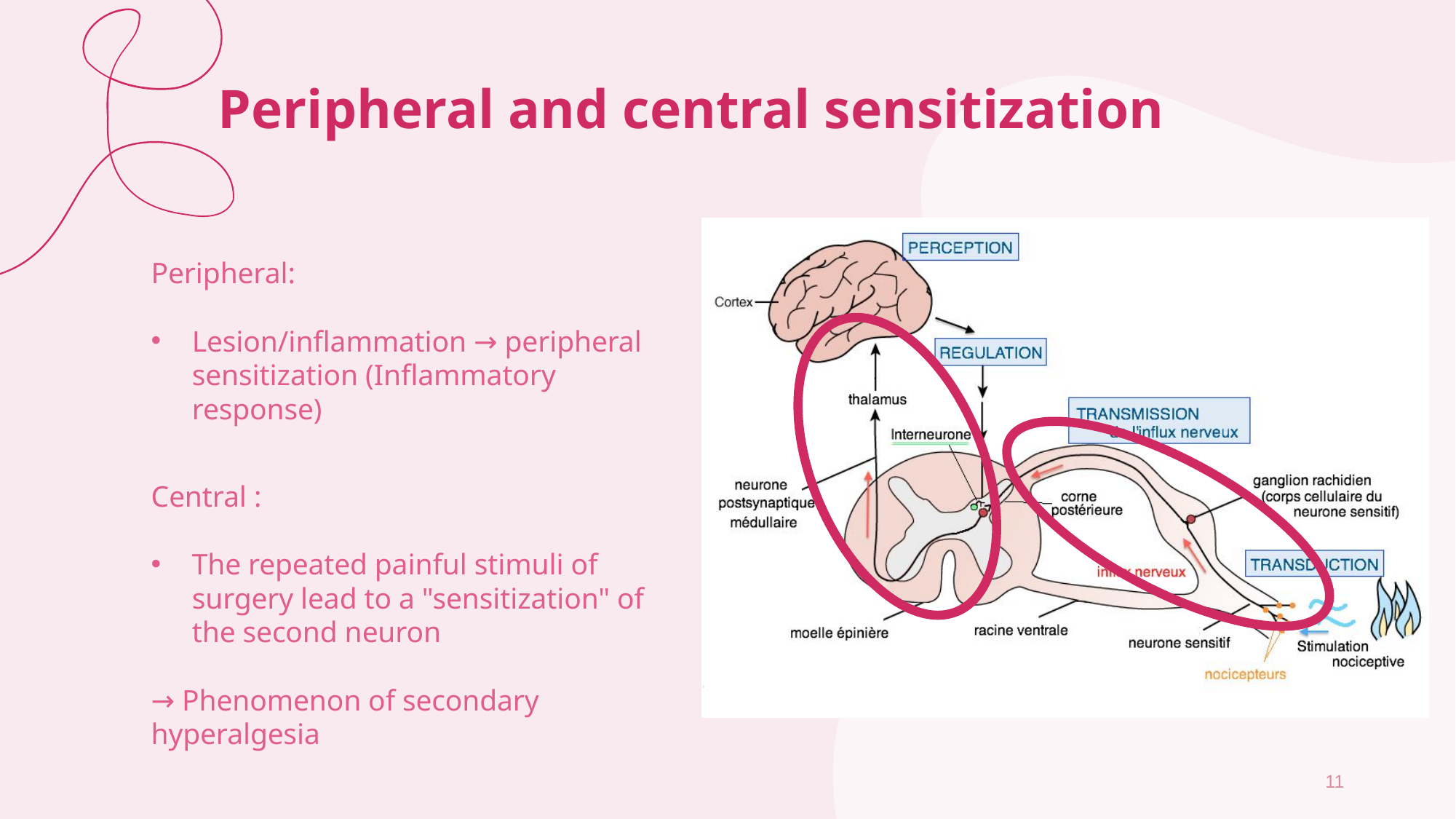

# Peripheral and central sensitization
Peripheral:
Lesion/inflammation → peripheral sensitization (Inflammatory response)
Central :
The repeated painful stimuli of surgery lead to a "sensitization" of the second neuron
→ Phenomenon of secondary hyperalgesia
11

## Slide 12
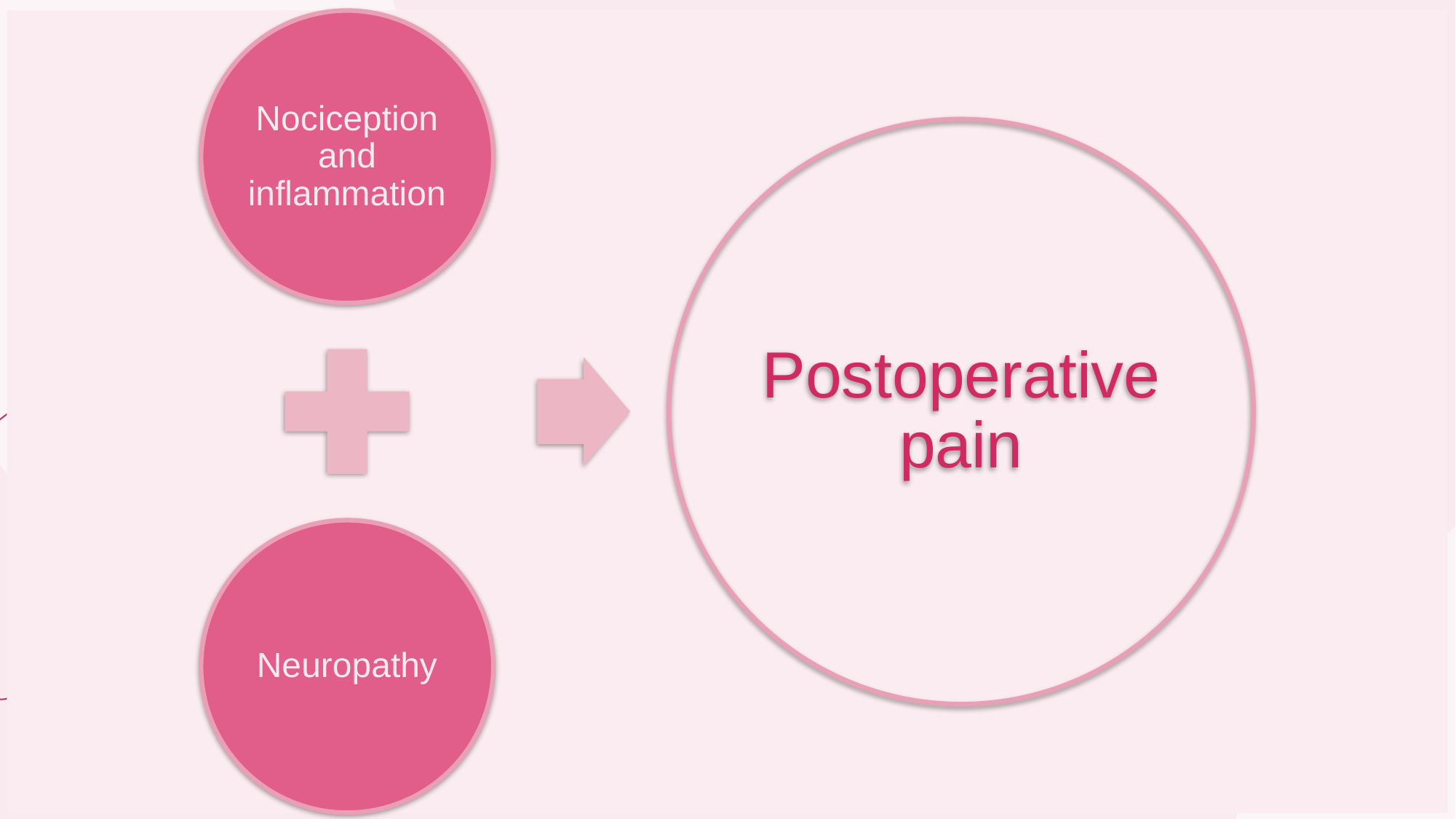

# Pain and surgery
Postoperative pain is provoqued by:
Tissue trauma  Inflammatory reaction
Peripheral nerve injury
Secondary hyperalgesia
This pain is :
Acute
Predictable
Foreseeable
Quantifiable
12

## Slide 13
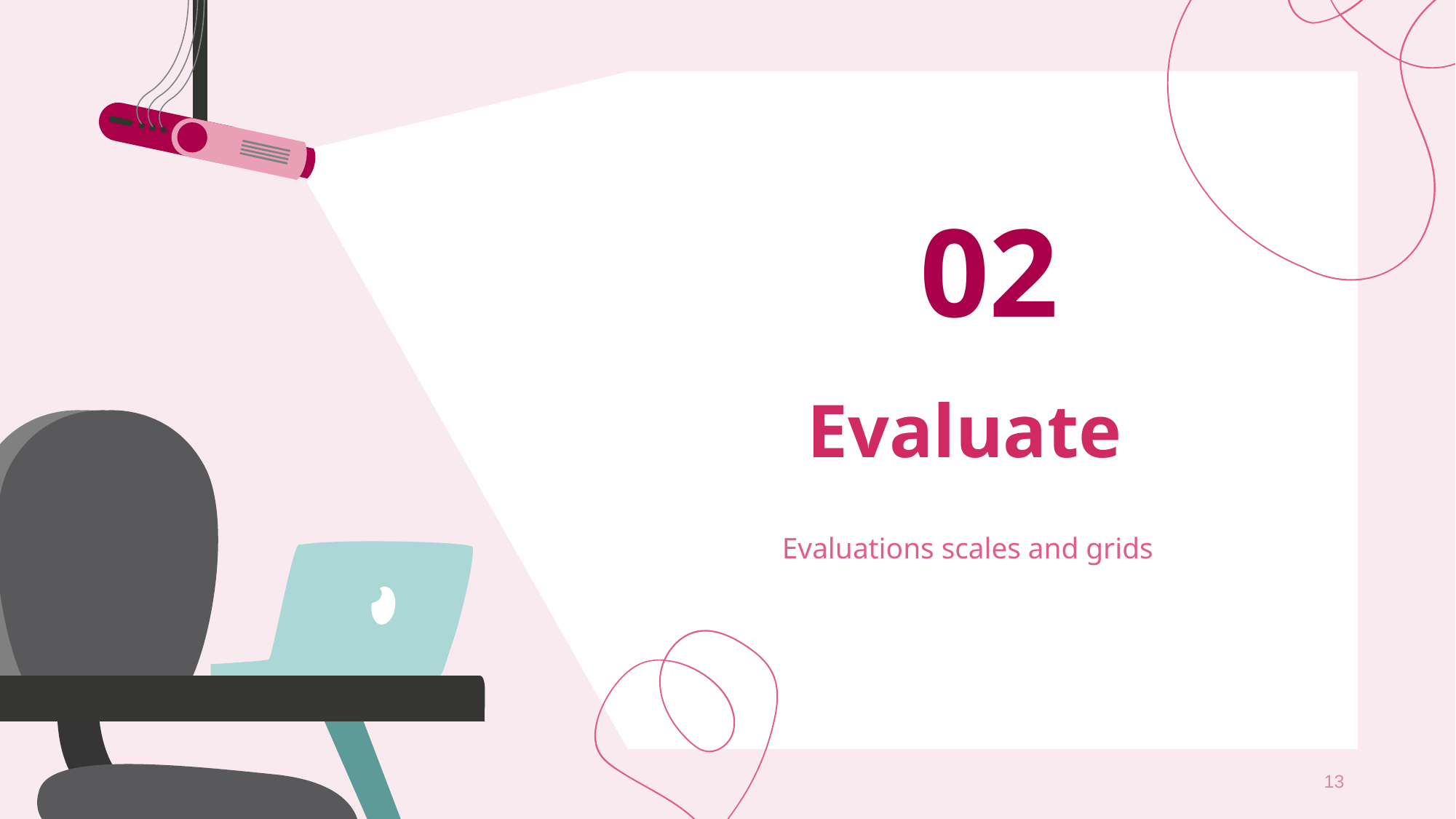

02
# Evaluate
Evaluations scales and grids
13

## Slide 14
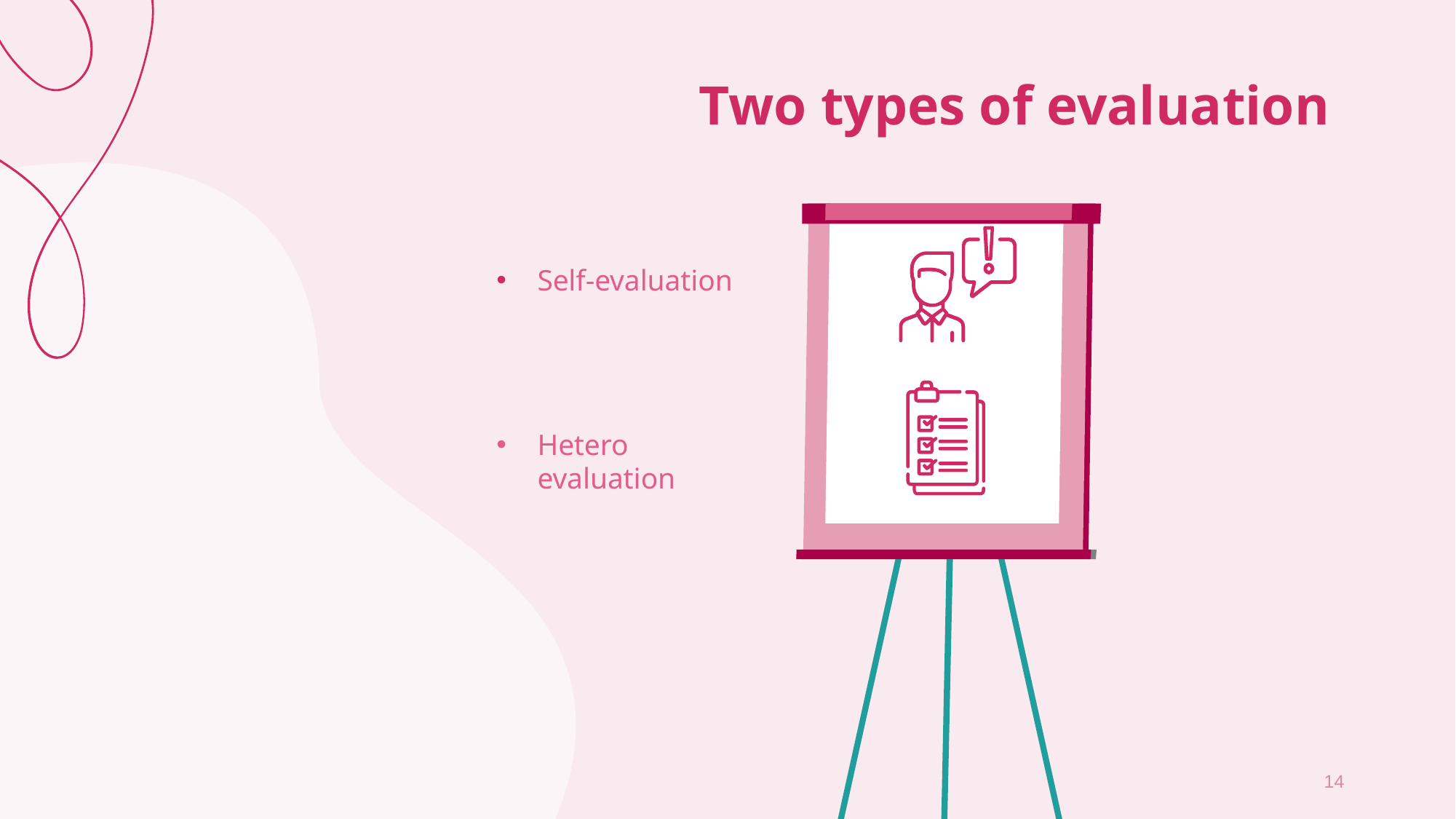

# Two types of evaluation
Self-evaluation
Hetero evaluation
14

## Slide 15
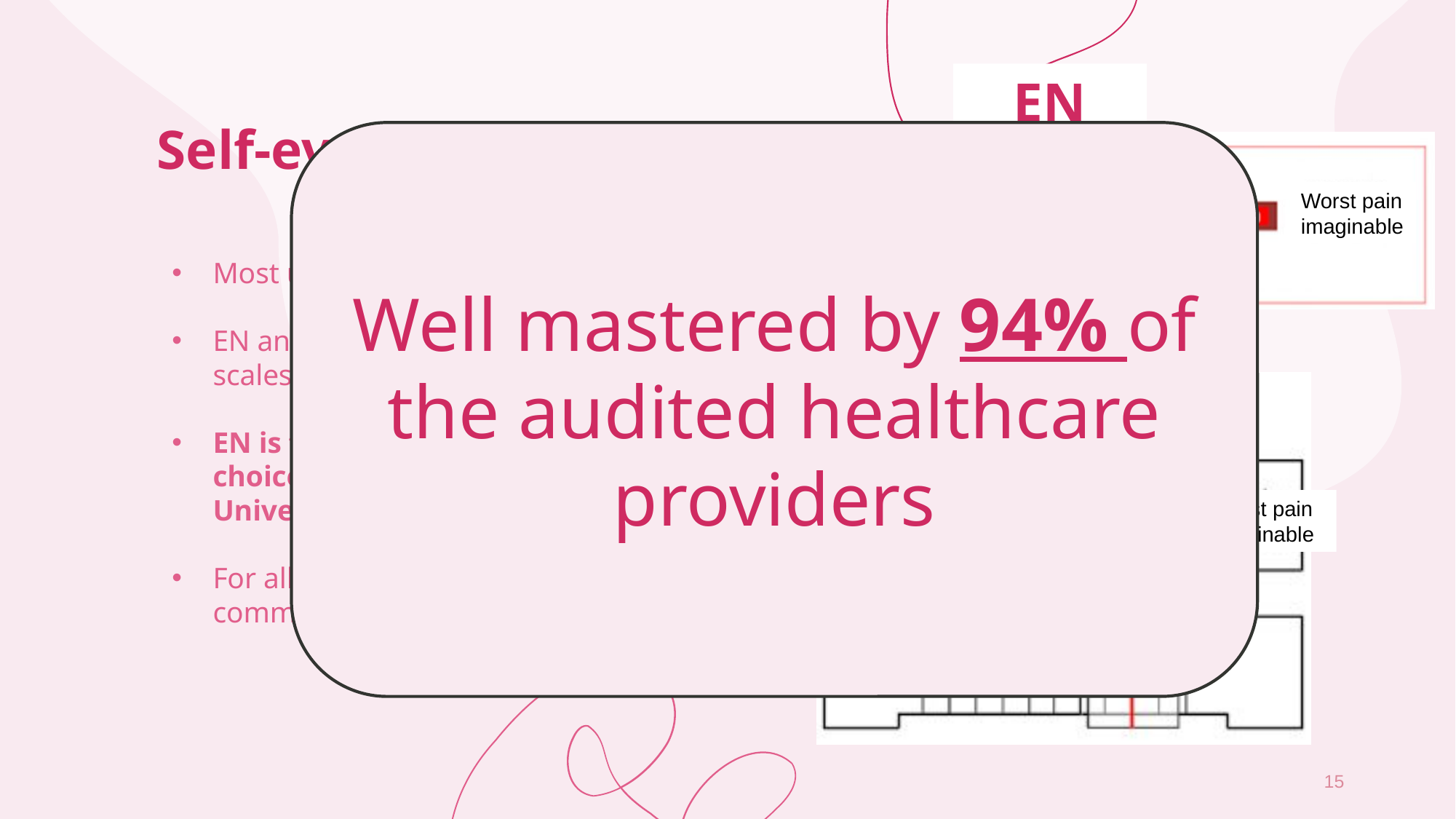

EN
# Self-evaluation
Well mastered by 94% of the audited healthcare providers
Worst pain imaginable
No pain
Most used
EN and EVA are most known scales
EN is the preferred institutional choice of the Clermont-Ferrand University Hospital
For all adults able to communicate
Worst pain imaginable
No pain
15

## Slide 16
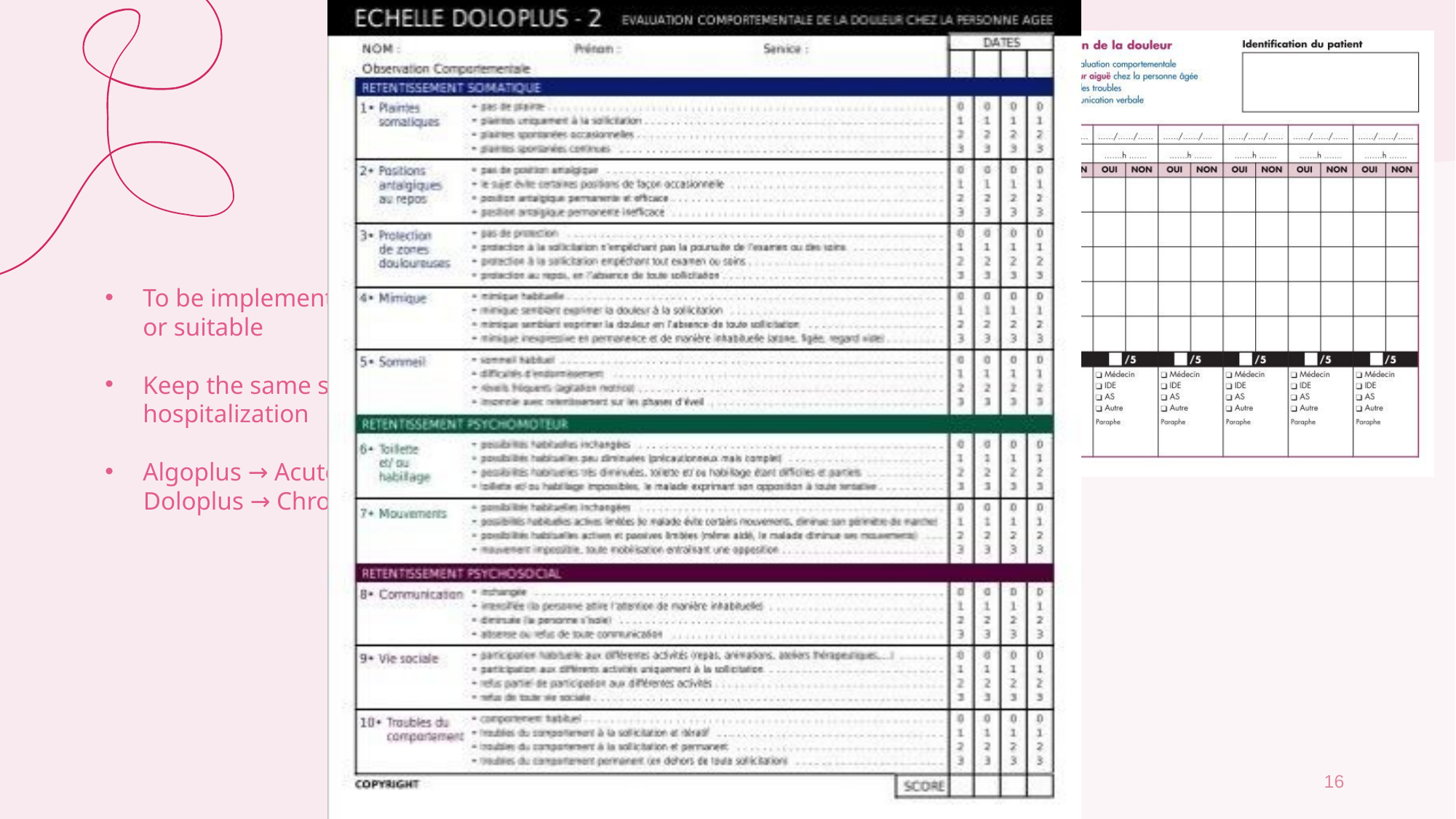

# Hetero evaluation
To be implemented if self-evaluation is not reliable or suitable
Keep the same scale until the end of the hospitalization
Algoplus → Acute pain
 Doloplus → Chronic pain
16

## Slide 17
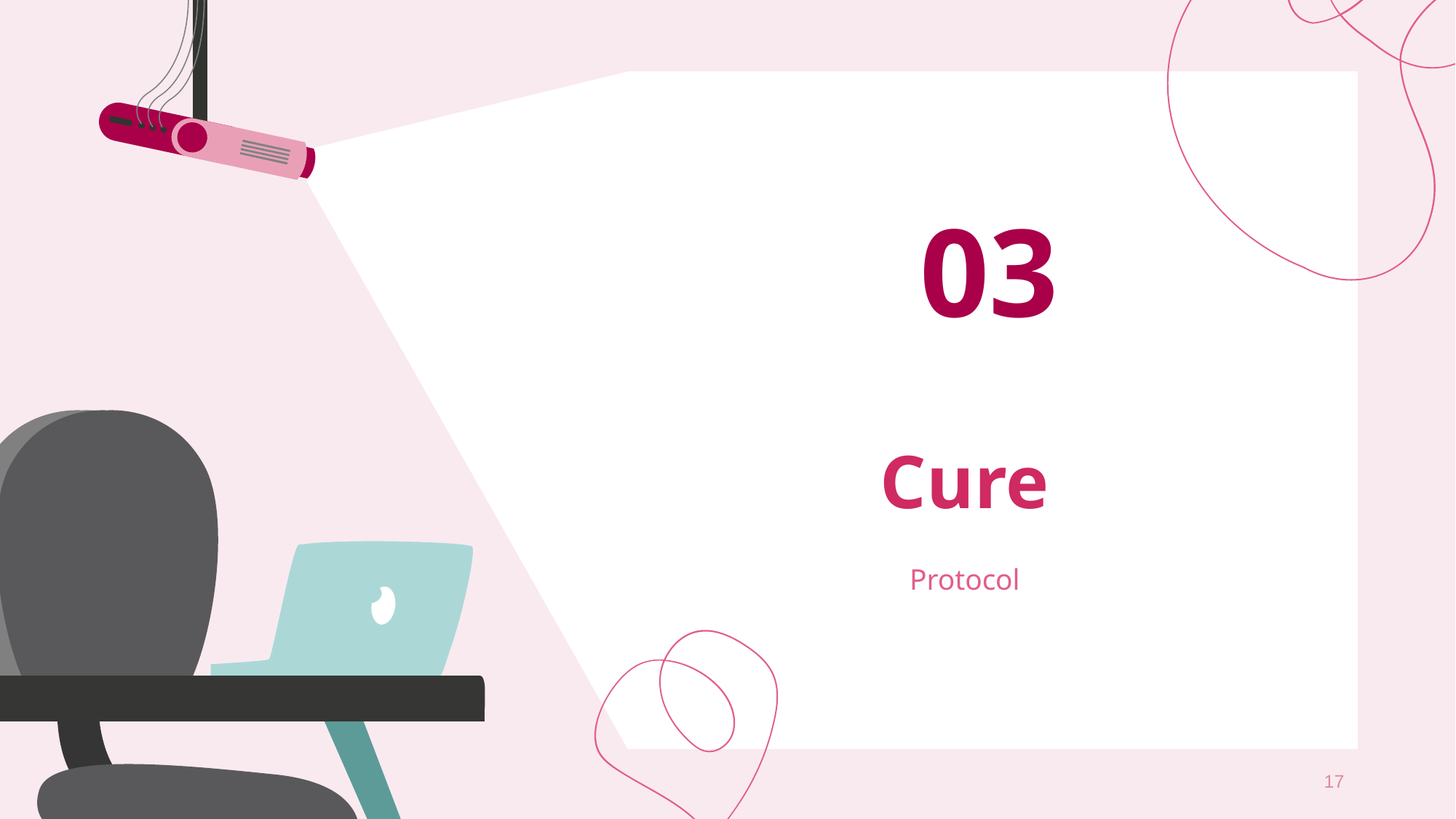

03
# Cure
Protocol
17

## Slide 18
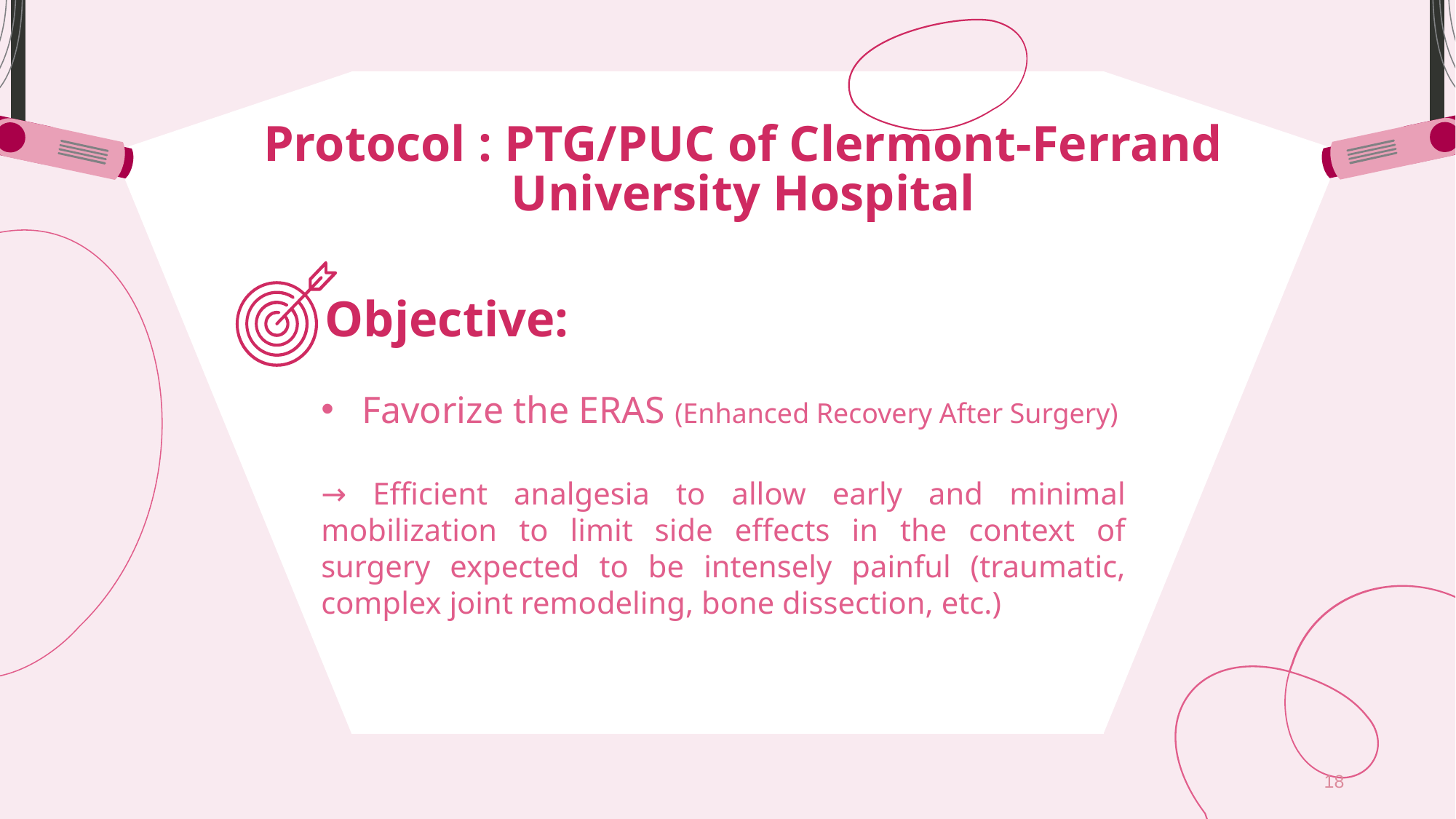

# Protocol : PTG/PUC of Clermont-Ferrand University Hospital
Objective:
Favorize the ERAS (Enhanced Recovery After Surgery)
→ Efficient analgesia to allow early and minimal mobilization to limit side effects in the context of surgery expected to be intensely painful (traumatic, complex joint remodeling, bone dissection, etc.)
18

## Slide 19
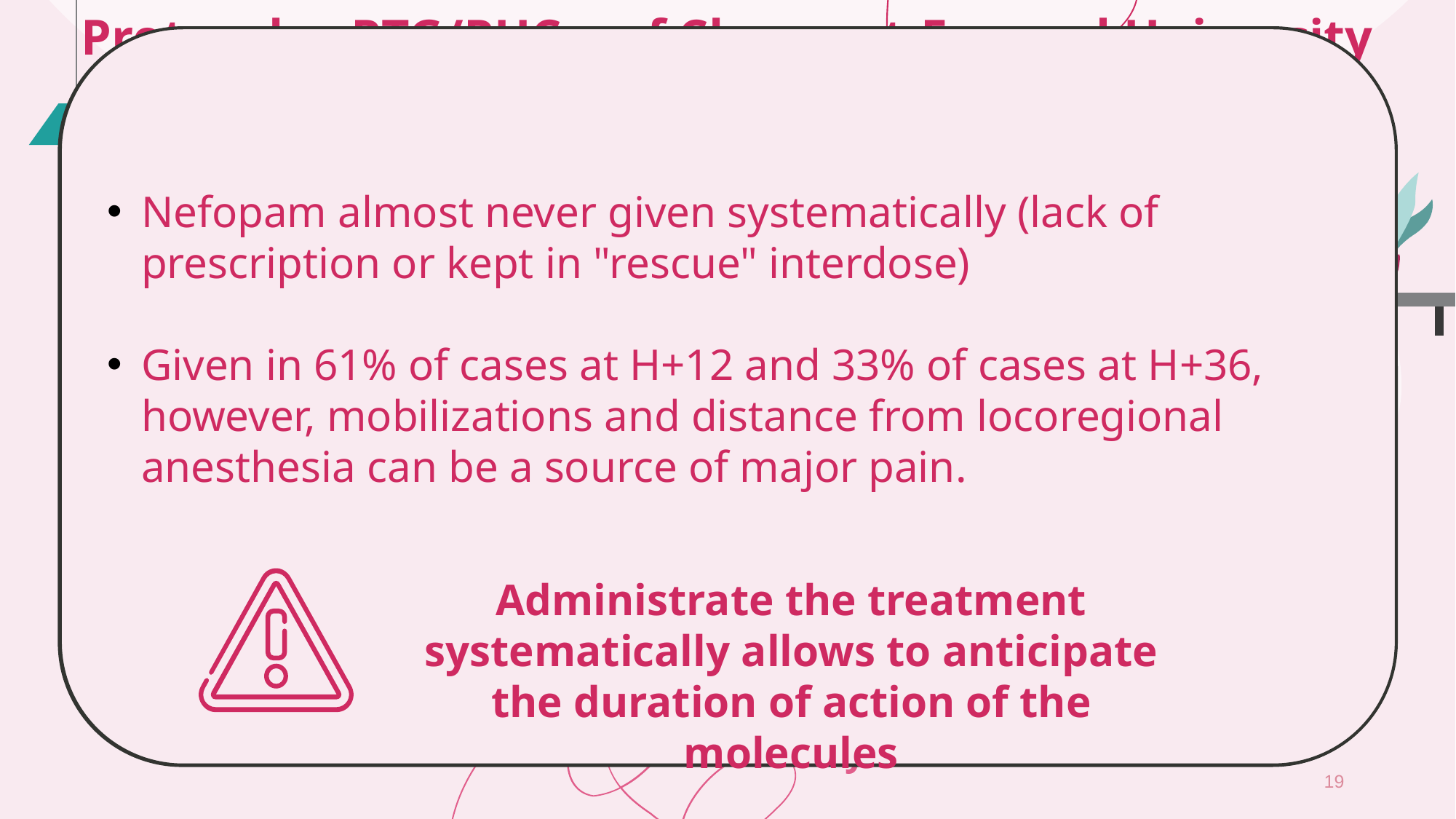

Protocol «  PTG/PUC » of Clermont-Ferrand University Hospital
Nefopam almost never given systematically (lack of prescription or kept in "rescue" interdose)
Given in 61% of cases at H+12 and 33% of cases at H+36, however, mobilizations and distance from locoregional anesthesia can be a source of major pain.
Paracetamol administrate almost all the time
Ketoprofene given to 2 patients on 3
Medical Treatment:
En systématique:
Paracetamol orally 1g * 4/day
Nefopam orally 20mg * 4/day
Ketoprofene orally 100 mg (prolonged release) * 2/day for 48 hours
Cryotherapy according to surgical prescription protocol
In need:
Actiskenan 5-10mg Immediate Release /4h if EN >3
Administrate the treatment systematically allows to anticipate the duration of action of the molecules
Treatment and dosage must be adapted to the patient's history
19

## Slide 20
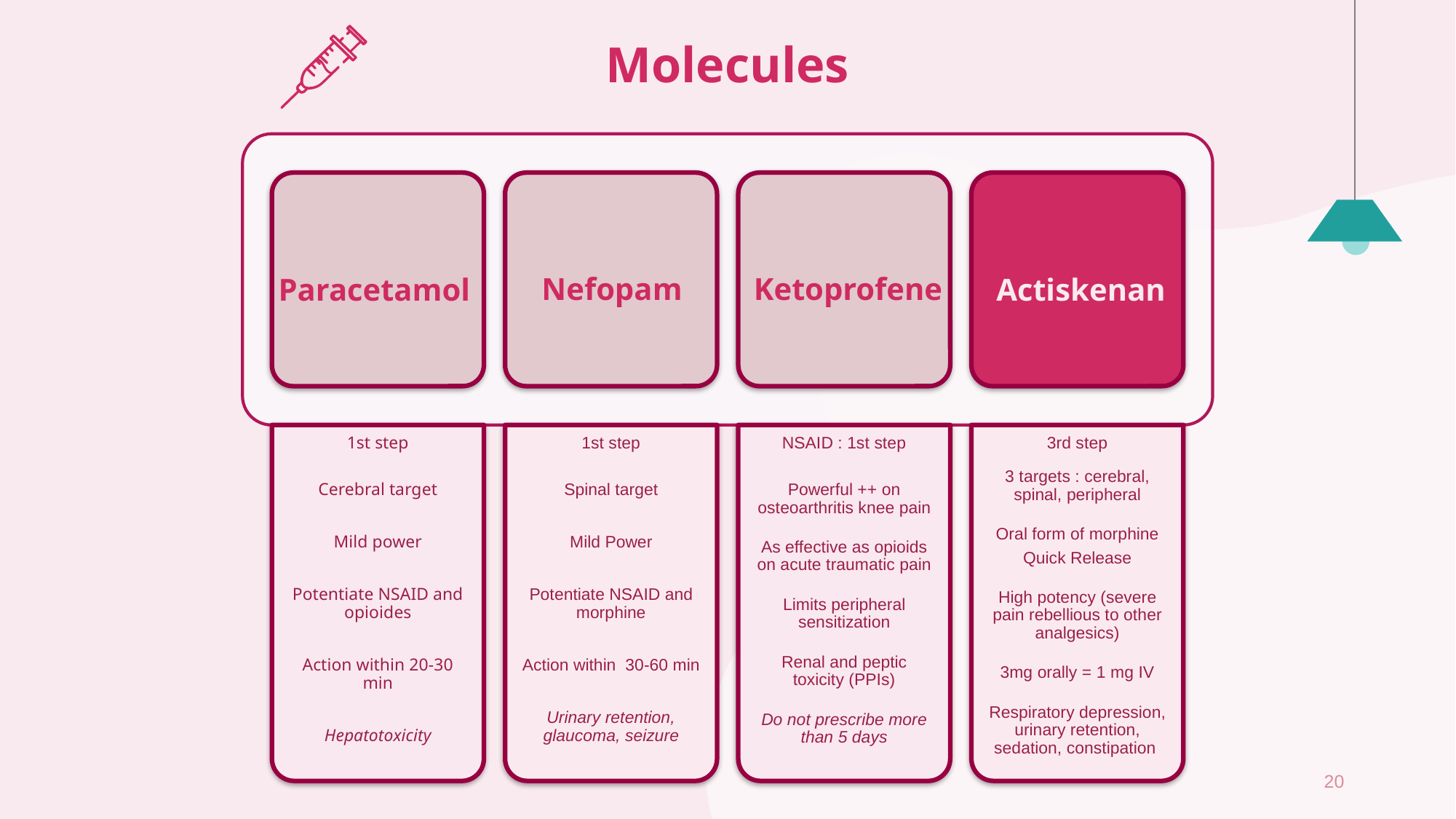

Molecules
1st step
Cerebral target
Mild power
Potentiate NSAID and opioides
Action within 20-30 min
Hepatotoxicity
1st step
Spinal target
Mild Power
Potentiate NSAID and morphine
Action within 30-60 min
Urinary retention, glaucoma, seizure
NSAID : 1st step
Powerful ++ on osteoarthritis knee pain
As effective as opioids on acute traumatic pain
Limits peripheral sensitization
Renal and peptic toxicity (PPIs)
Do not prescribe more than 5 days
3rd step
3 targets : cerebral, spinal, peripheral
Oral form of morphine
Quick Release
High potency (severe pain rebellious to other analgesics)
3mg orally = 1 mg IV
Respiratory depression, urinary retention, sedation, constipation
Nefopam
Ketoprofene
Paracetamol
Actiskenan
20

## Slide 21
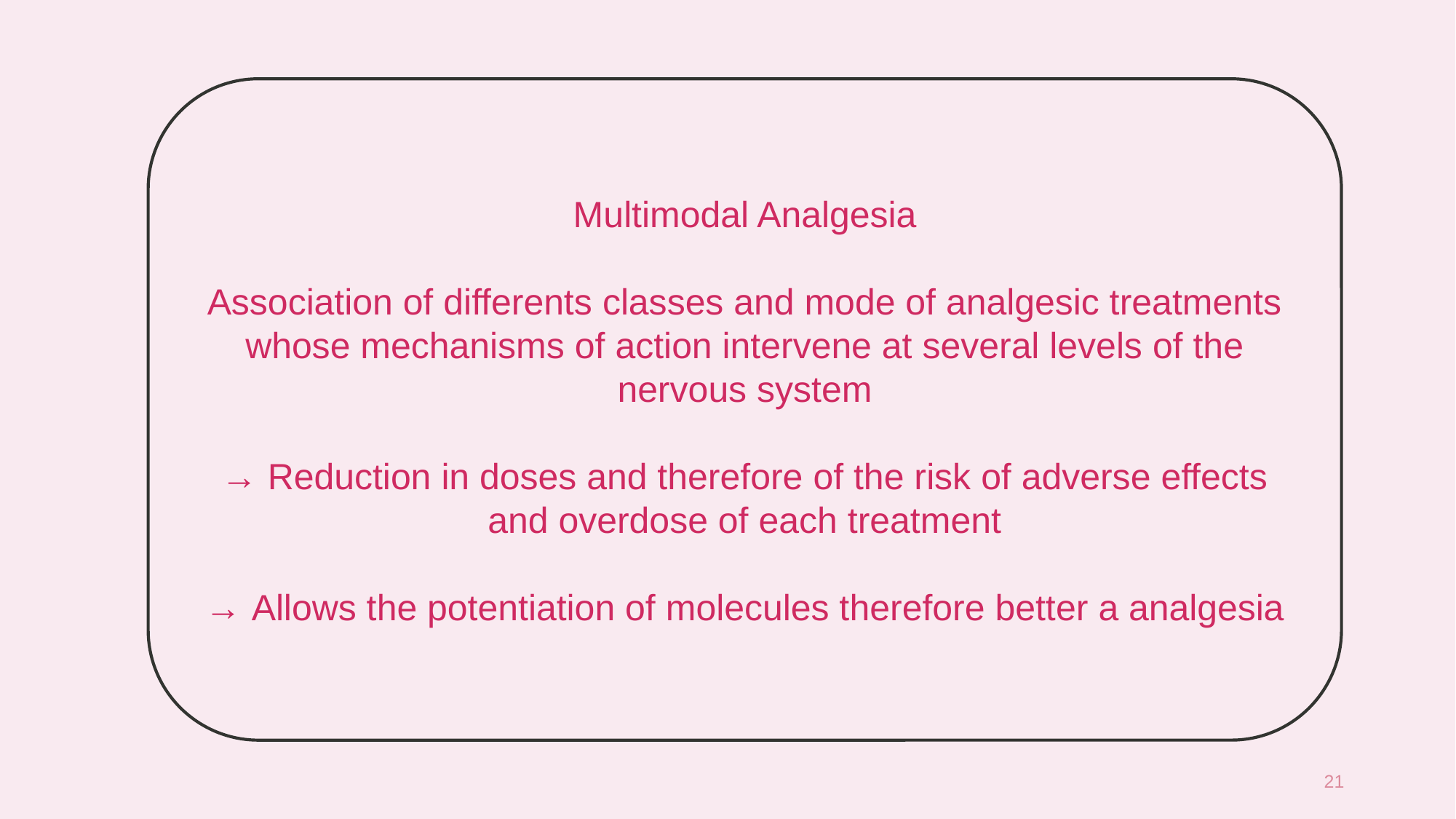

# WHO’s scale
Multimodal Analgesia
Association of differents classes and mode of analgesic treatments whose mechanisms of action intervene at several levels of the nervous system
→ Reduction in doses and therefore of the risk of adverse effects and overdose of each treatment
→ Allows the potentiation of molecules therefore better a analgesia
THIRD STEP
SECOND STEP
“Strong” Opioid Painkillers
Morphine
Fentanyl
Hydromorphone
Oxycodone
FIRST STEP
“Weak” Opioid Painkillers
Codeine
Dextropropoyphene
Tramadol
Non-Opioid Painkillers
Paracetamol
Aspirin
NSAID
MILD PAIN
MILD TO MODERATE PAIN
MODERATE TO SEVERE PAIN
1+ 2
Synergistic association
1+ 3
21

## Slide 22
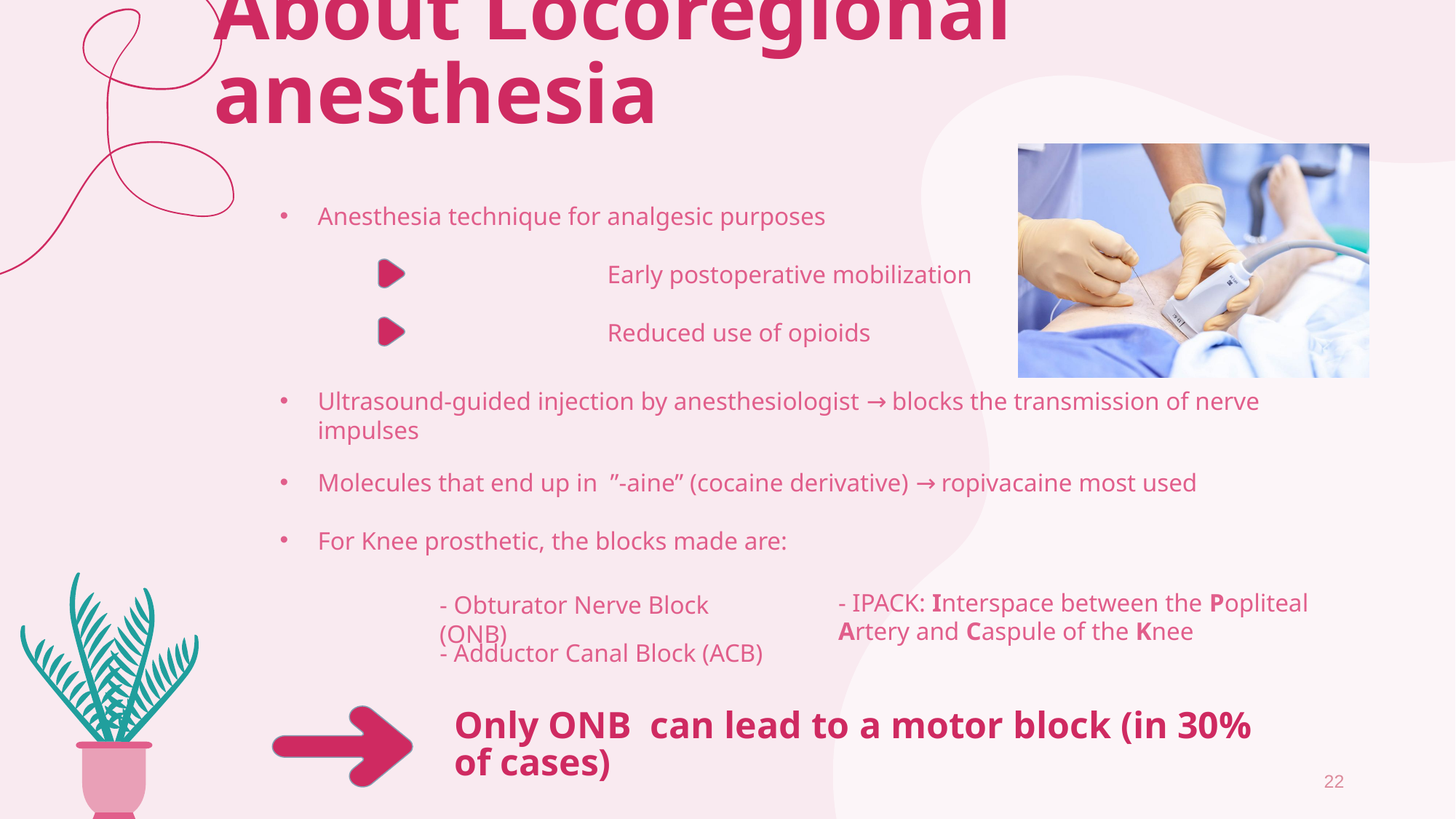

# About Locoregional anesthesia
Anesthesia technique for analgesic purposes
			Early postoperative mobilization
			Reduced use of opioids
Ultrasound-guided injection by anesthesiologist → blocks the transmission of nerve impulses
Molecules that end up in  ”-aine” (cocaine derivative) → ropivacaine most used
For Knee prosthetic, the blocks made are:
- IPACK: Interspace between the Popliteal Artery and Caspule of the Knee
- Obturator Nerve Block (ONB)
- Adductor Canal Block (ACB)
Only ONB can lead to a motor block (in 30% of cases)
22

## Slide 23
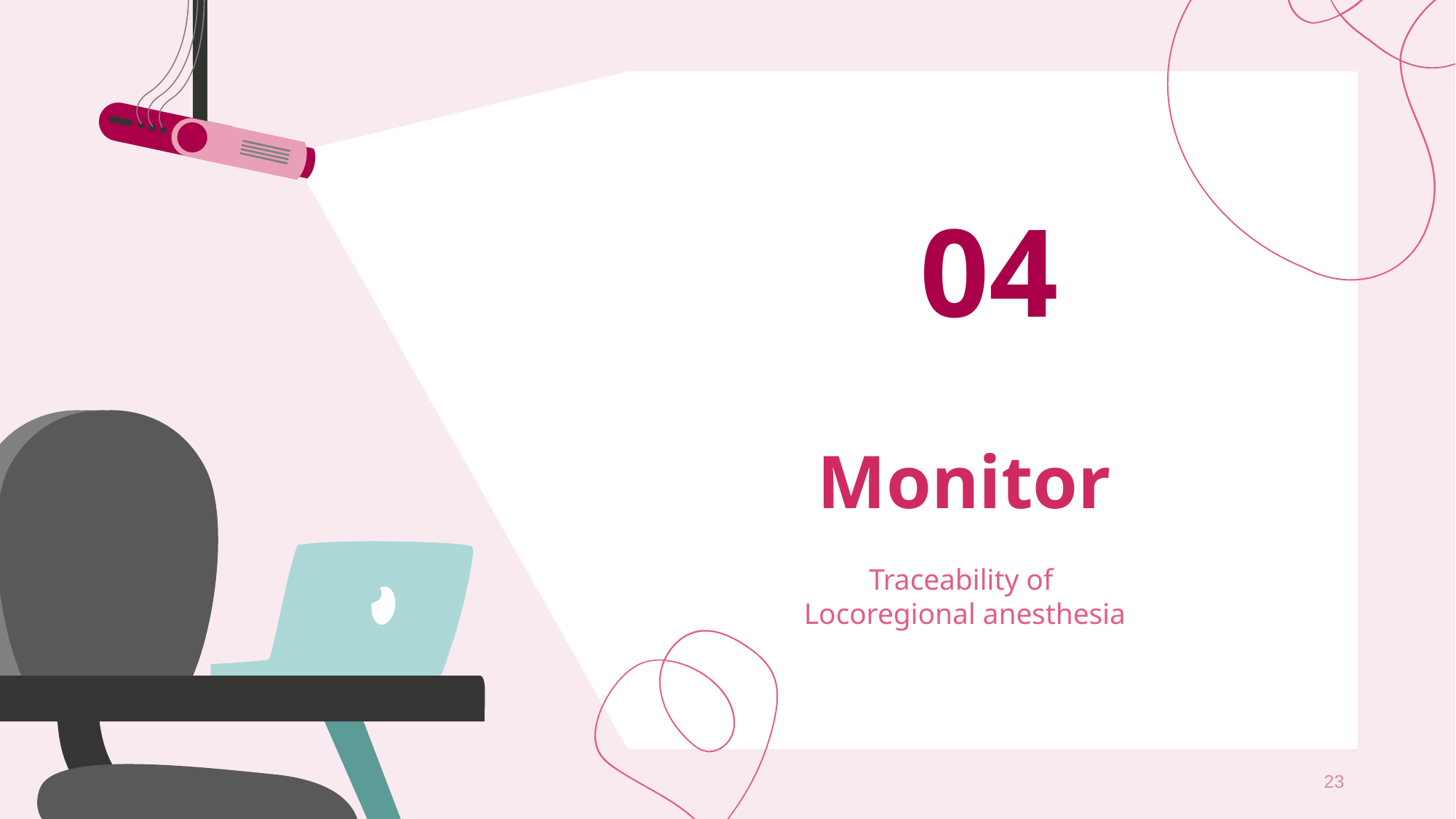

04
# Monitor
Traceability of
Locoregional anesthesia
23

## Slide 24
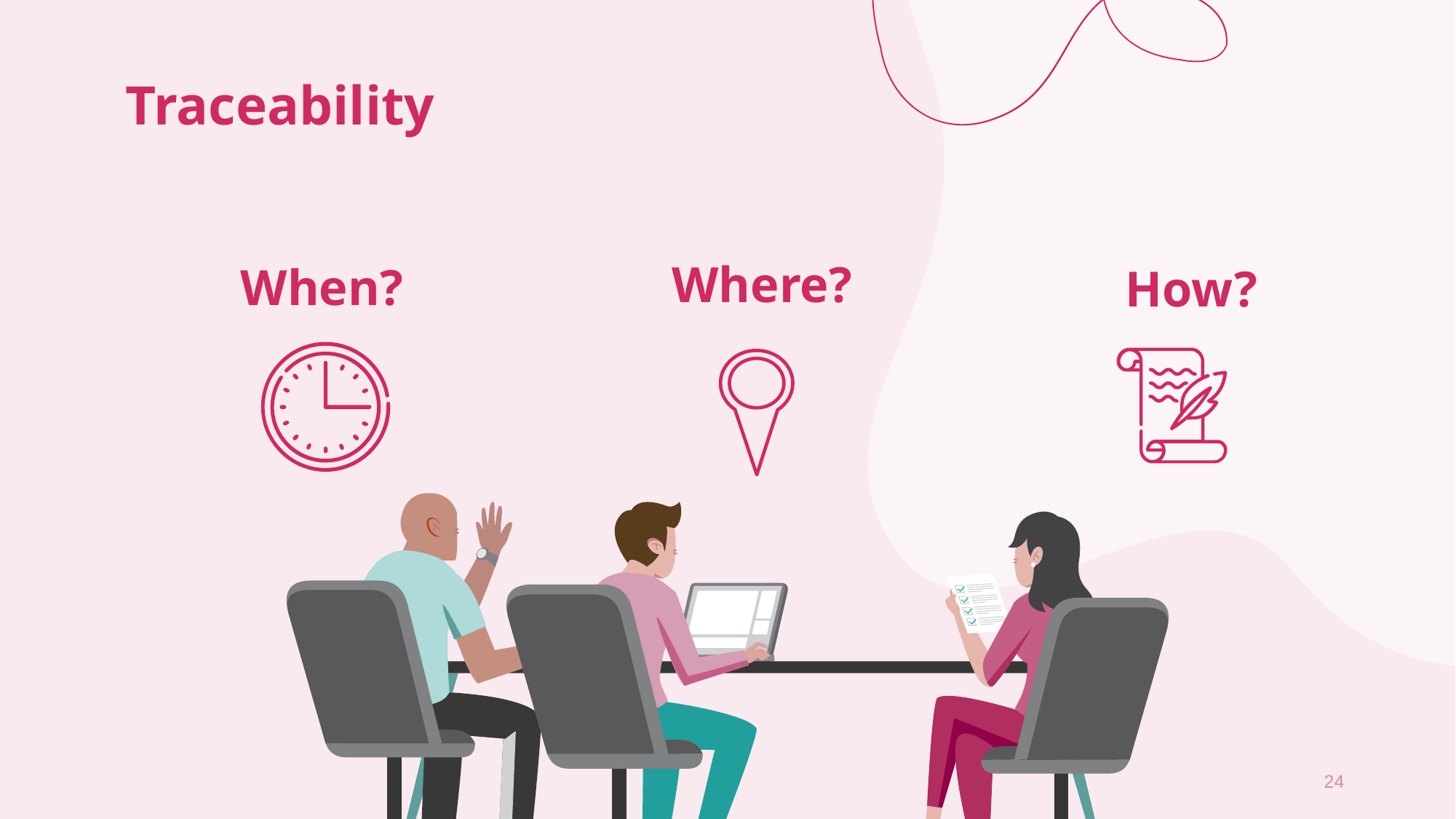

# Traceability
Where?
When?
How?
24

## Slide 25
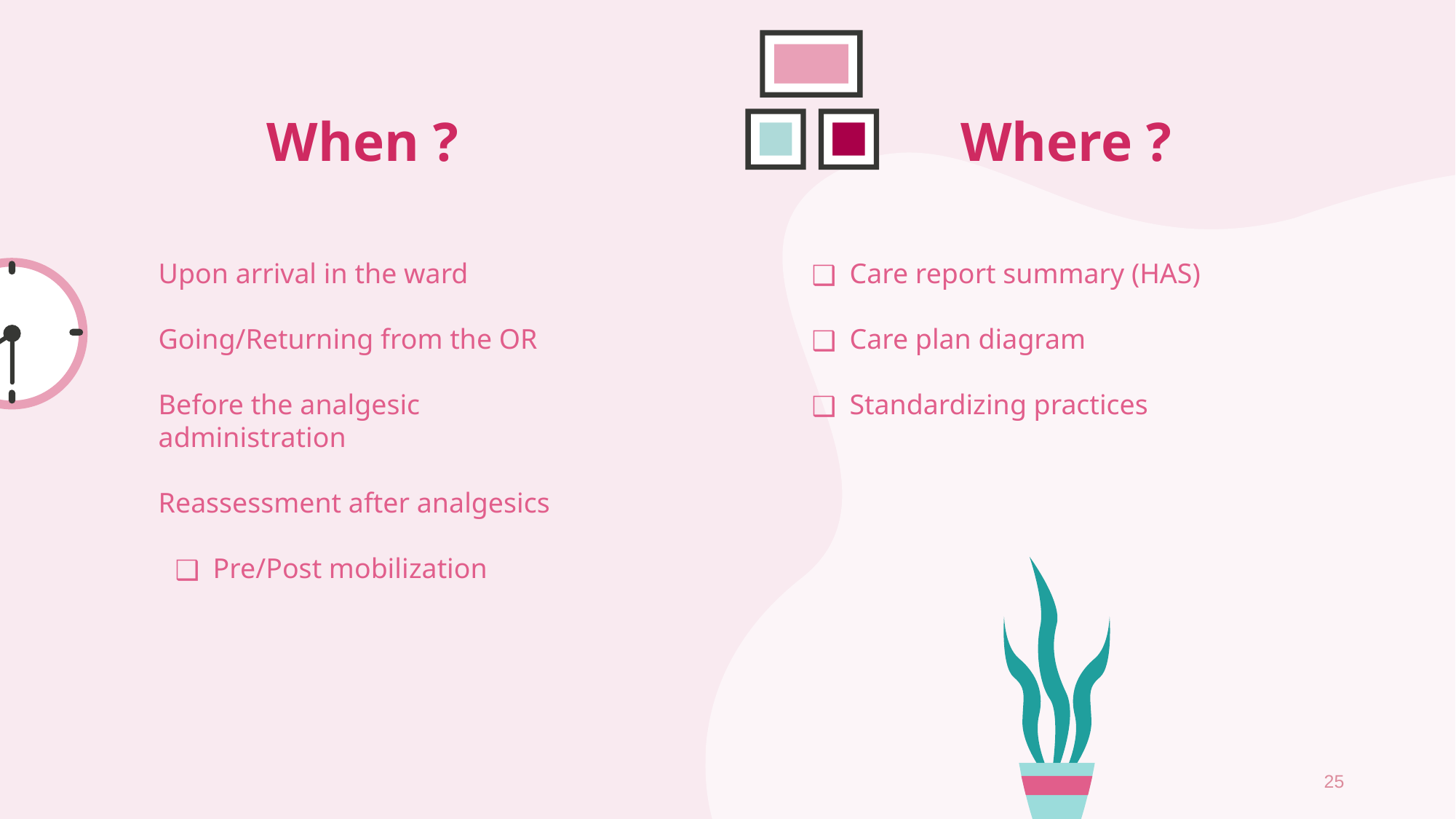

# When ?
Where ?
Upon arrival in the ward
Going/Returning from the OR
Before the analgesic administration
Reassessment after analgesics
Pre/Post mobilization
Care report summary (HAS)
Care plan diagram
Standardizing practices
25

## Slide 26
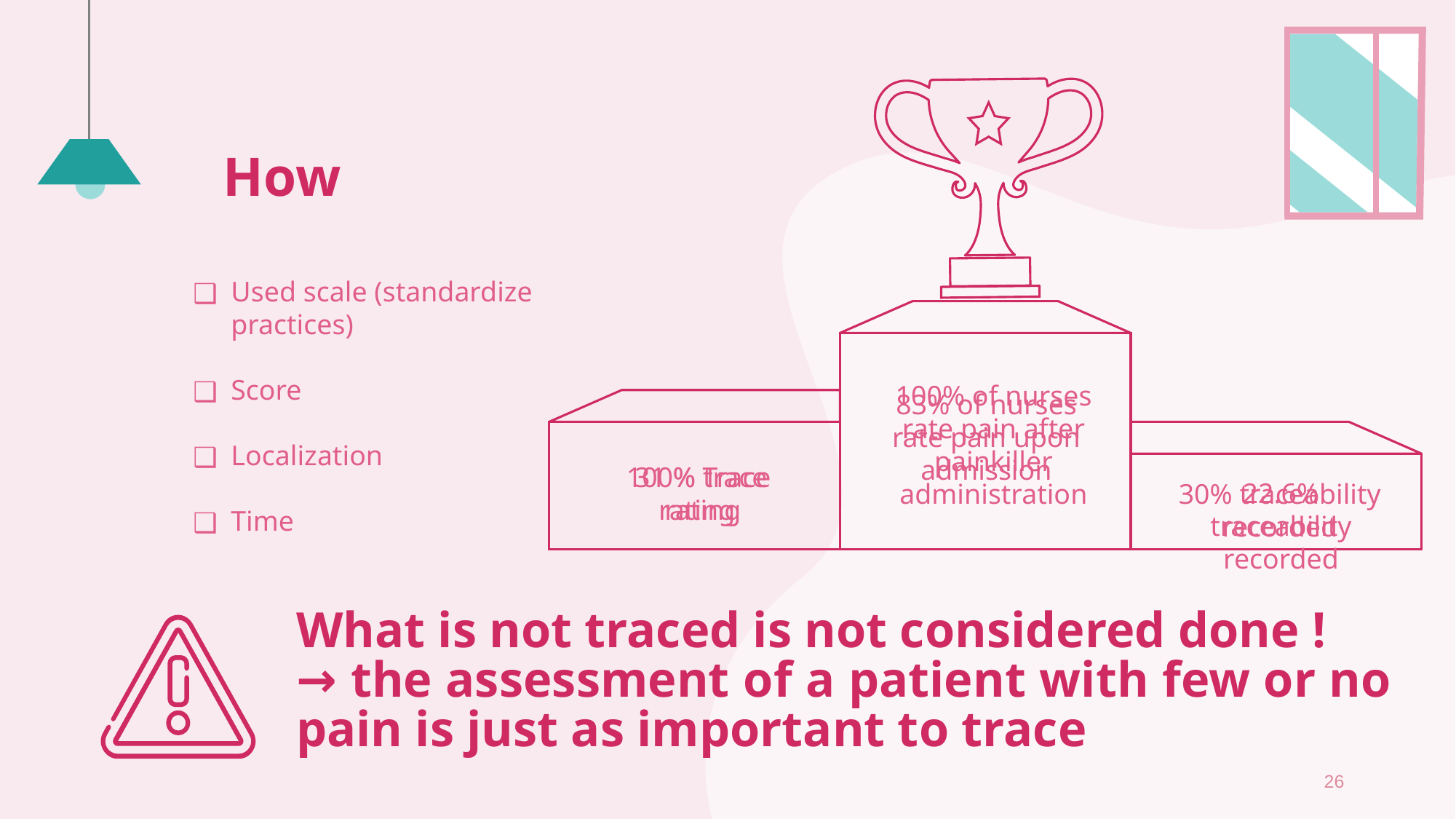

# How
Used scale (standardize practices)
Score
Localization
Time
100% of nurses rate pain after painkiller administration
83% of nurses rate pain upon admission
100% trace rating
31 % Trace rating
22.6% traceability recorded
30% traceability recorded
What is not traced is not considered done !
→ the assessment of a patient with few or no pain is just as important to trace
26

## Slide 27
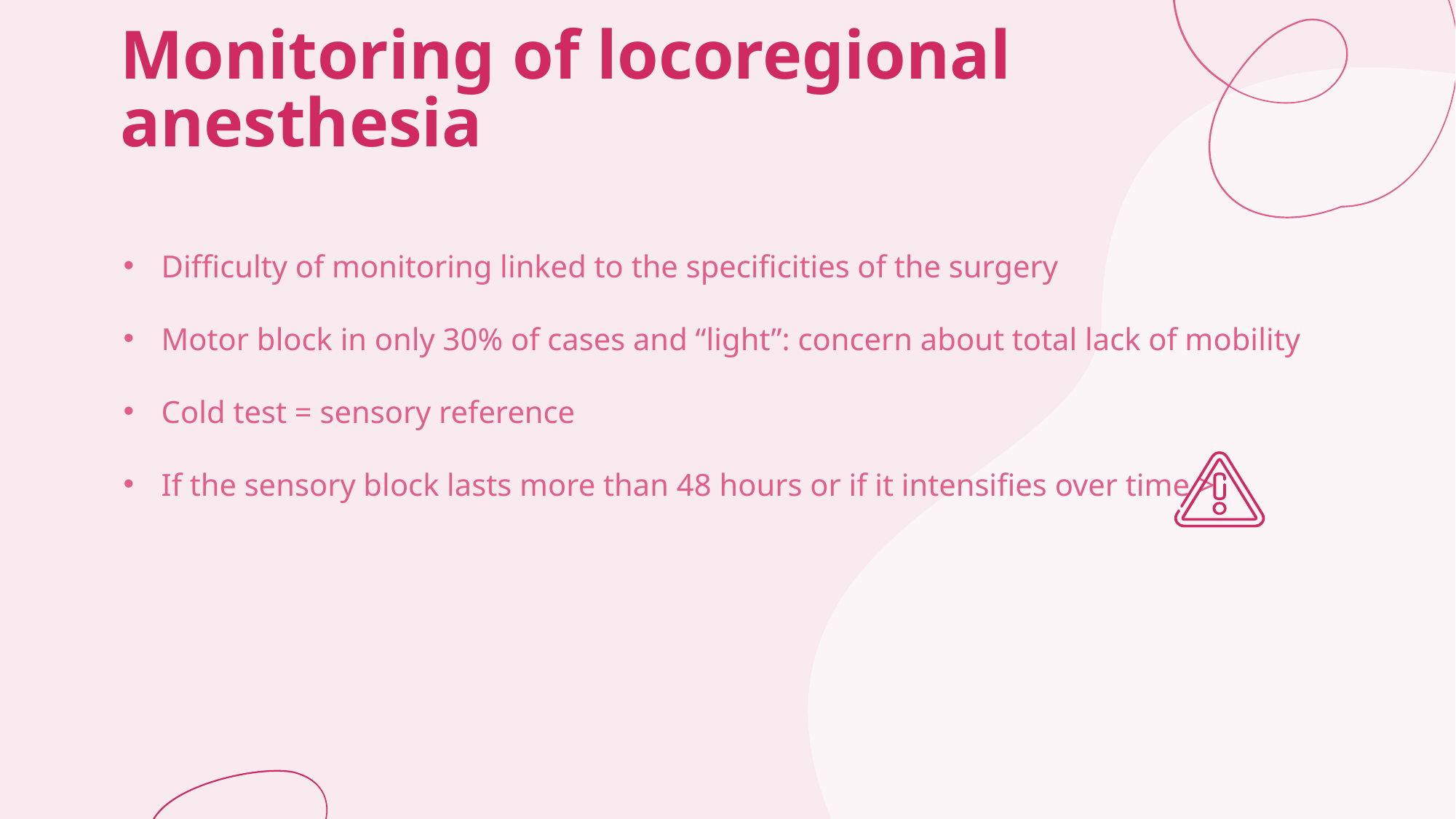

Monitoring of locoregional anesthesia
Difficulty of monitoring linked to the specificities of the surgery
Motor block in only 30% of cases and “light”: concern about total lack of mobility
Cold test = sensory reference
If the sensory block lasts more than 48 hours or if it intensifies over time >

## Slide 28
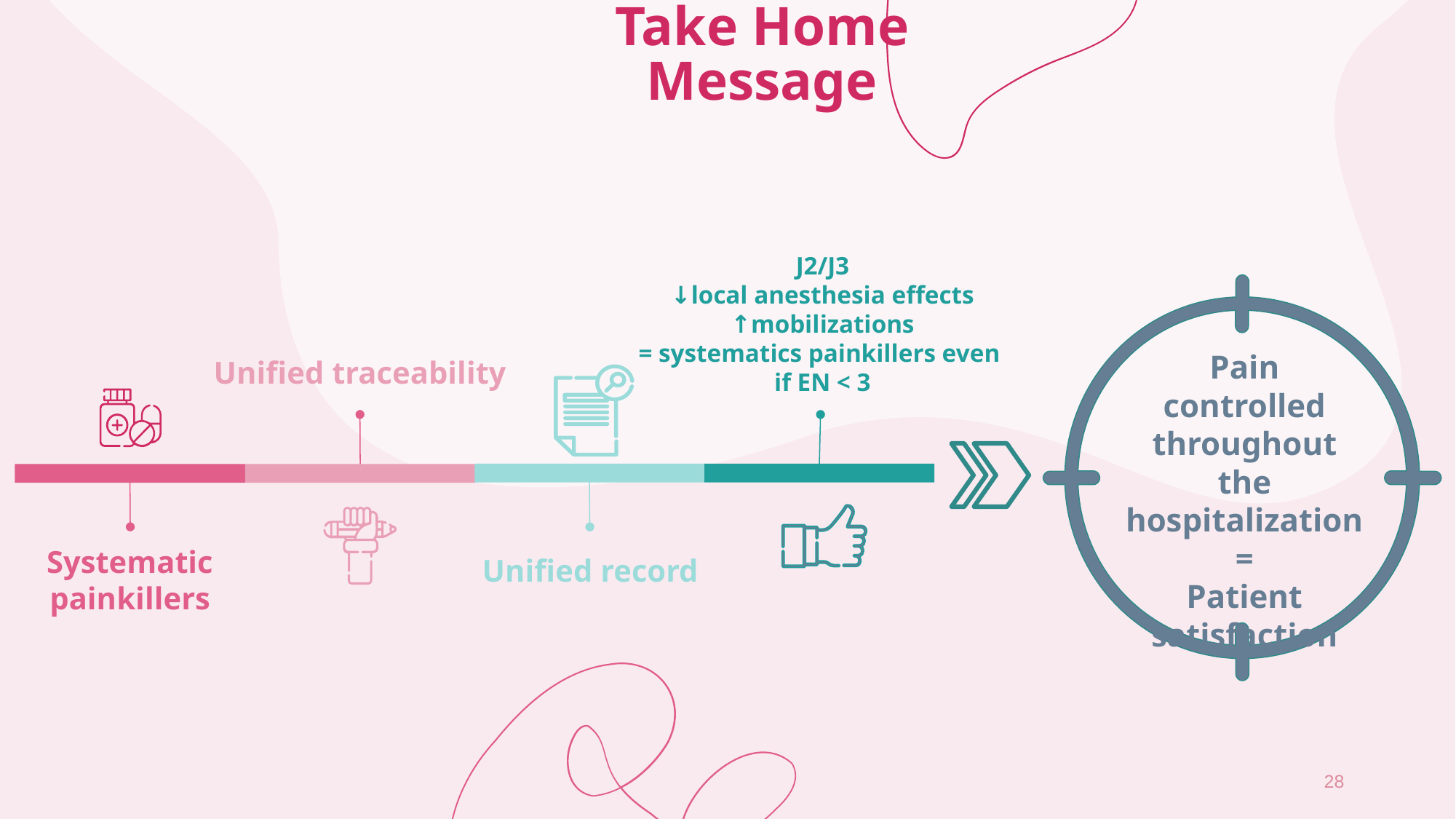

# Take Home Message
J2/J3
↓local anesthesia effects
↑mobilizations
= systematics painkillers even
if EN < 3
Unified traceability
Pain controlled throughout the hospitalization
=
Patient satisfaction
Systematic painkillers
Unified record
28

## Slide 29
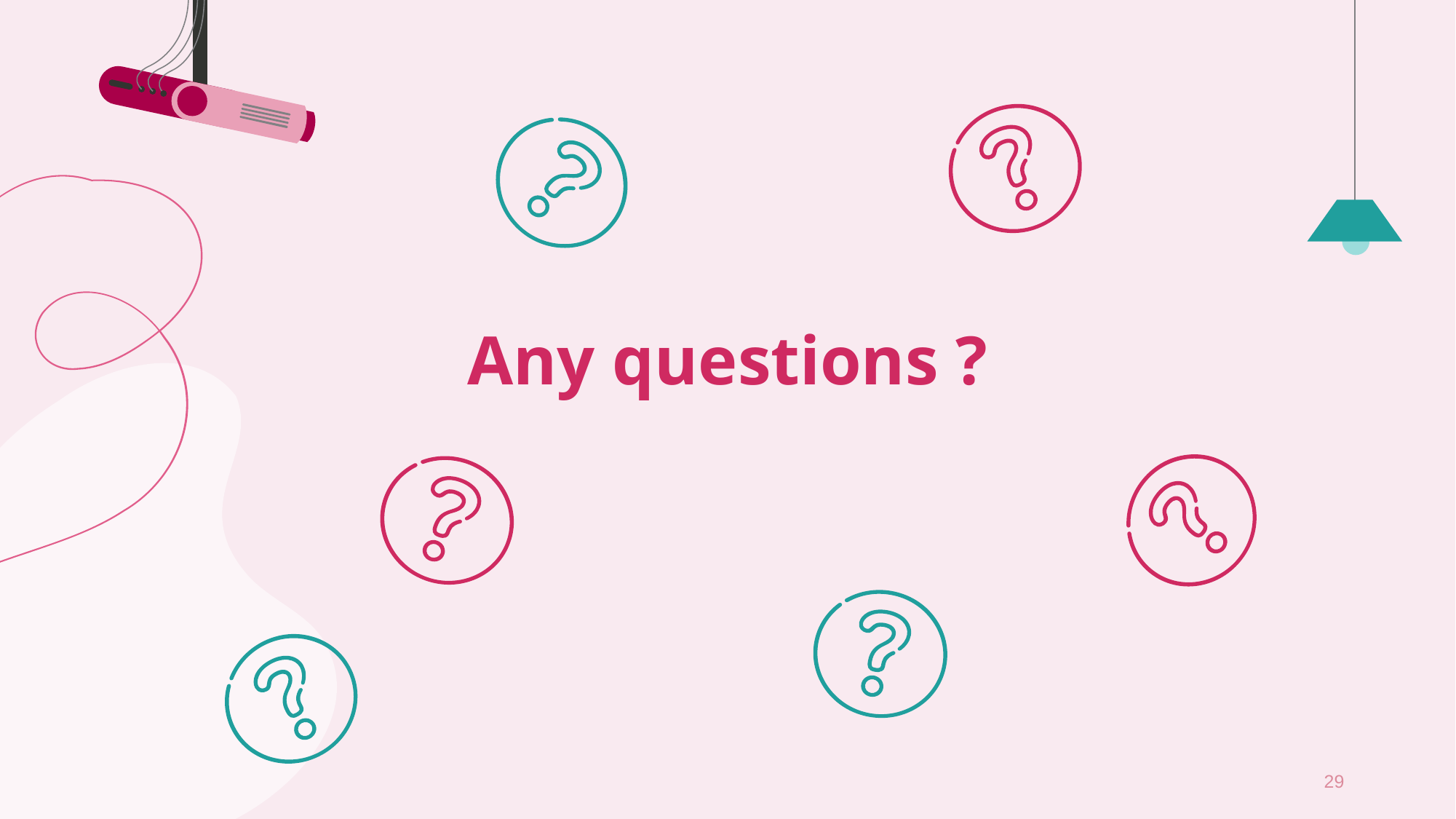

# Any questions ?
29

## Slide 30
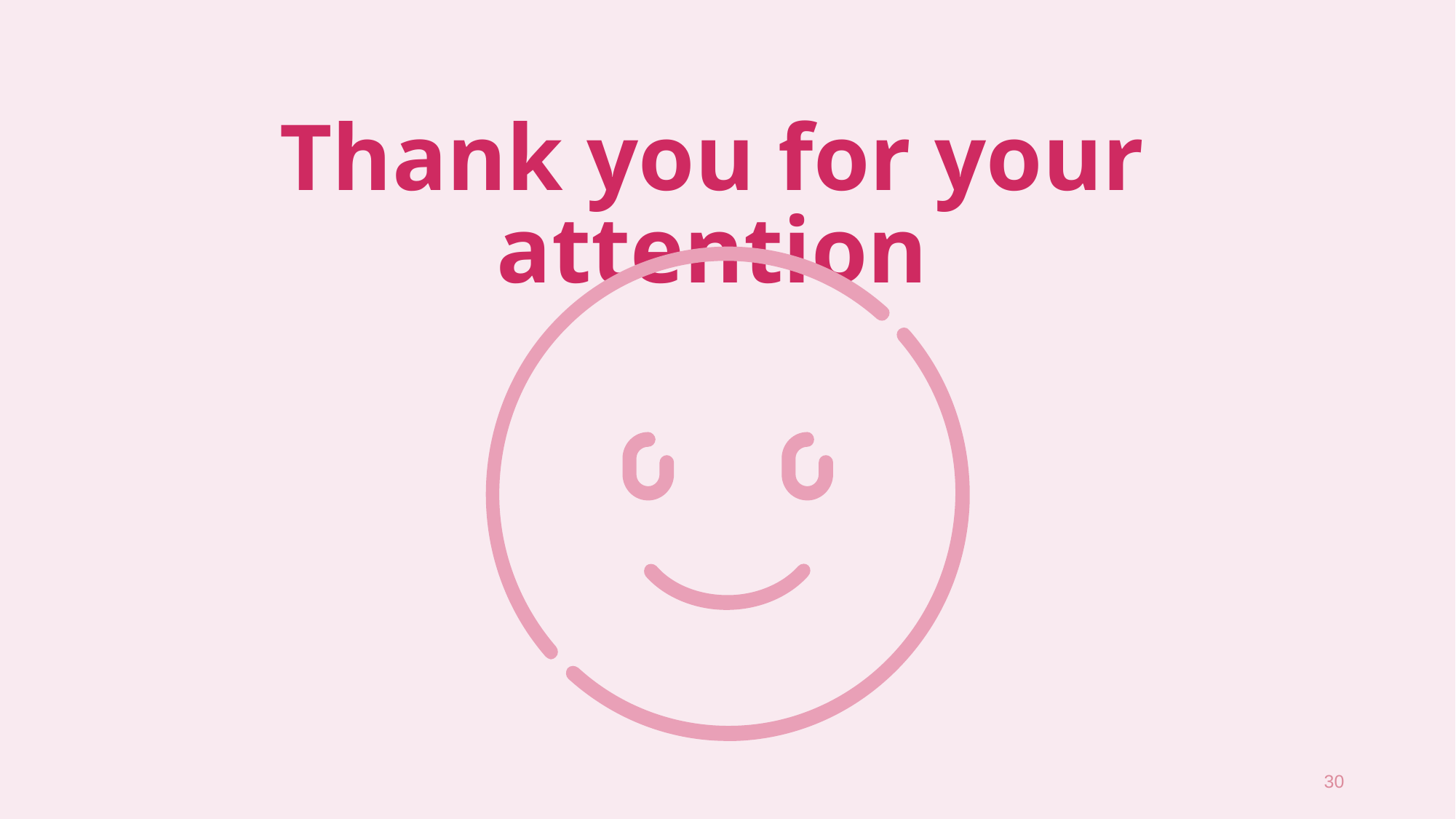

# Thank you for your attention
30

## Slide 31
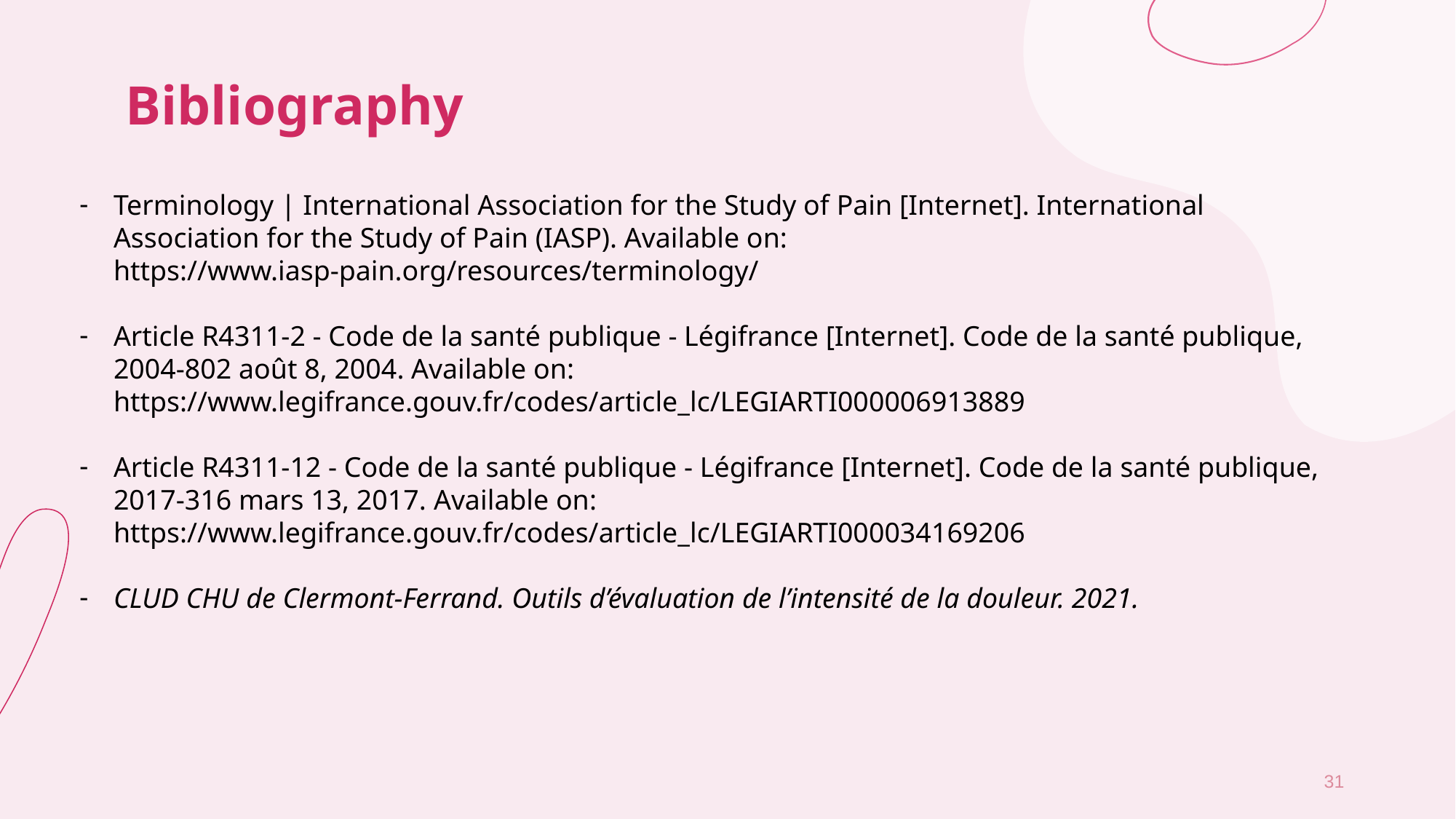

# Bibliography
Terminology | International Association for the Study of Pain [Internet]. International Association for the Study of Pain (IASP). Available on: https://www.iasp-pain.org/resources/terminology/
Article R4311-2 - Code de la santé publique - Légifrance [Internet]. Code de la santé publique, 2004‑802 août 8, 2004. Available on: https://www.legifrance.gouv.fr/codes/article_lc/LEGIARTI000006913889
Article R4311-12 - Code de la santé publique - Légifrance [Internet]. Code de la santé publique, 2017‑316 mars 13, 2017. Available on: https://www.legifrance.gouv.fr/codes/article_lc/LEGIARTI000034169206
CLUD CHU de Clermont-Ferrand. Outils d’évaluation de l’intensité de la douleur. 2021.
31
