# Supplementary material for: Impact of a nurse anesthetist student–led training program on perioperative pain management in total knee replacement: A prospective before and after study
Source: Int J Nurs Stud Adv. 2026 Jan 27;10:100495. doi: 10.1016/j.ijnsa.2026.100495 (PMC12925520; doi:10.1016/j.ijnsa.2026.100495)
Supplement: Supplementary file 1 [file mmc1.zip › Quick Formation pain - OR (english).pptx]

## Slide 1
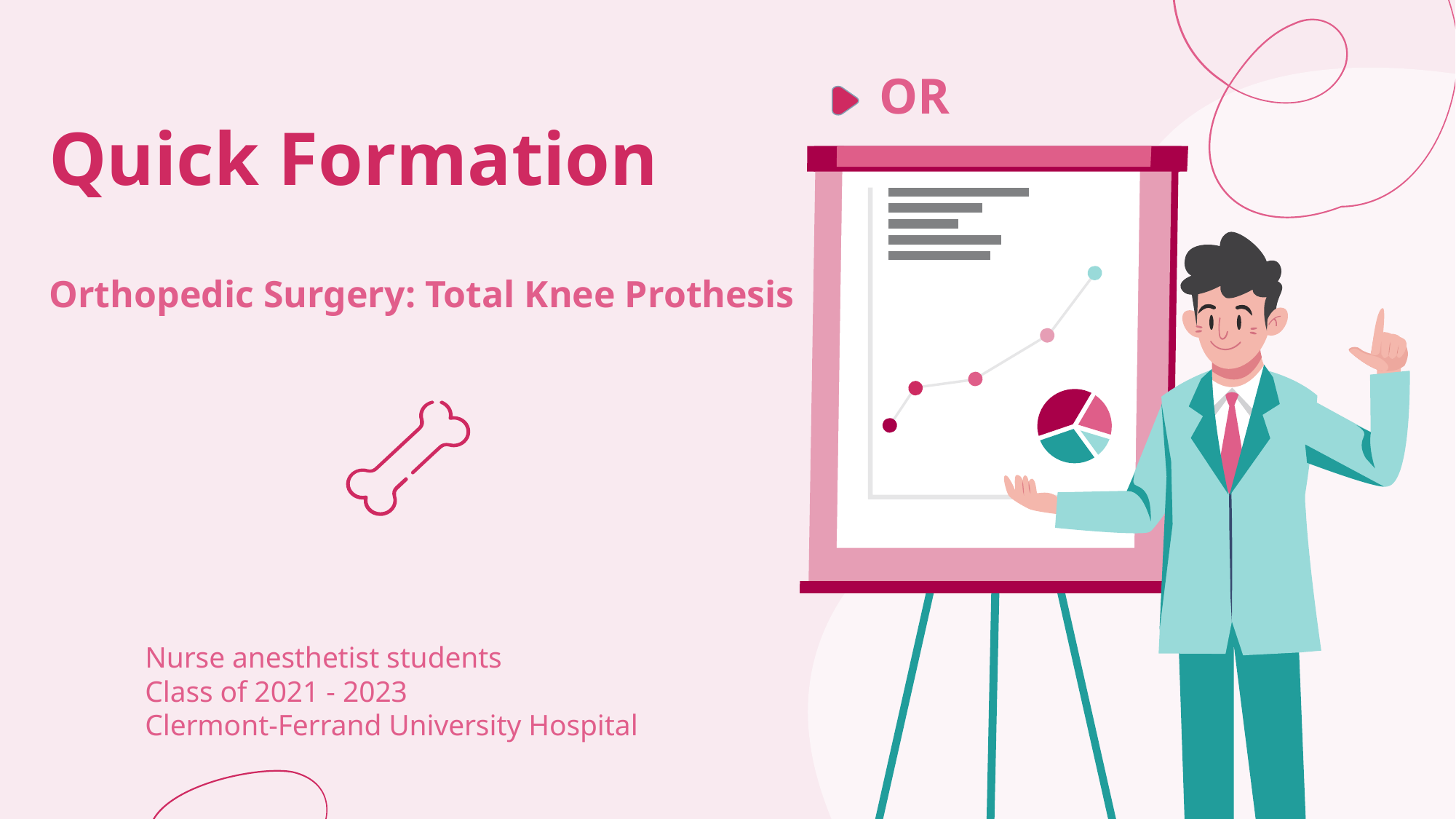

OR
# Quick Formation
Orthopedic Surgery: Total Knee Prothesis
Nurse anesthetist students
Class of 2021 - 2023
Clermont-Ferrand University Hospital

## Slide 2
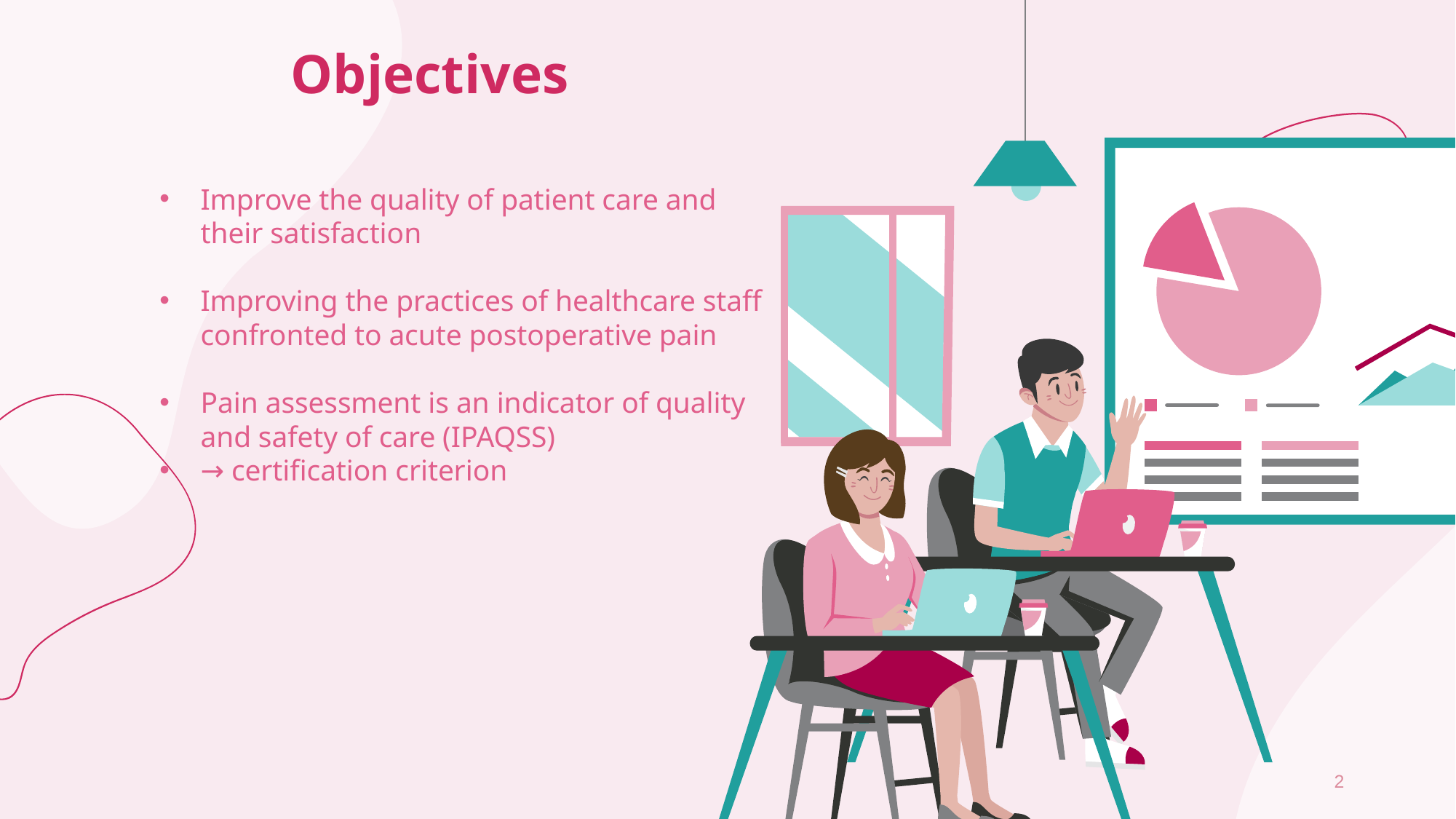

# Objectives
Improve the quality of patient care and their satisfaction
Improving the practices of healthcare staff confronted to acute postoperative pain
Pain assessment is an indicator of quality and safety of care (IPAQSS)
→ certification criterion
2

## Slide 3
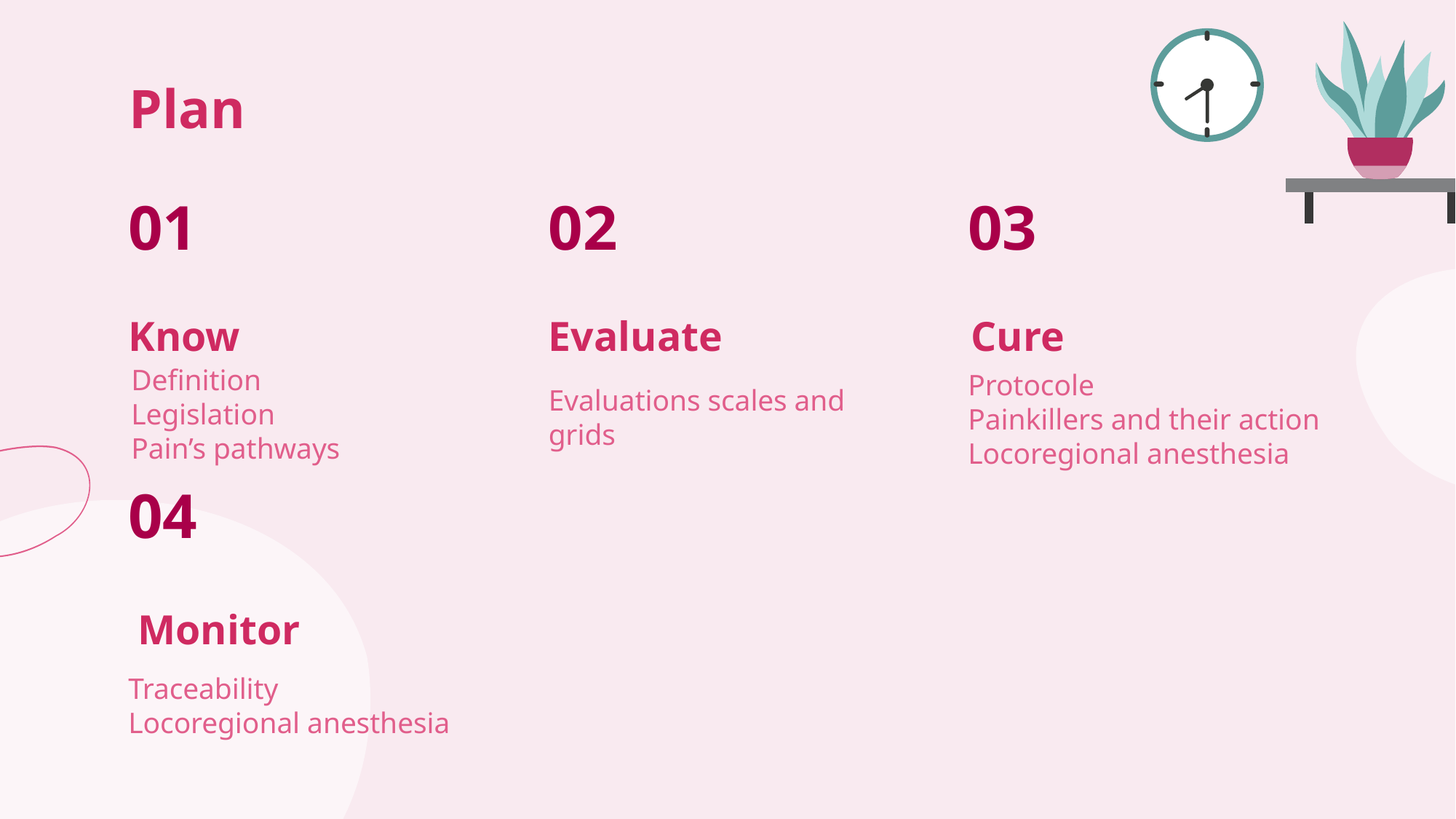

Plan
# 01
02
03
Know
Evaluate
Cure
Definition
Legislation
Pain’s pathways
Protocole
Painkillers and their action
Locoregional anesthesia
Evaluations scales and grids
04
Monitor
Traceability
Locoregional anesthesia

## Slide 4
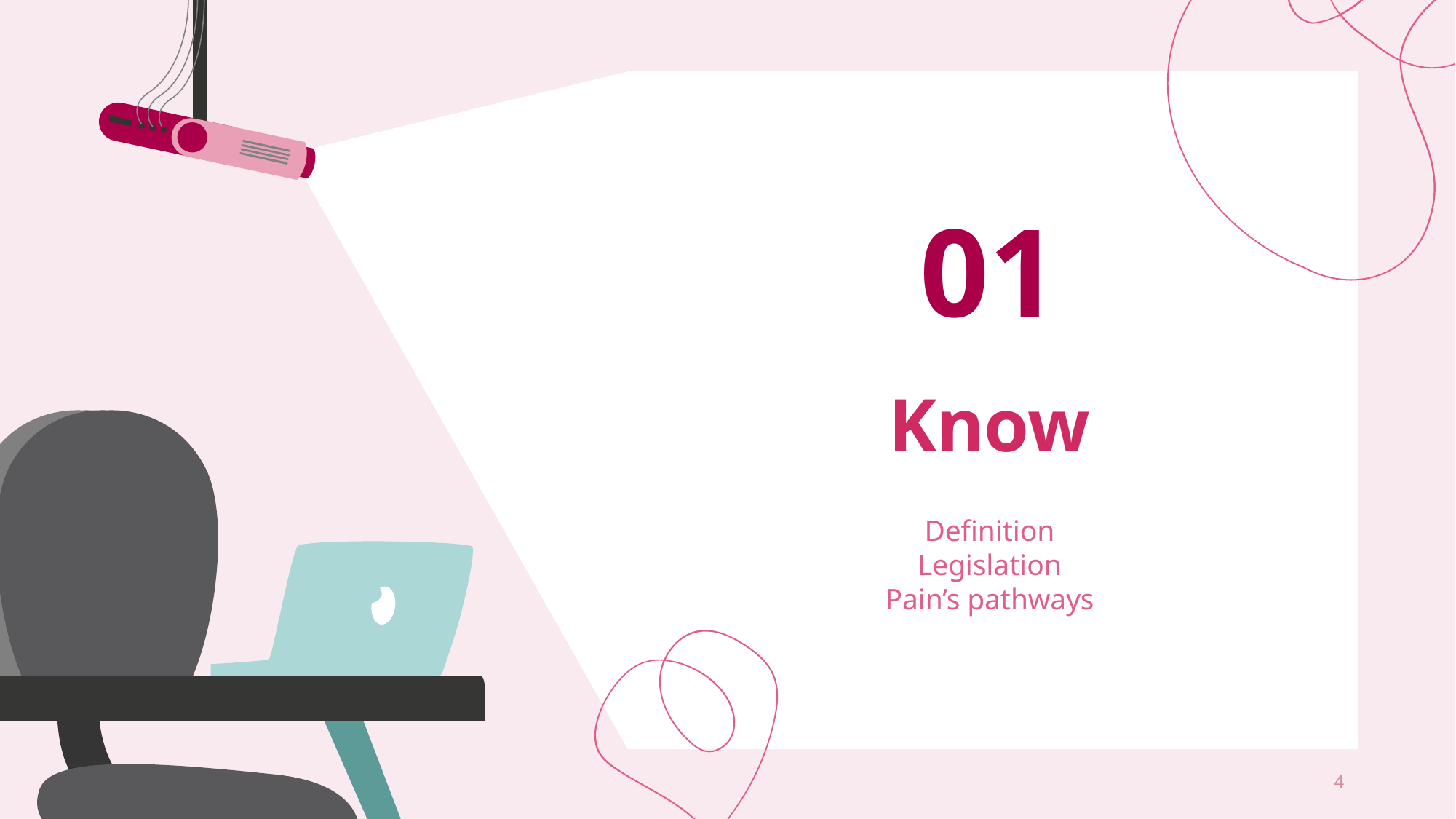

01
# Know
Definition
Legislation
Pain’s pathways
4

## Slide 5
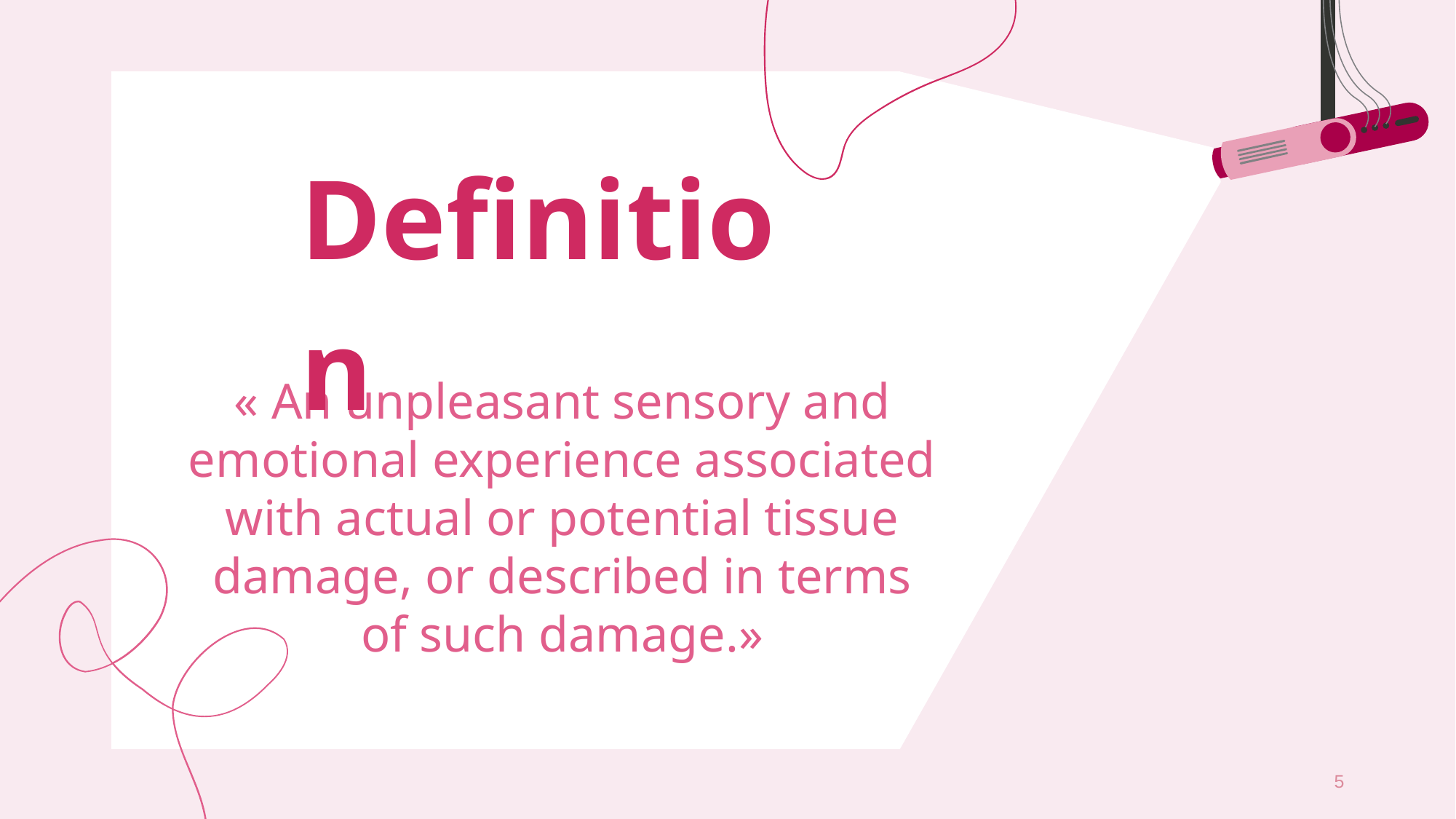

Definition
# « An unpleasant sensory and emotional experience associated with actual or potential tissue damage, or described in terms of such damage.»
5

## Slide 6
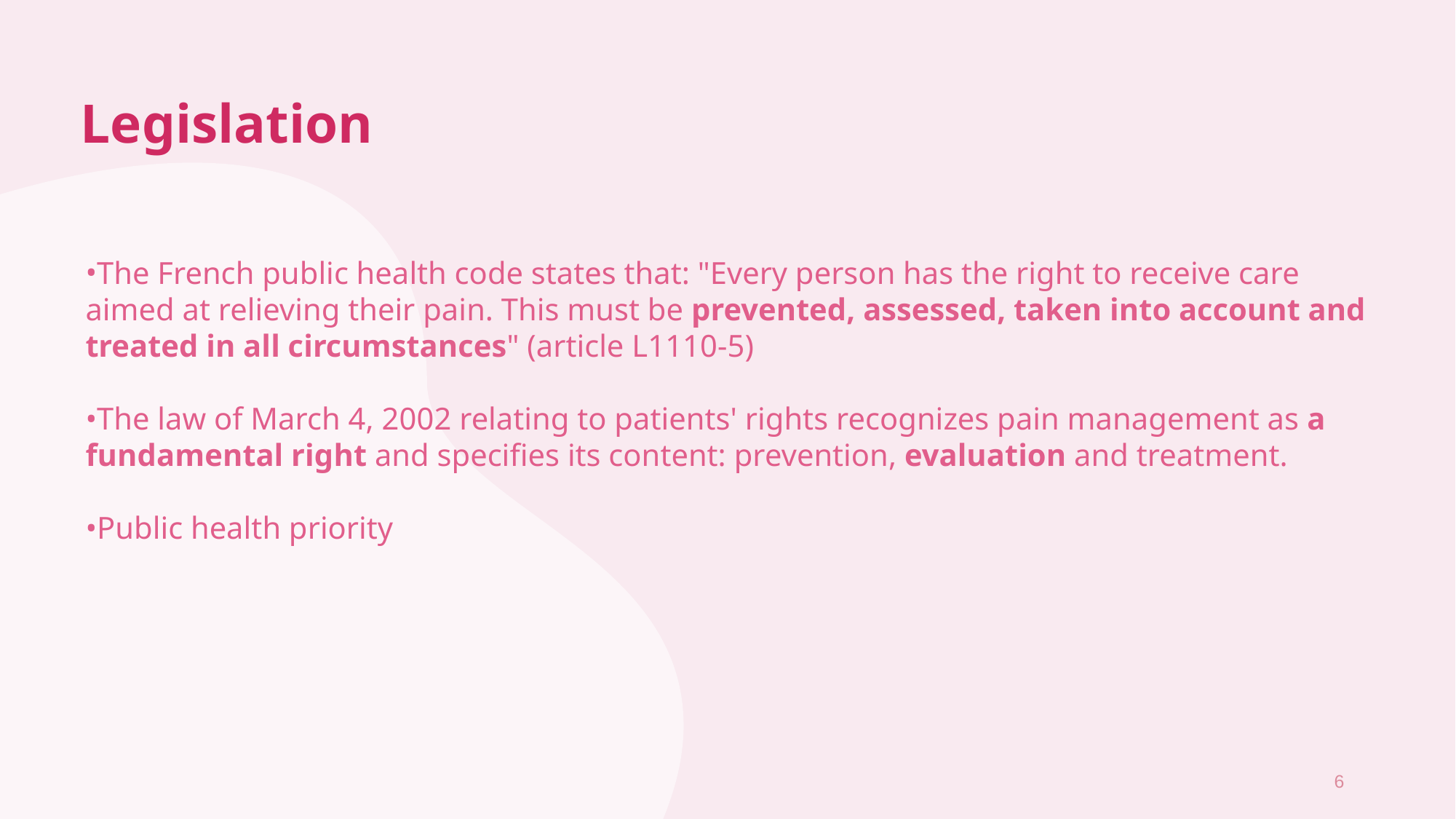

# Legislation
The French public health code states that: "Every person has the right to receive care aimed at relieving their pain. This must be prevented, assessed, taken into account and treated in all circumstances" (article L1110-5)
The law of March 4, 2002 relating to patients' rights recognizes pain management as a fundamental right and specifies its content: prevention, evaluation and treatment.
Public health priority
6

## Slide 7
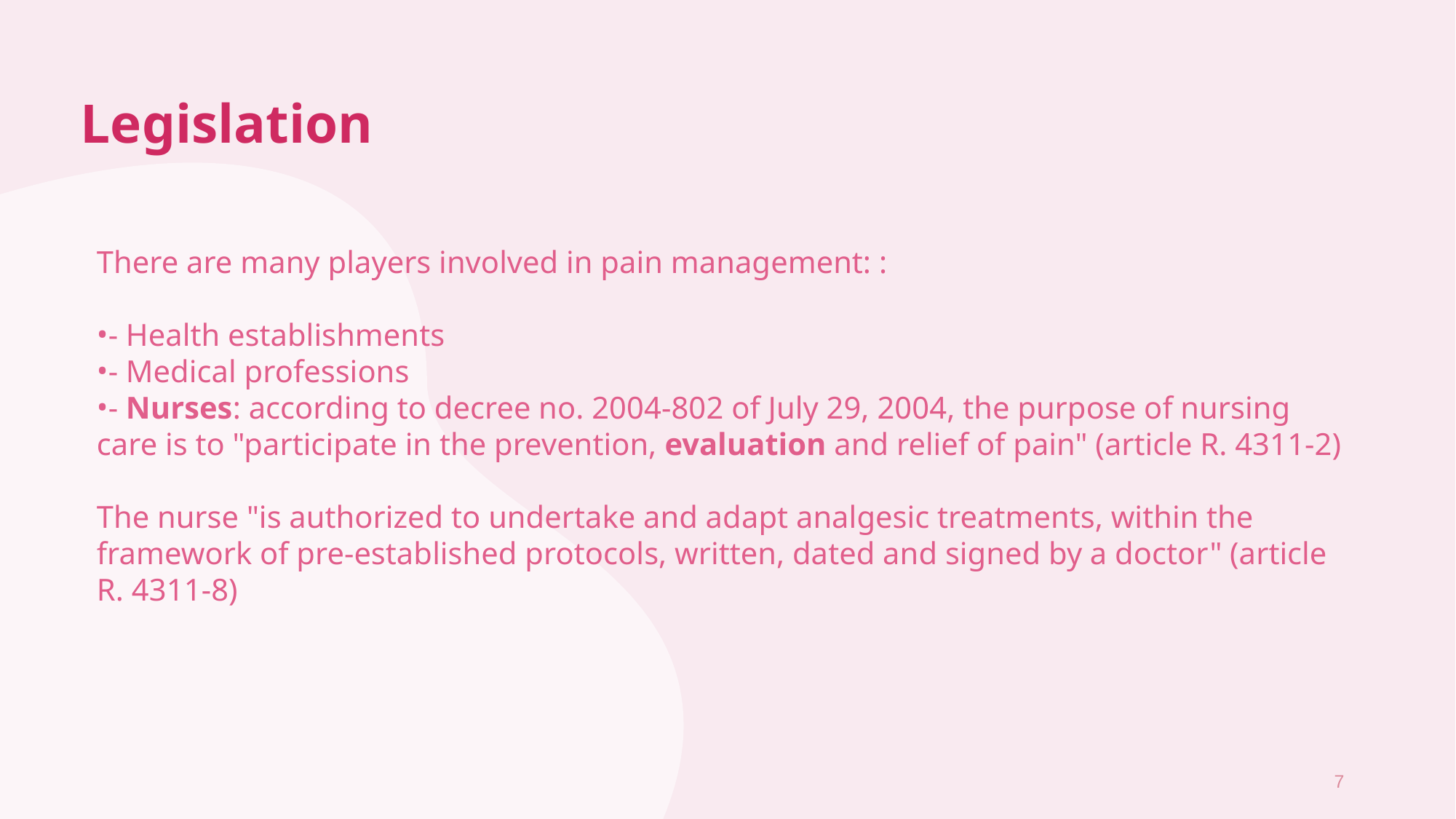

# Legislation
There are many players involved in pain management: :
- Health establishments
- Medical professions
- Nurses: according to decree no. 2004-802 of July 29, 2004, the purpose of nursing care is to "participate in the prevention, evaluation and relief of pain" (article R. 4311-2)
The nurse "is authorized to undertake and adapt analgesic treatments, within the framework of pre-established protocols, written, dated and signed by a doctor" (article R. 4311-8)
7

## Slide 8
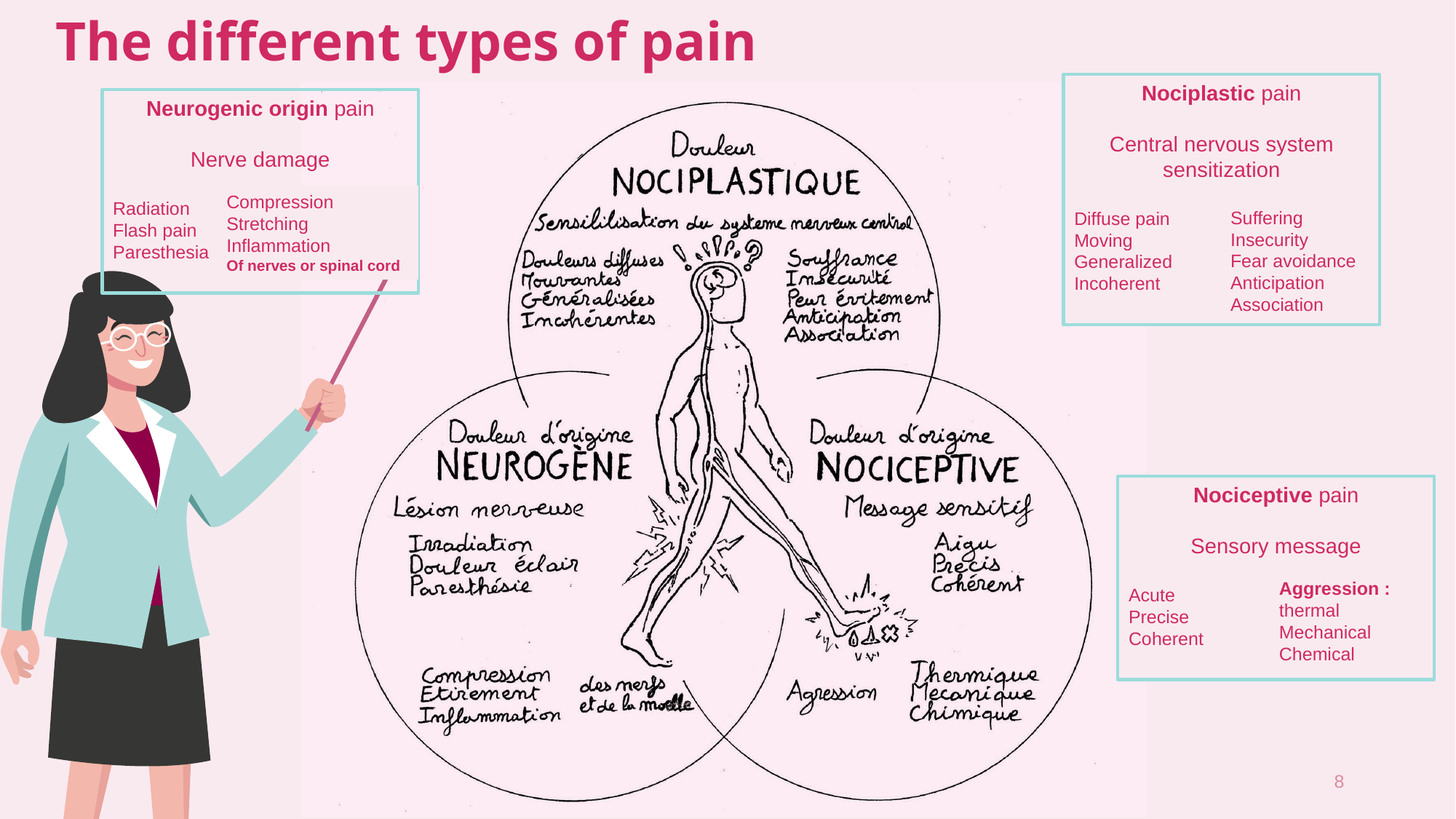

# The different types of pain
Nociplastic pain
Central nervous system sensitization
Diffuse pain
Moving
Generalized
Incoherent
Suffering
Insecurity
Fear avoidance
Anticipation
Association
Neurogenic origin pain
Nerve damage
Radiation
Flash pain
Paresthesia
Compression
Stretching
Inflammation
Of nerves or spinal cord
Nociceptive pain
Sensory message
Acute
Precise
Coherent
Aggression :
thermal
Mechanical
Chemical
8

## Slide 9
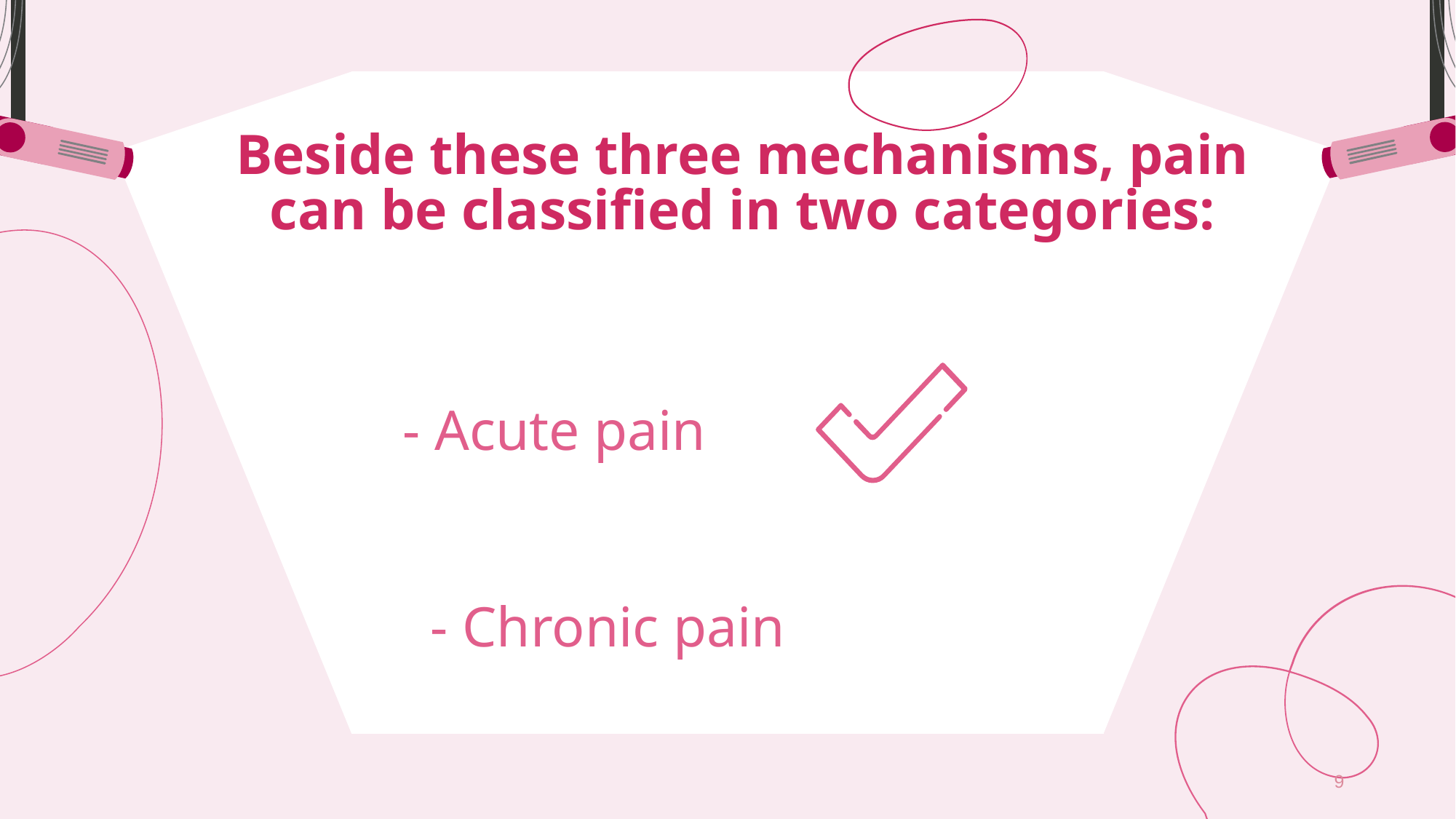

# Beside these three mechanisms, pain can be classified in two categories:
- Acute pain
- Chronic pain
9

## Slide 10
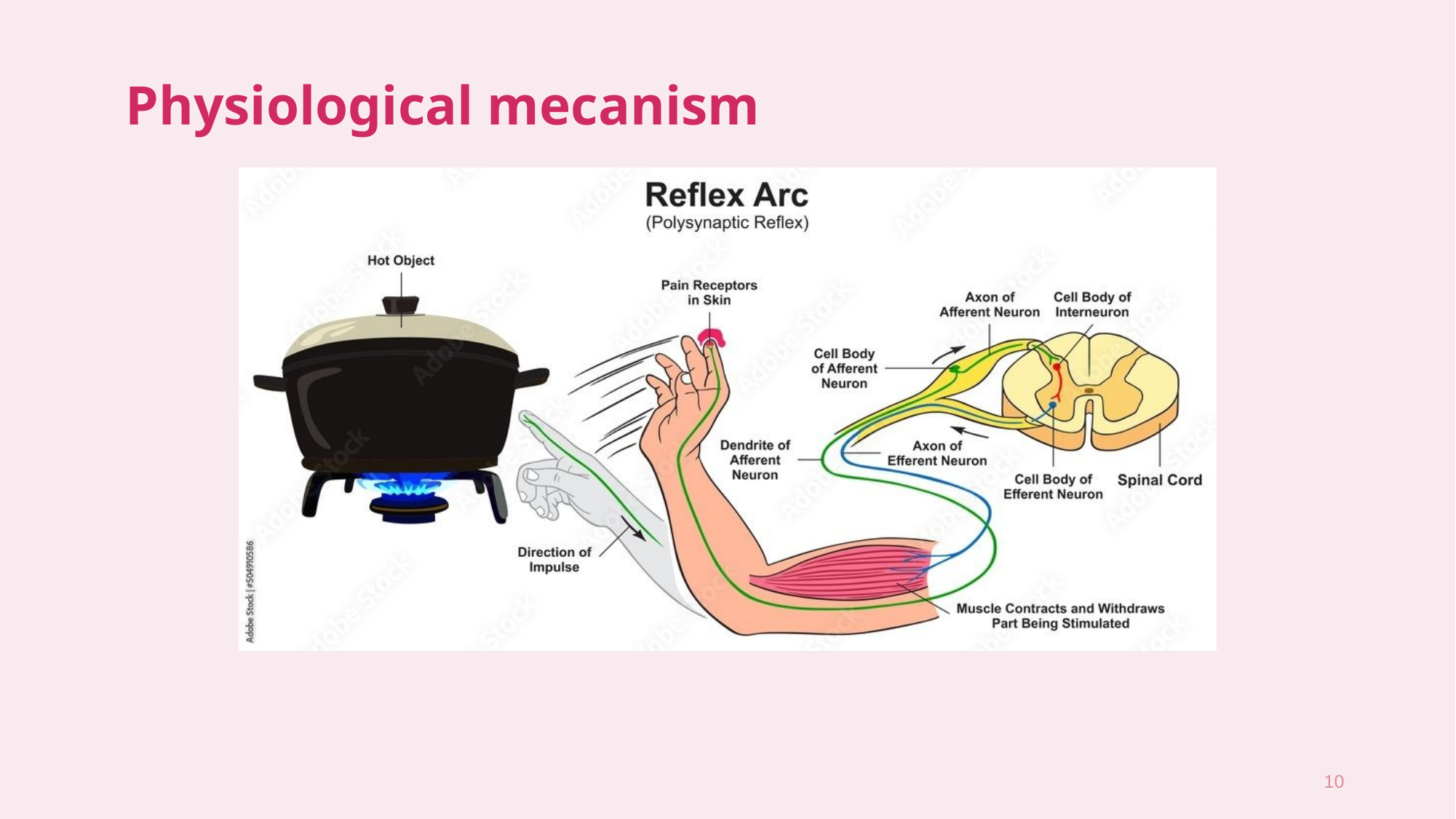

# Physiological mecanism
10

## Slide 11
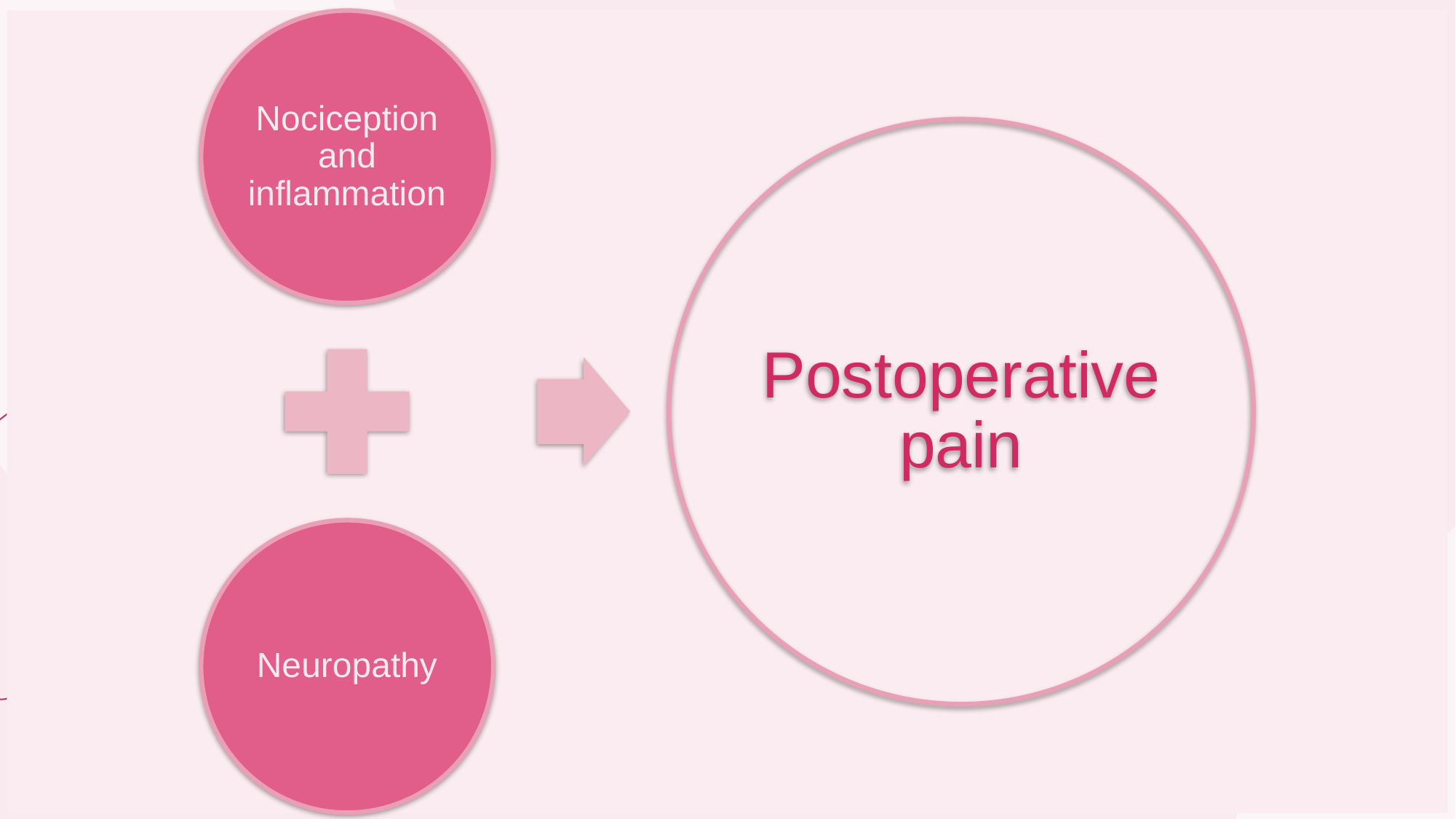

# Pain and surgery
Postoperative pain is provoqued by:
Tissue trauma  Inflammatory reaction
Peripheral nerve injury
Secondary hyperalgesia
This pain is :
Acute
Predictable
Foreseeable
Quantifiable
11

## Slide 12
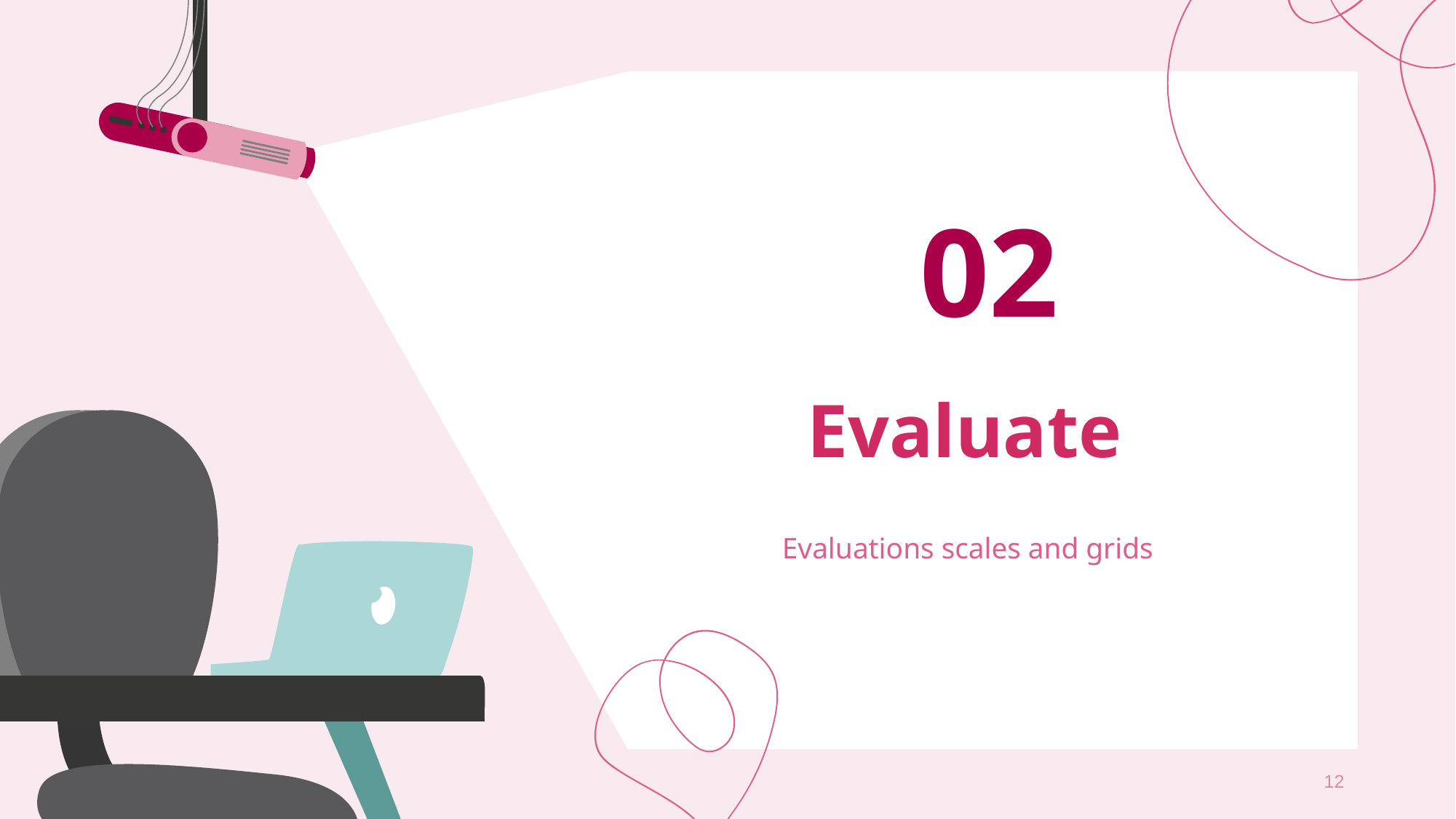

02
# Evaluate
Evaluations scales and grids
12

## Slide 13
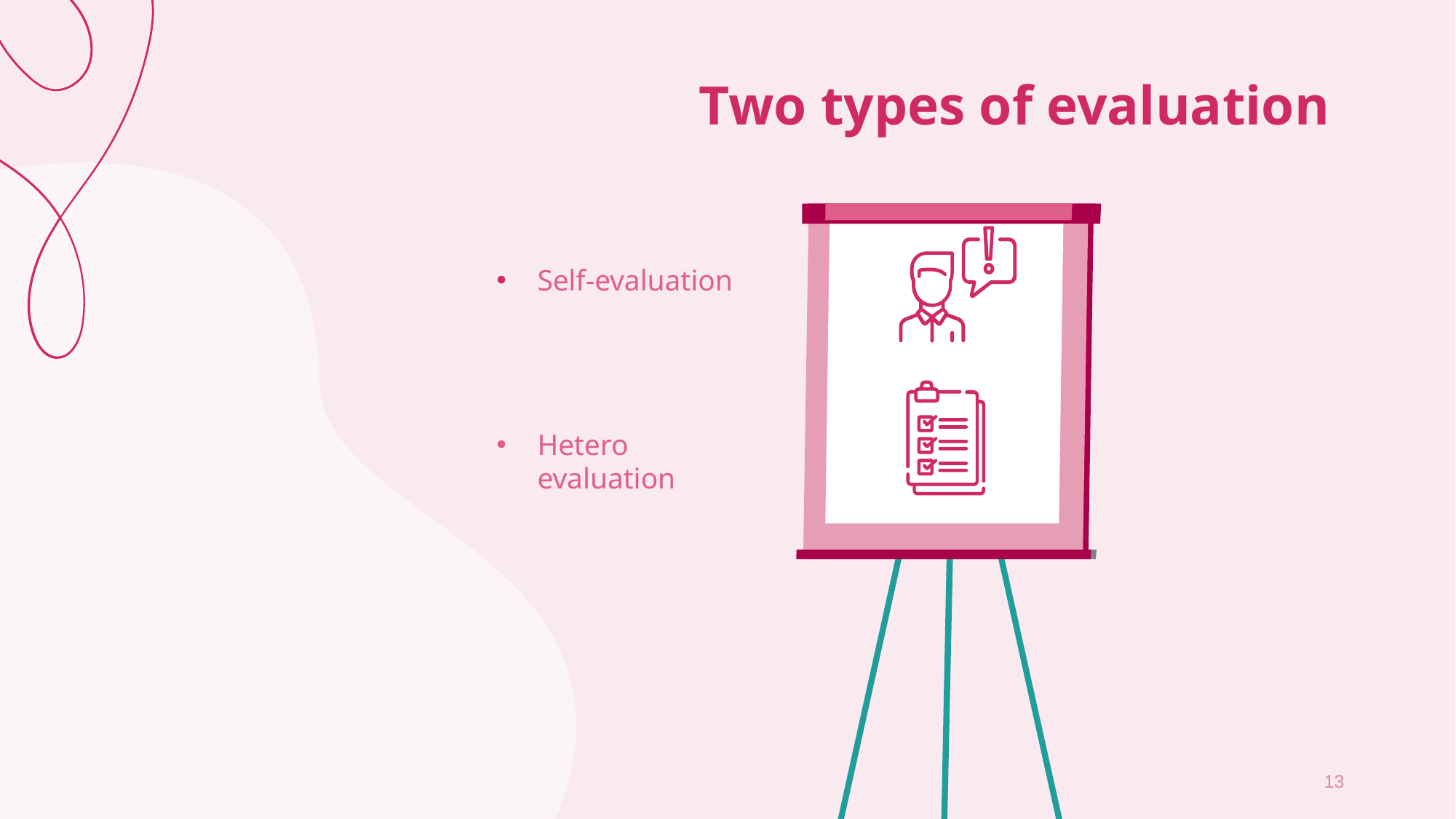

# Two types of evaluation
Self-evaluation
Hetero evaluation
13

## Slide 14
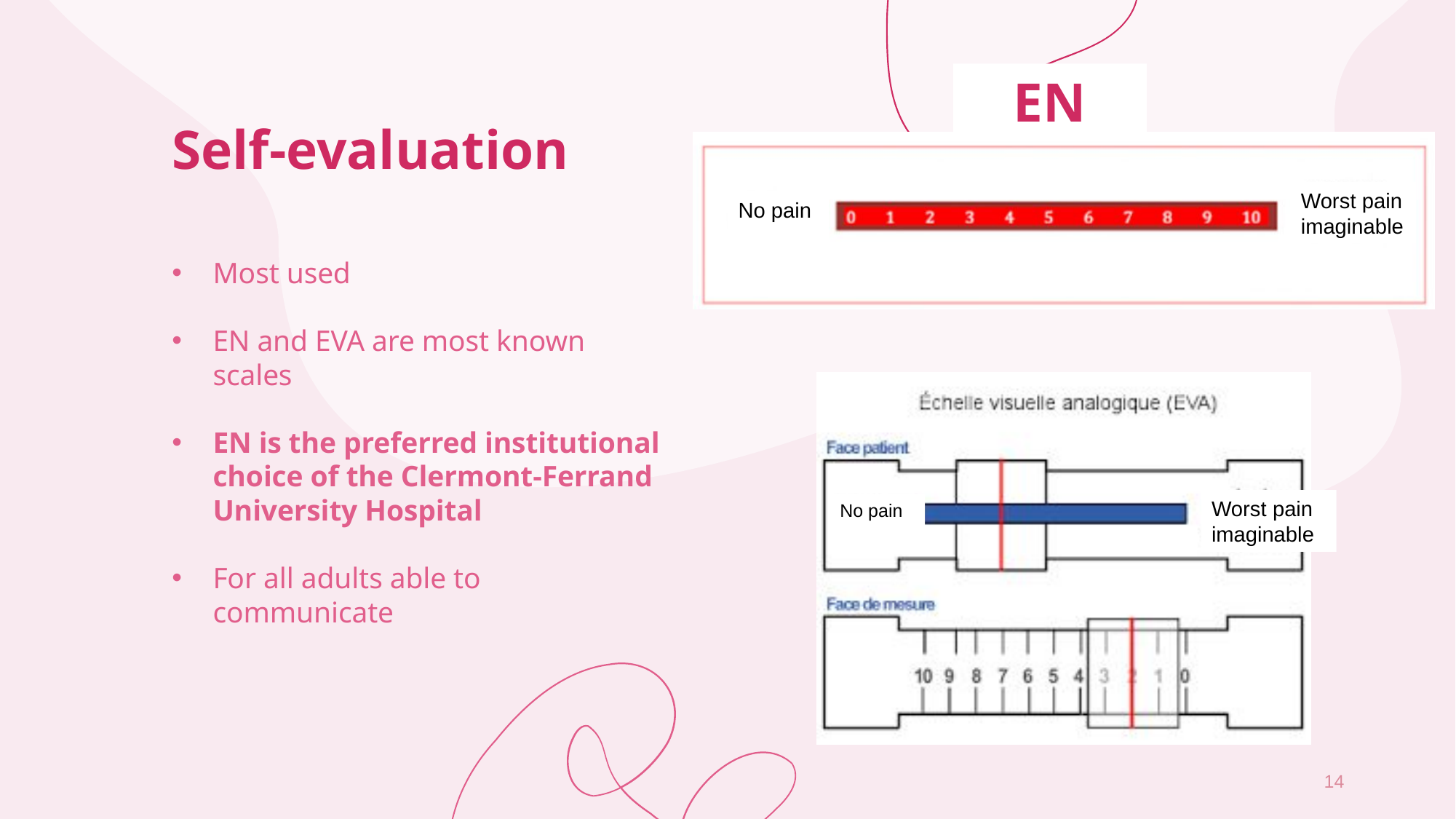

EN
# Self-evaluation
Worst pain imaginable
No pain
Most used
EN and EVA are most known scales
EN is the preferred institutional choice of the Clermont-Ferrand University Hospital
For all adults able to communicate
Worst pain imaginable
No pain
14

## Slide 15
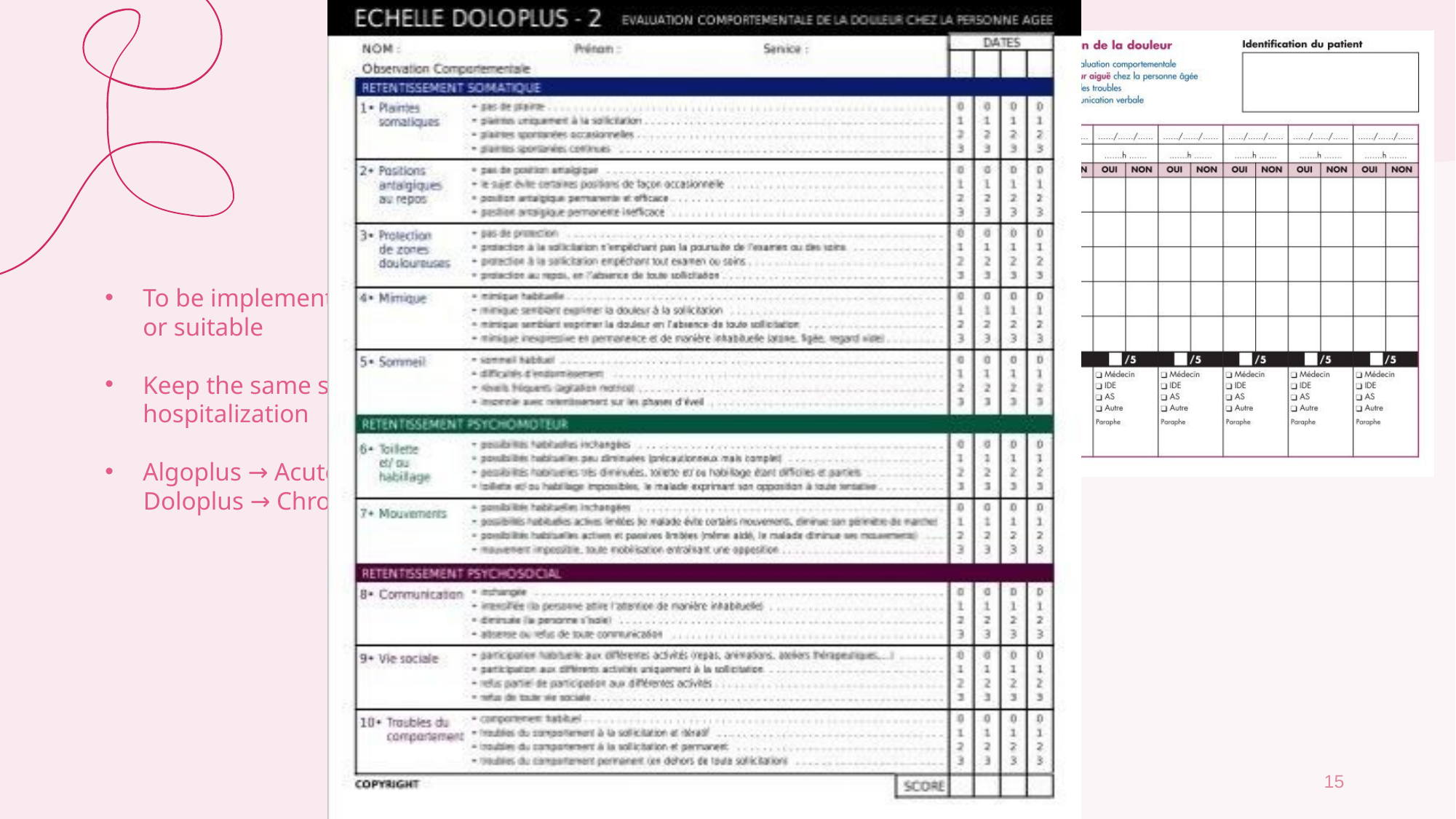

# Hetero evaluation
To be implemented if self-evaluation is not reliable or suitable
Keep the same scale until the end of the hospitalization
Algoplus → Acute pain
 Doloplus → Chronic pain
15

## Slide 16
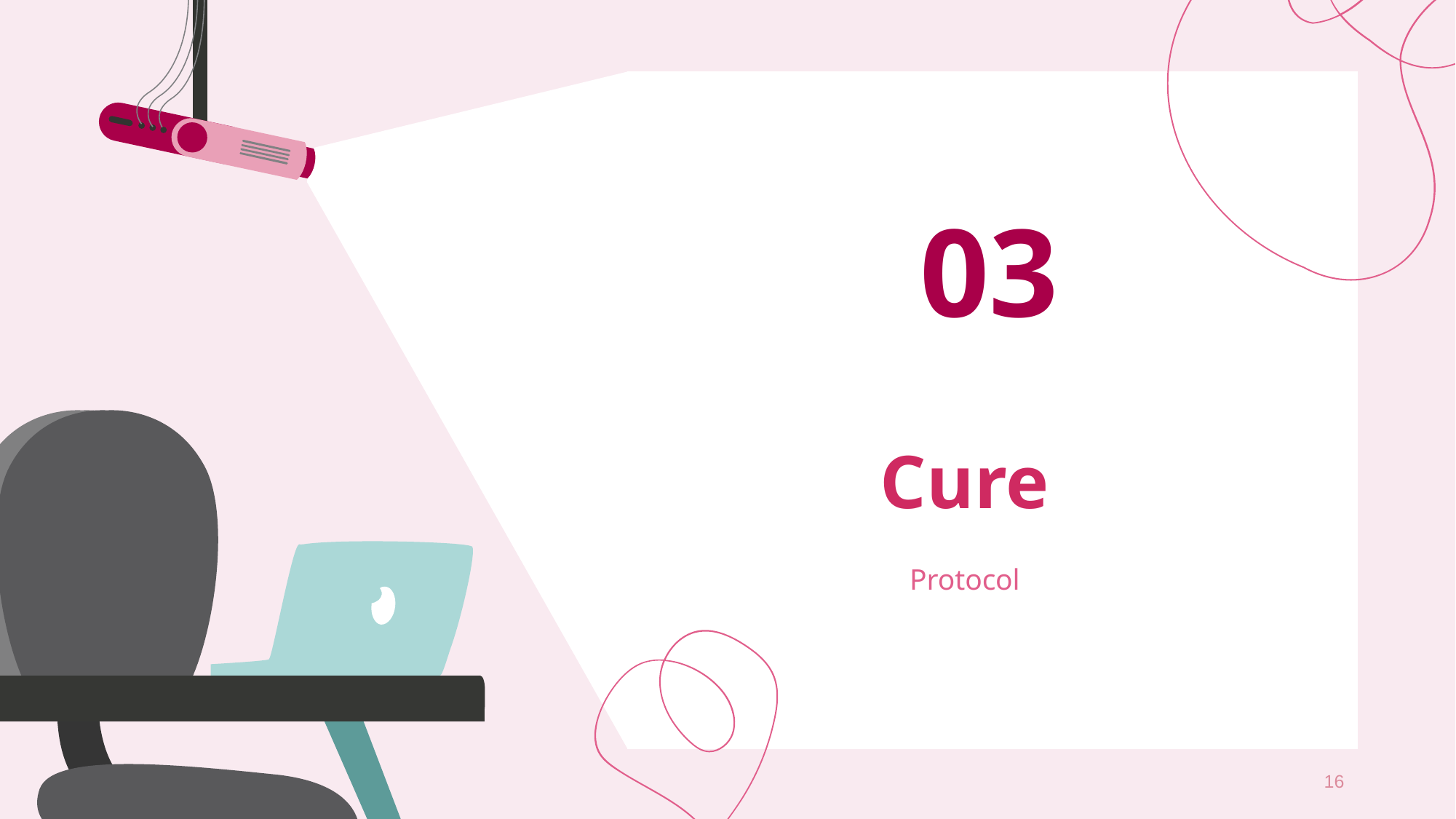

03
# Cure
Protocol
16

## Slide 17
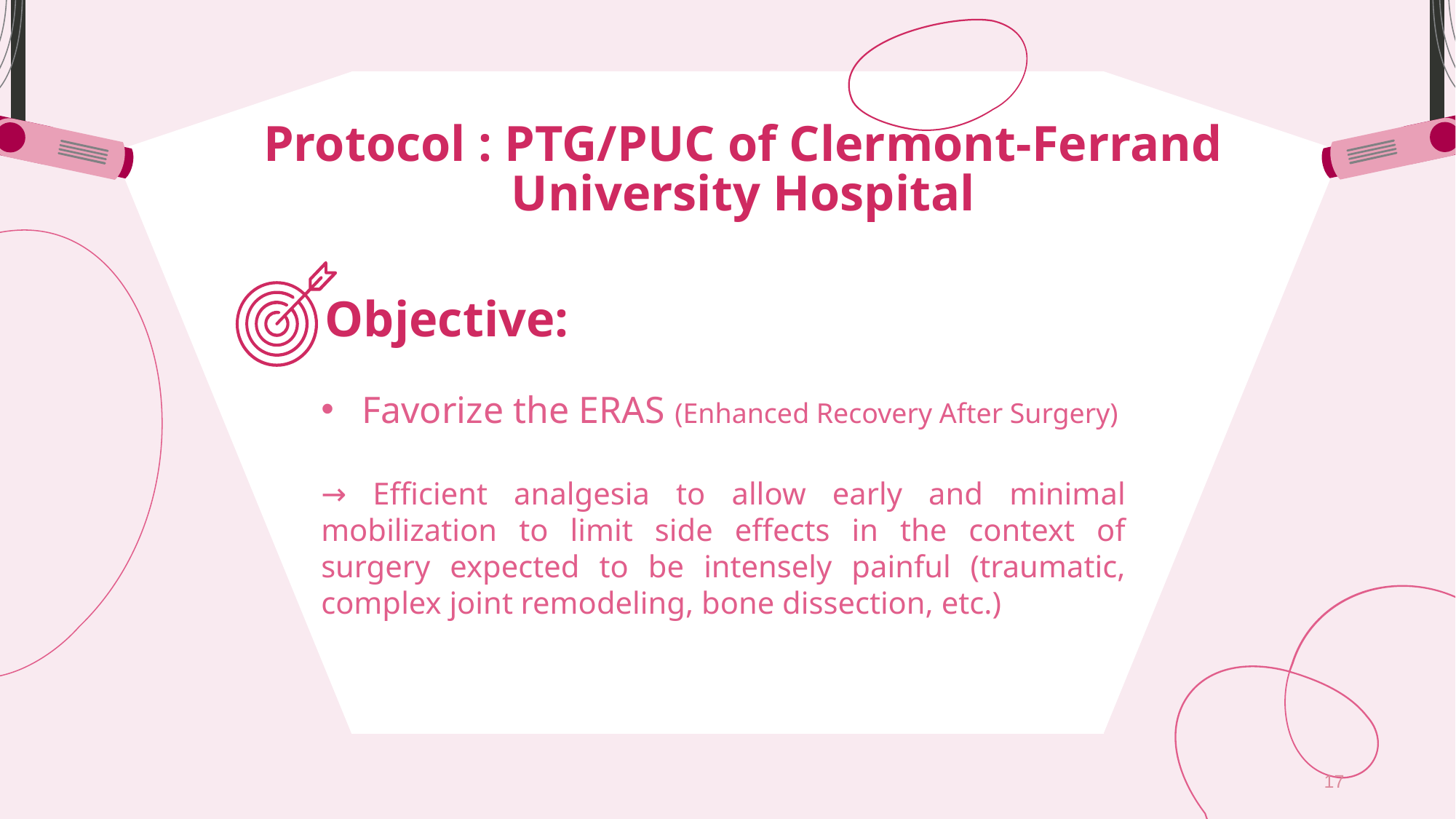

# Protocol : PTG/PUC of Clermont-Ferrand University Hospital
Objective:
Favorize the ERAS (Enhanced Recovery After Surgery)
→ Efficient analgesia to allow early and minimal mobilization to limit side effects in the context of surgery expected to be intensely painful (traumatic, complex joint remodeling, bone dissection, etc.)
17

## Slide 18
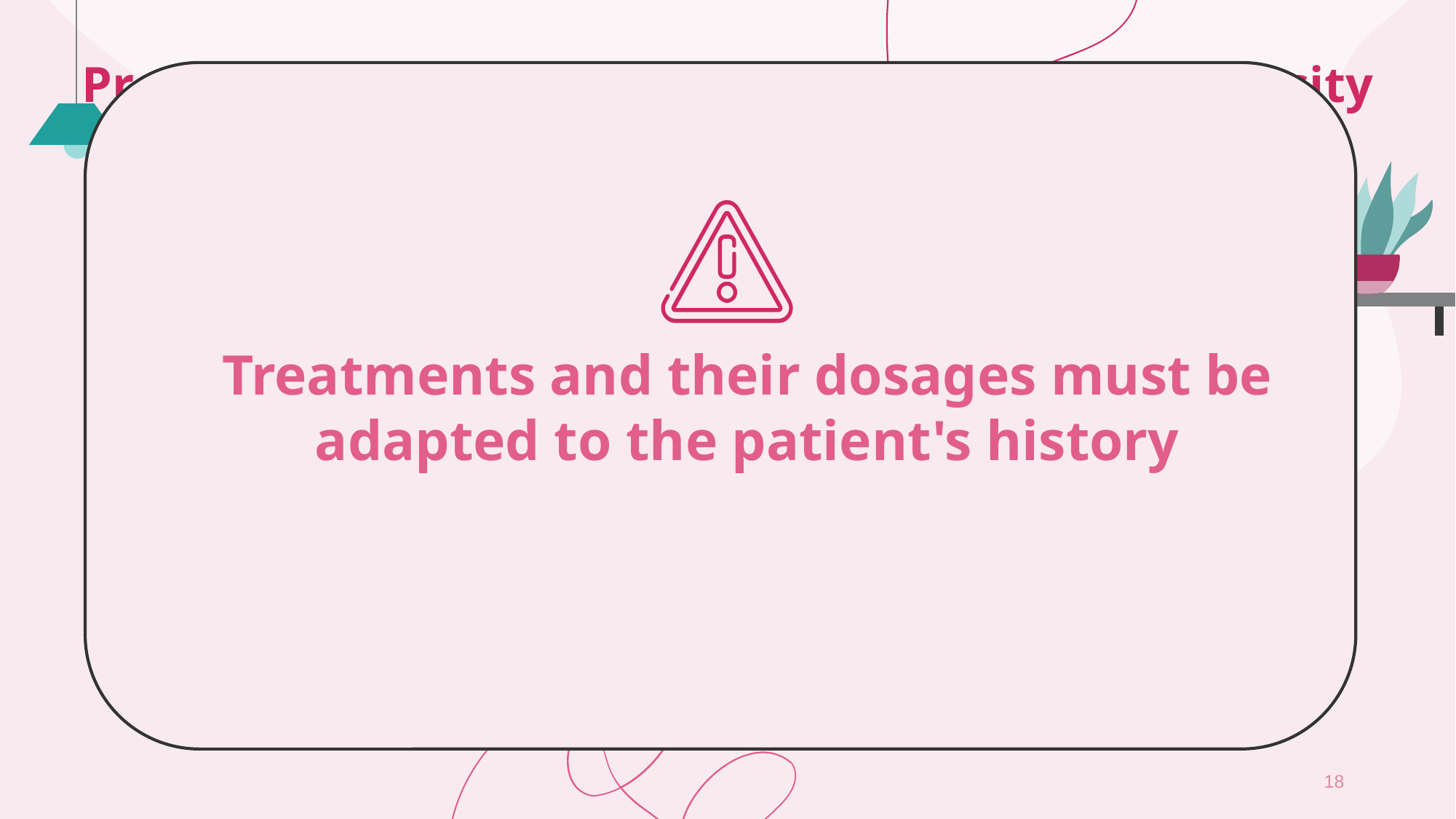

Treatments and their dosages must be adapted to the patient's history
Protocol «  PTG/PUC » of Clermont-Ferrand University Hospital
Medical Treatment:
Everytime :
Paracetamol IV maximum dosage
Nefopam IV 20 mg
Ketoprofen IV 100 mg
(Single dose possible in case of contraindication)
Dexamethasone 0,15mg/kg
Morphine IV:
At the end of surgery: depending the used opioid during surgery.
(If remifentanil : morphine IV 0,15mg/kg of normalized body weight)
Spinal Analgesia : anticipate the end of effects with morphine IV ≤ 0,15mg/kg
Recovery Room : Morphine titration up to 0,15mg/kg
18

## Slide 19
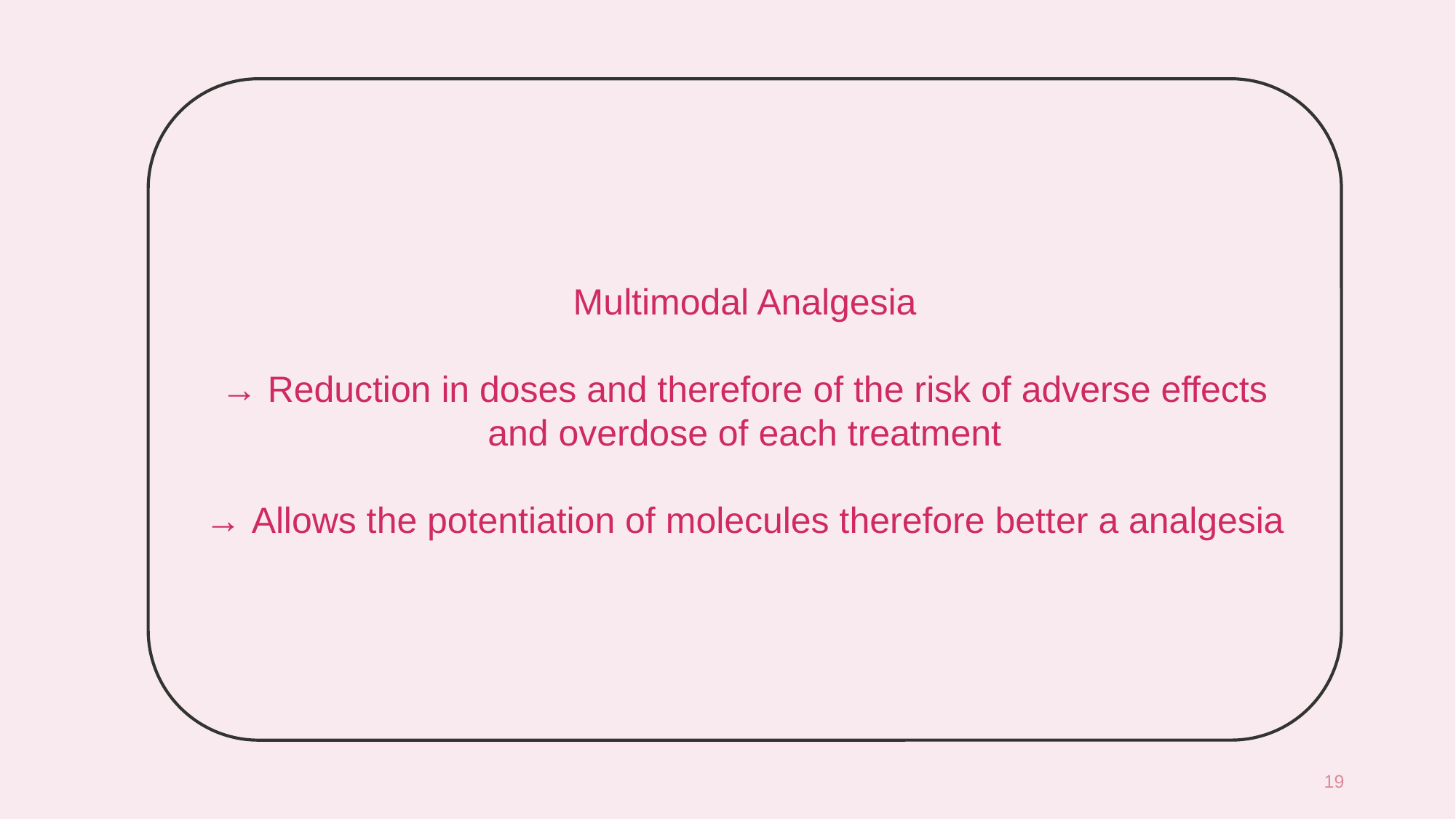

# WHO’s scale
Multimodal Analgesia
→ Reduction in doses and therefore of the risk of adverse effects and overdose of each treatment
→ Allows the potentiation of molecules therefore better a analgesia
THIRD STEP
SECOND STEP
“Strong” Opioid Painkillers
Morphine
Fentanyl
Hydromorphone
Oxycodone
FIRST STEP
“Weak” Opioid Painkillers
Codeine
Dextropropoyphene
Tramadol
Non-Opioid Painkillers
Paracetamol
Aspirin
NSAID
MILD PAIN
MILD TO MODERATE PAIN
MODERATE TO SEVERE PAIN
1+ 2
Synergistic association
1+ 3
19

## Slide 20
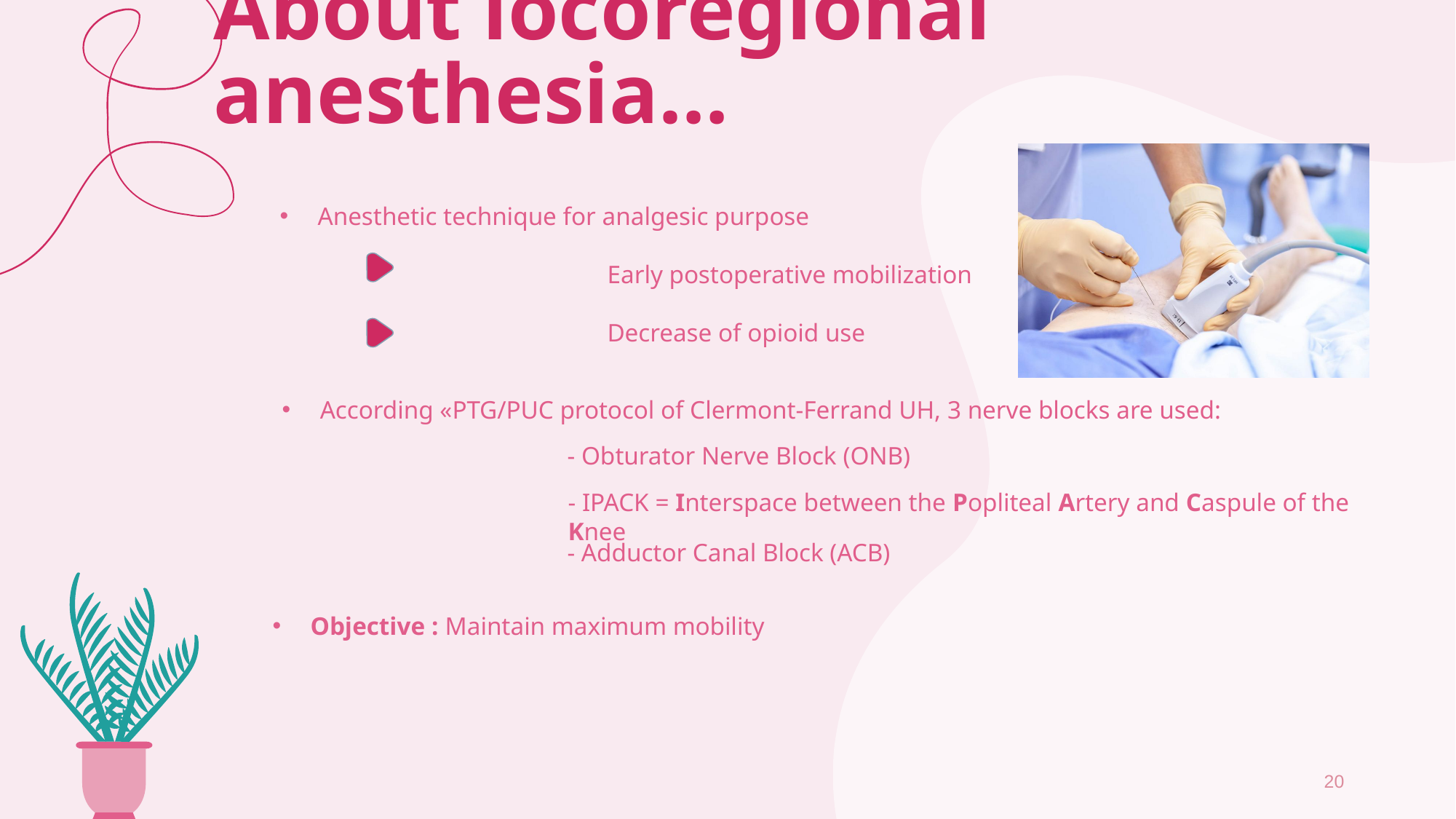

# About locoregional anesthesia…
Anesthetic technique for analgesic purpose
			Early postoperative mobilization
			Decrease of opioid use
According «PTG/PUC protocol of Clermont-Ferrand UH, 3 nerve blocks are used:
- Obturator Nerve Block (ONB)
- IPACK = Interspace between the Popliteal Artery and Caspule of the Knee
- Adductor Canal Block (ACB)
Objective : Maintain maximum mobility
20

## Slide 21
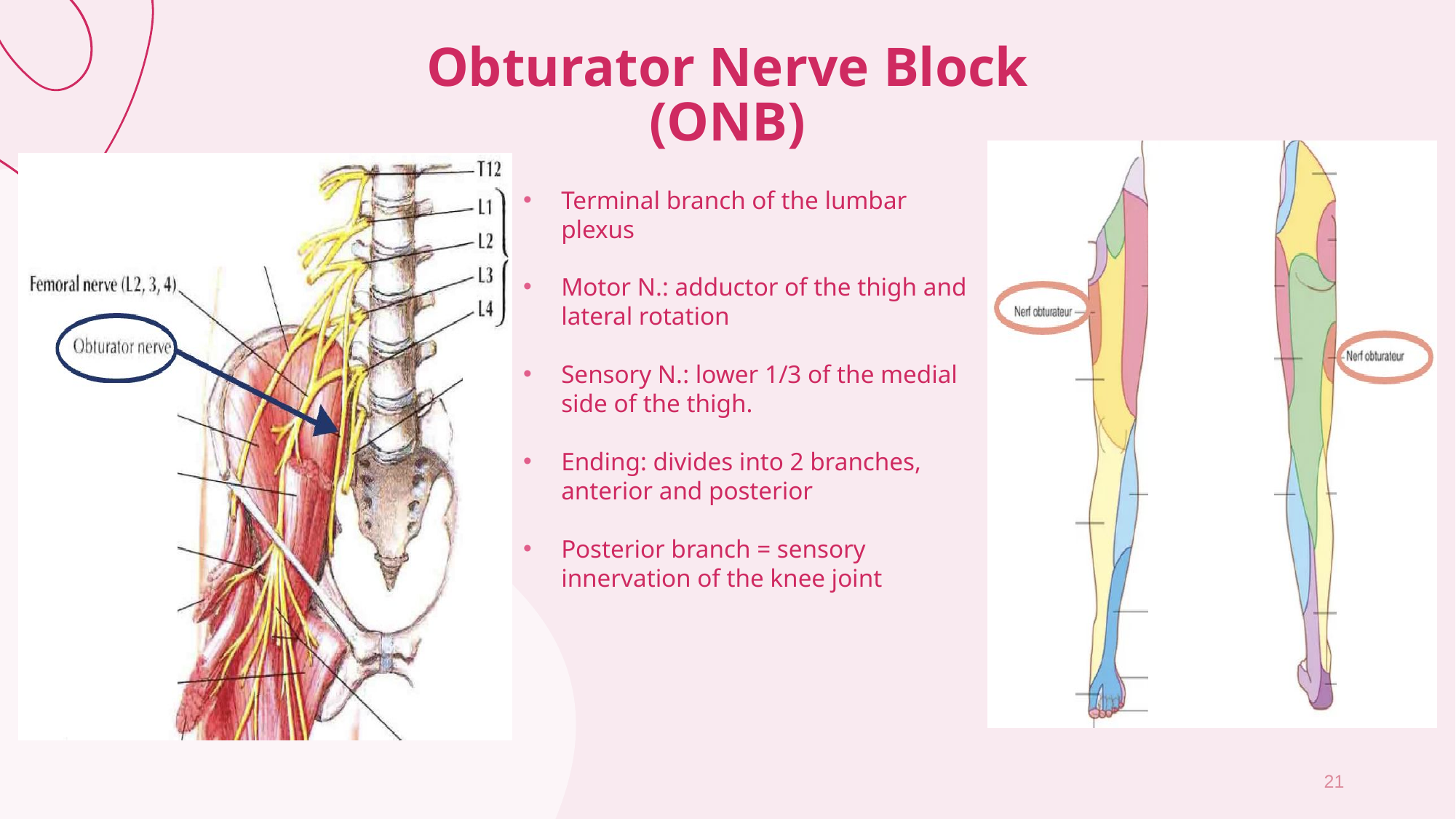

# Obturator Nerve Block (ONB)
Terminal branch of the lumbar plexus
Motor N.: adductor of the thigh and lateral rotation
Sensory N.: lower 1/3 of the medial side of the thigh.
Ending: divides into 2 branches, anterior and posterior
Posterior branch = sensory innervation of the knee joint
21

## Slide 22
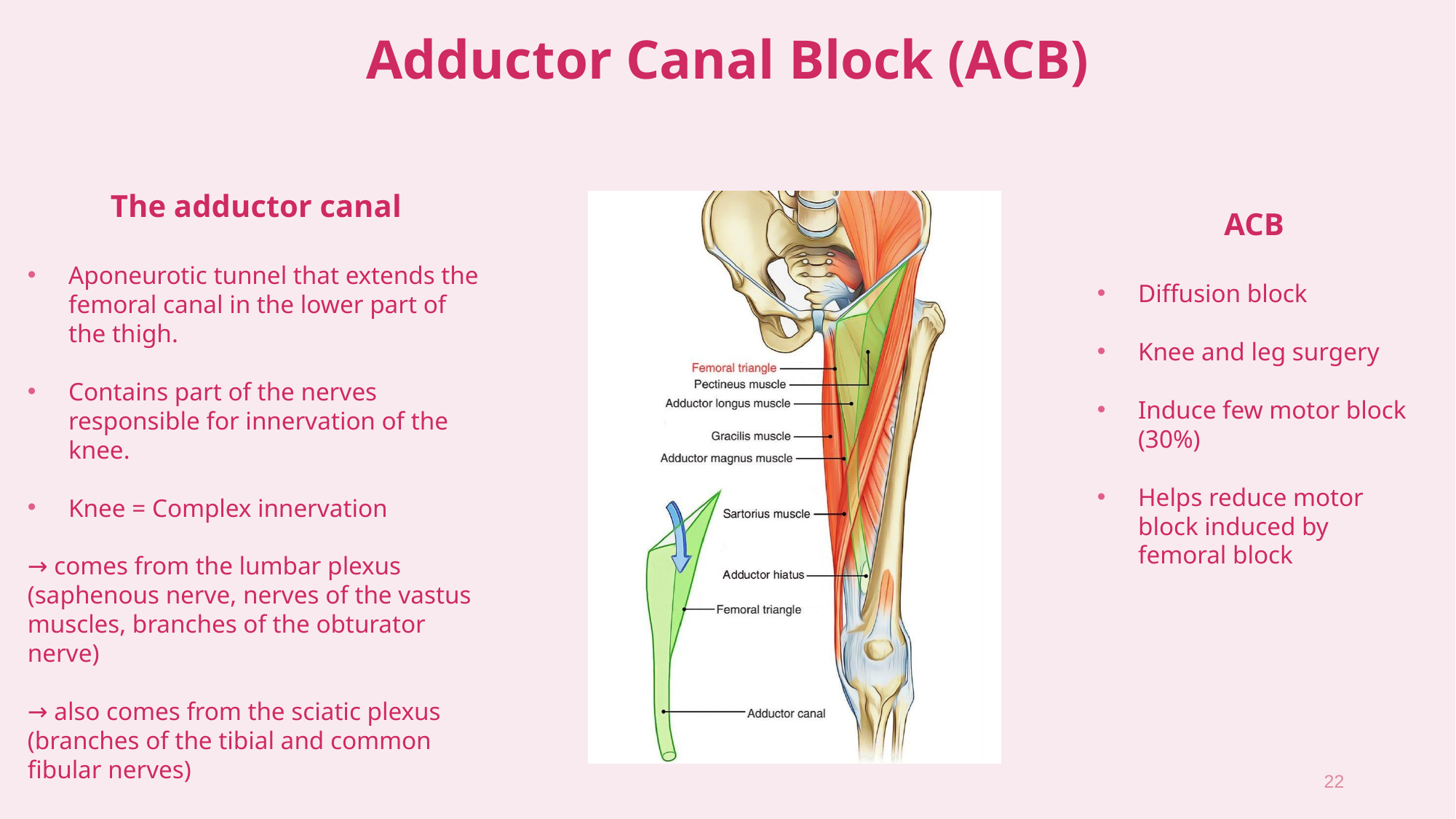

# Adductor Canal Block (ACB)
The adductor canal
Aponeurotic tunnel that extends the femoral canal in the lower part of the thigh.
Contains part of the nerves responsible for innervation of the knee.
Knee = Complex innervation
→ comes from the lumbar plexus (saphenous nerve, nerves of the vastus muscles, branches of the obturator nerve)
→ also comes from the sciatic plexus (branches of the tibial and common fibular nerves)
ACB
Diffusion block
Knee and leg surgery
Induce few motor block (30%)
Helps reduce motor block induced by femoral block
22

## Slide 23
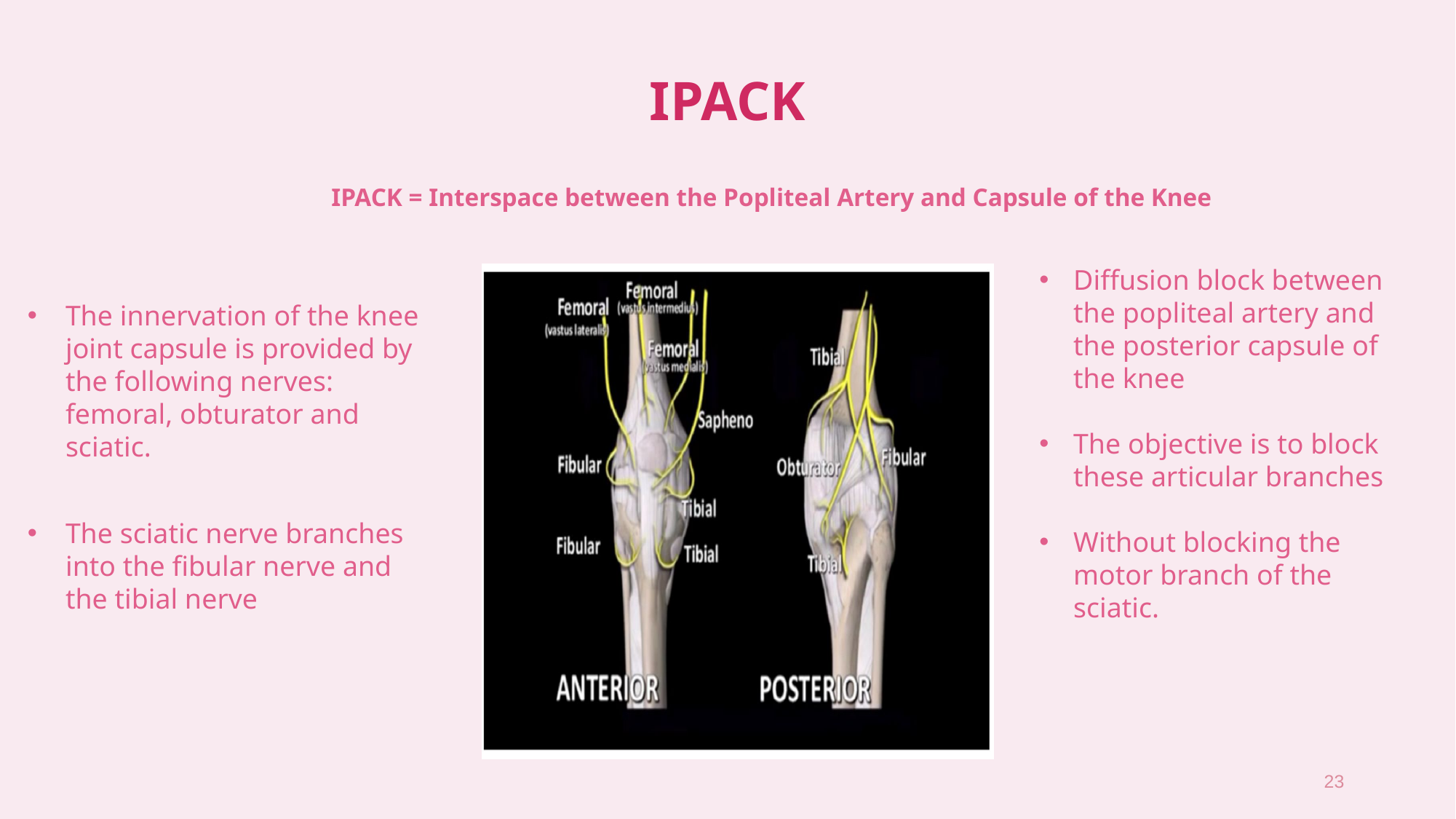

# IPACK
IPACK = Interspace between the Popliteal Artery and Capsule of the Knee
The innervation of the knee joint capsule is provided by the following nerves: femoral, obturator and sciatic.
The sciatic nerve branches into the fibular nerve and the tibial nerve
Diffusion block between the popliteal artery and the posterior capsule of the knee
The objective is to block these articular branches
Without blocking the motor branch of the sciatic.
23

## Slide 24
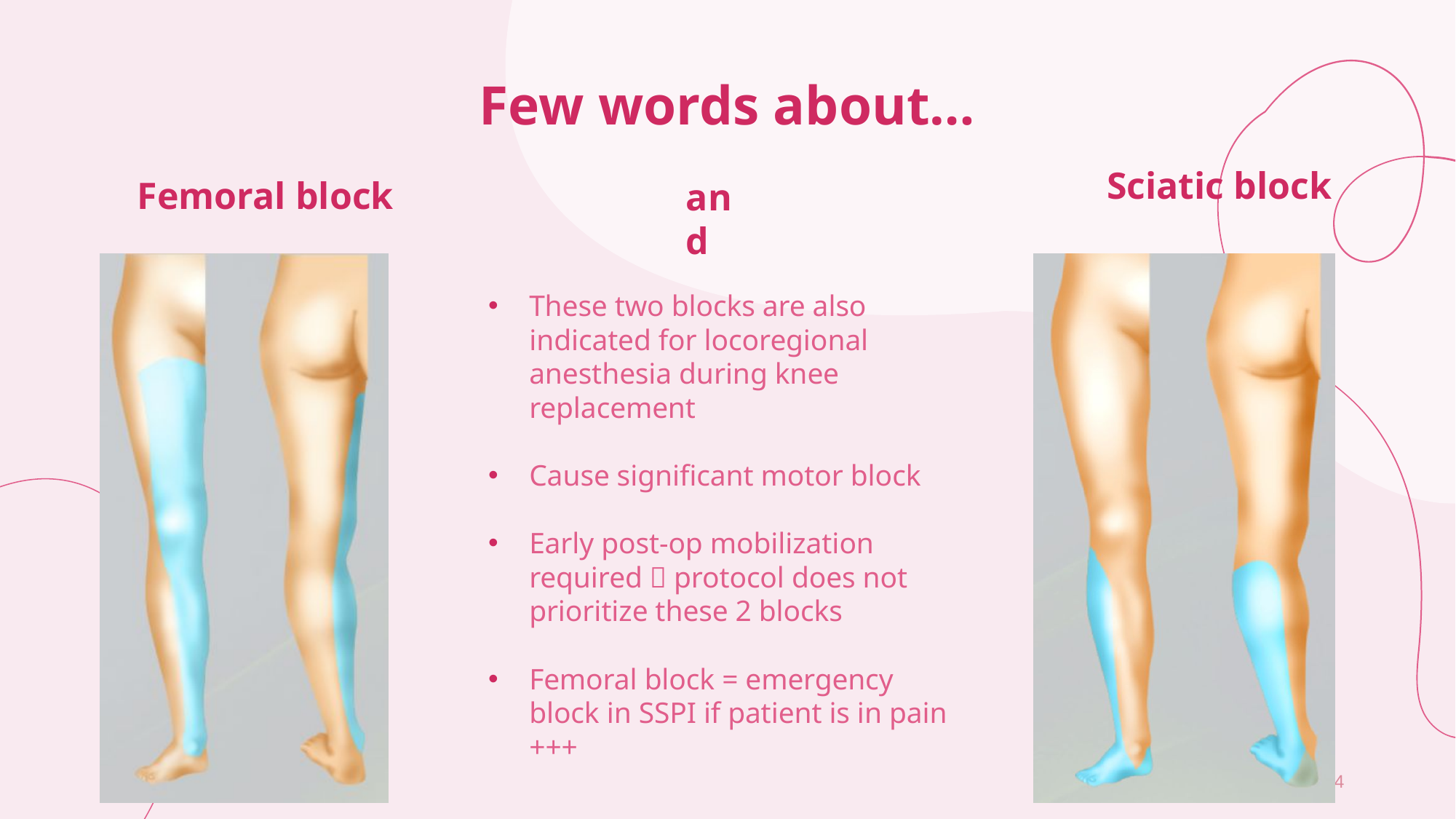

# Few words about…
Sciatic block
Femoral block
and
These two blocks are also indicated for locoregional anesthesia during knee replacement
Cause significant motor block
Early post-op mobilization required  protocol does not prioritize these 2 blocks
Femoral block = emergency block in SSPI if patient is in pain +++
24

## Slide 25
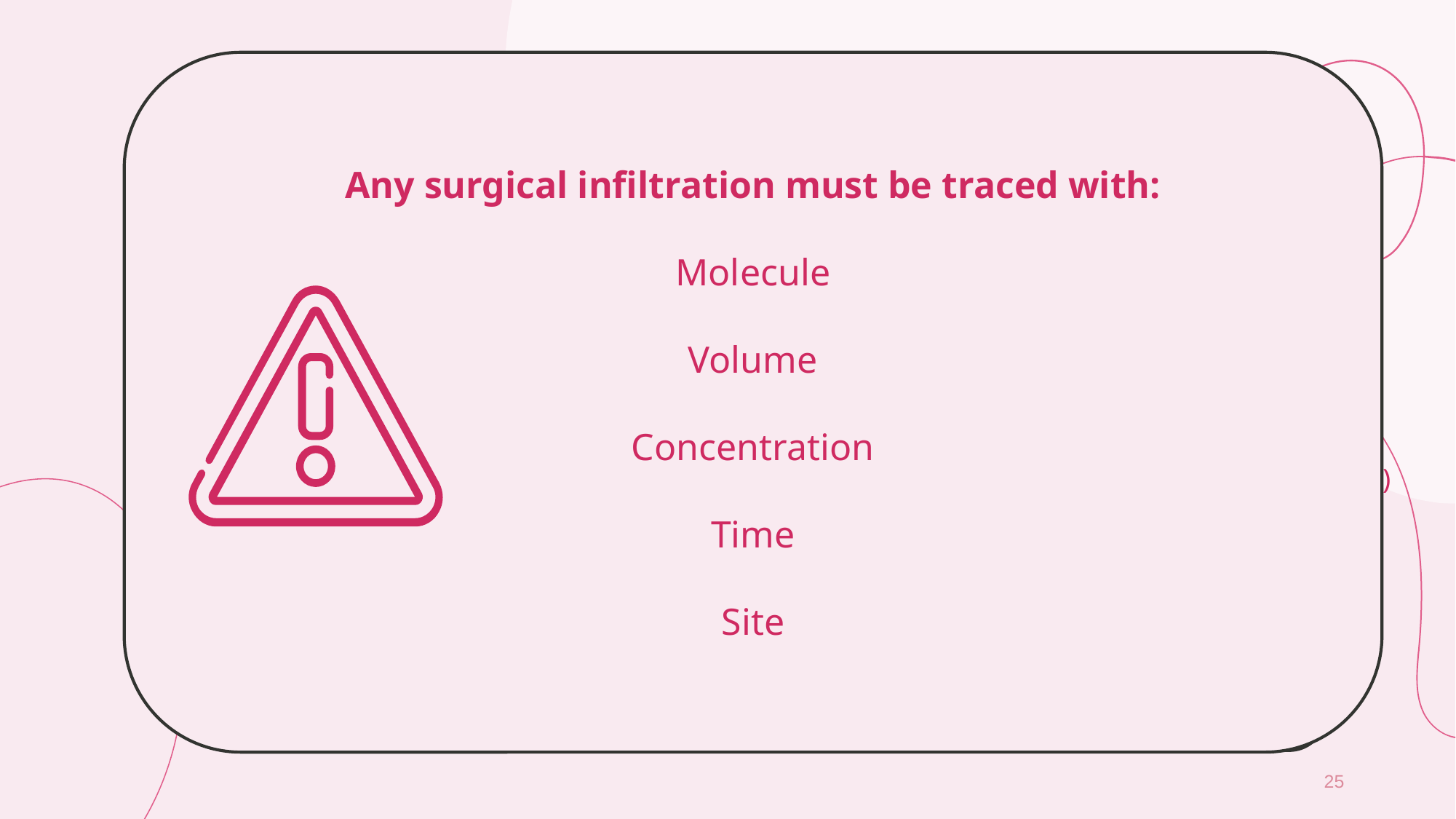

# Locoregional anesthesia traceability
Any surgical infiltration must be traced with:
Molecule
Volume
Concentration
Time
Site
Must be noted on the anesthesia sheet:
Time of block
Name of Block
Local Anesthetic used:
	→ volume
	→ Concentration
Puncture site
Lateralization (side)
Needle used
Aspiration test
Any incident
(Technique used (ultrasound, etc.))
96 % of Anesthetist Nurses ensure that the block is traced
72% of them ensure that the 8 criteria above appear
25

## Slide 26
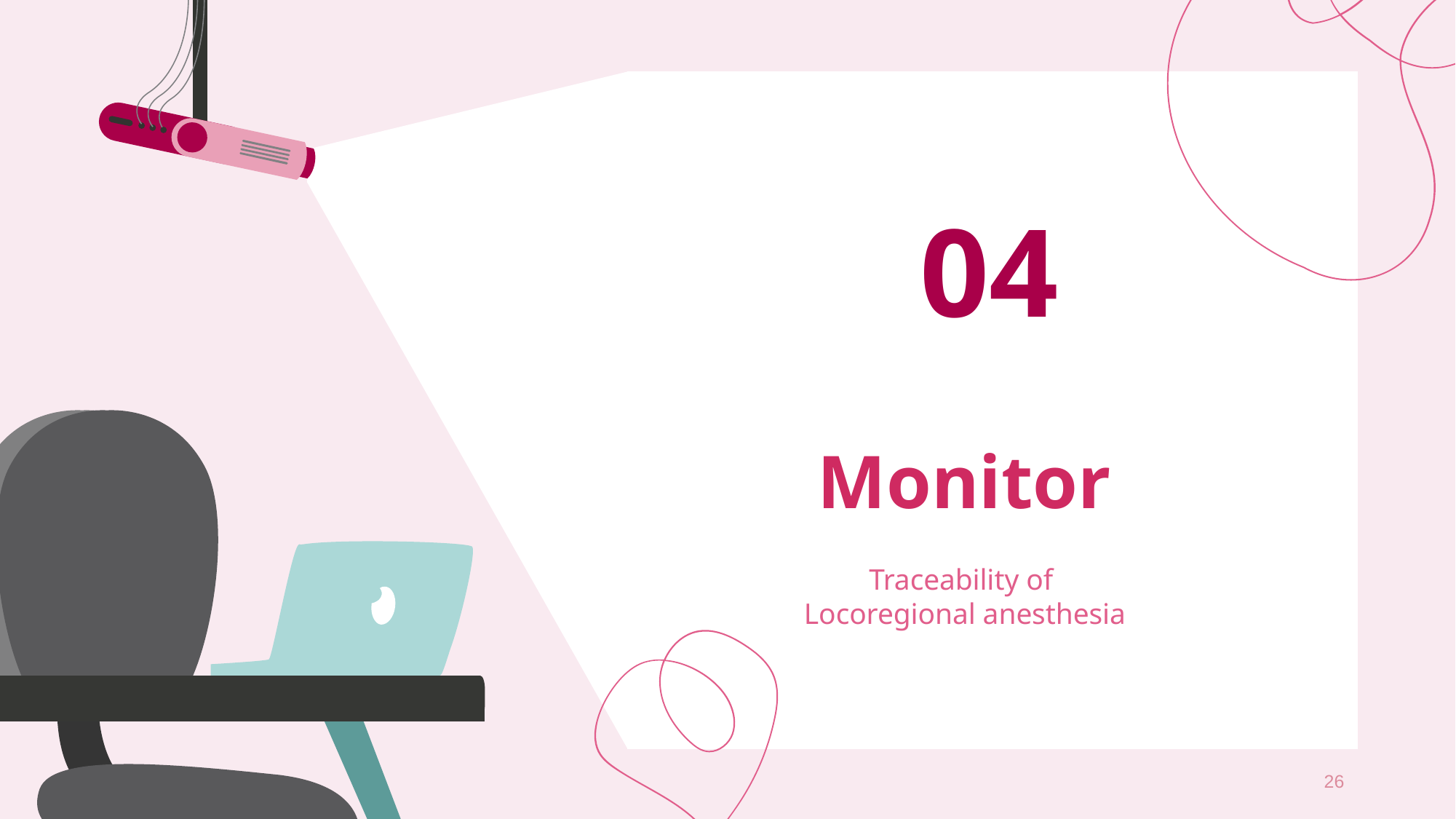

04
# Monitor
Traceability of
Locoregional anesthesia
26

## Slide 27
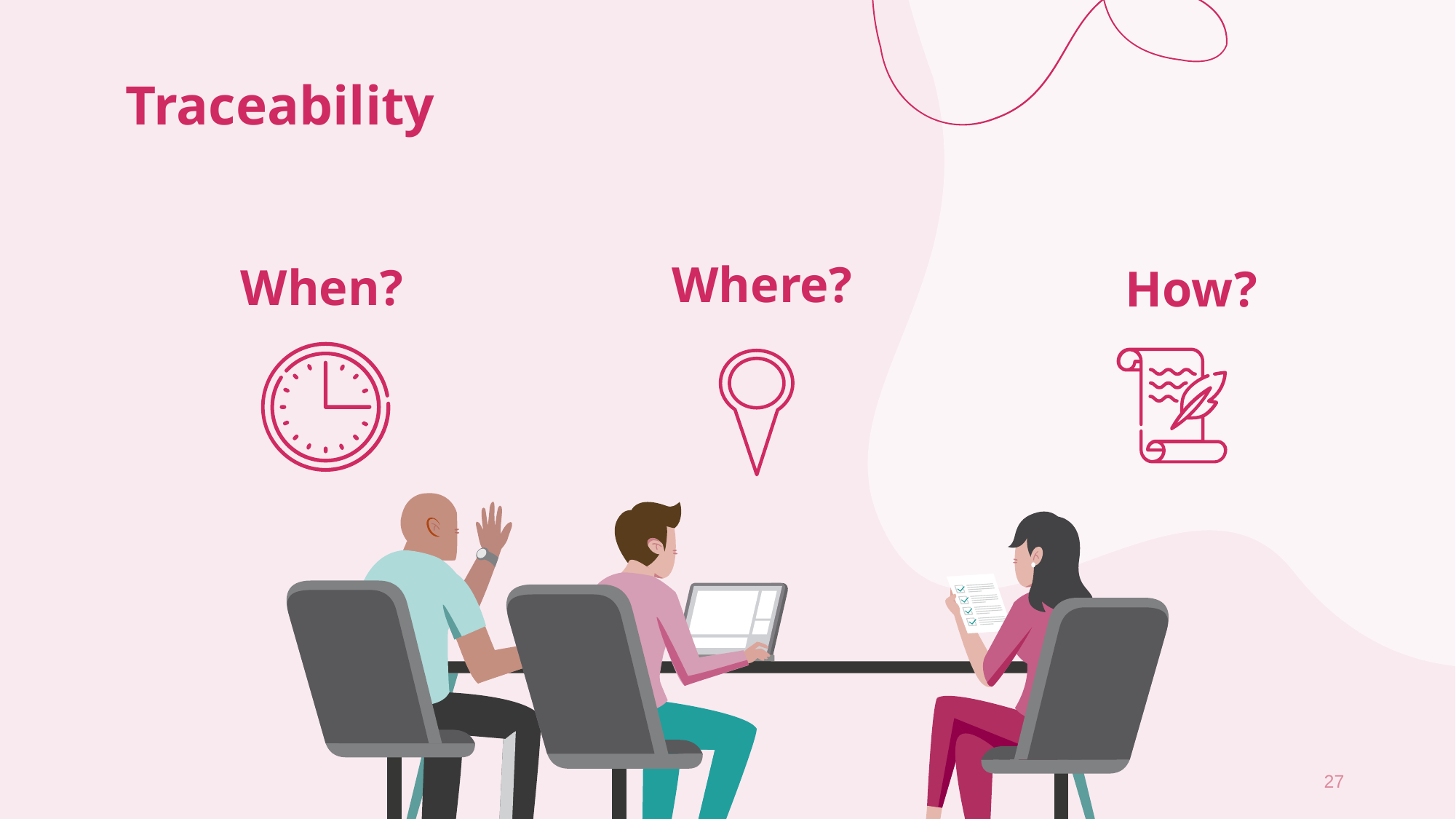

# Traceability
Where?
When?
How?
27

## Slide 28
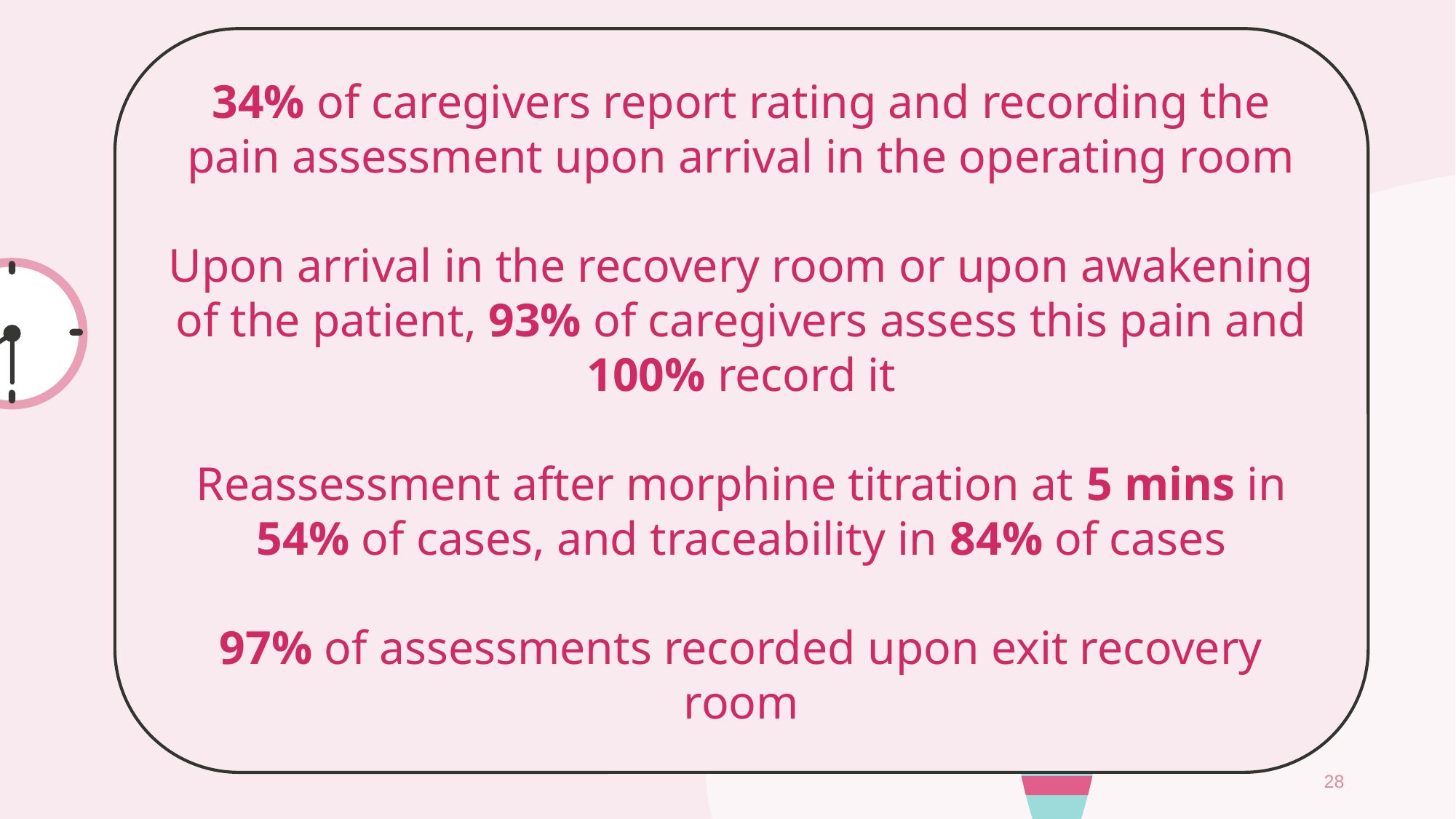

34% of caregivers report rating and recording the pain assessment upon arrival in the operating room
Upon arrival in the recovery room or upon awakening of the patient, 93% of caregivers assess this pain and 100% record it
Reassessment after morphine titration at 5 mins in 54% of cases, and traceability in 84% of cases
97% of assessments recorded upon exit recovery room
# WHEN ?
WHERE ?
Upon admission in the OR
Upon admission in recovery room
At each monitoring
Reassess after analgesic administration
When leaving recovery room
Anesthetic sheet before surgery
Recovery room sheet after surgery
28

## Slide 29
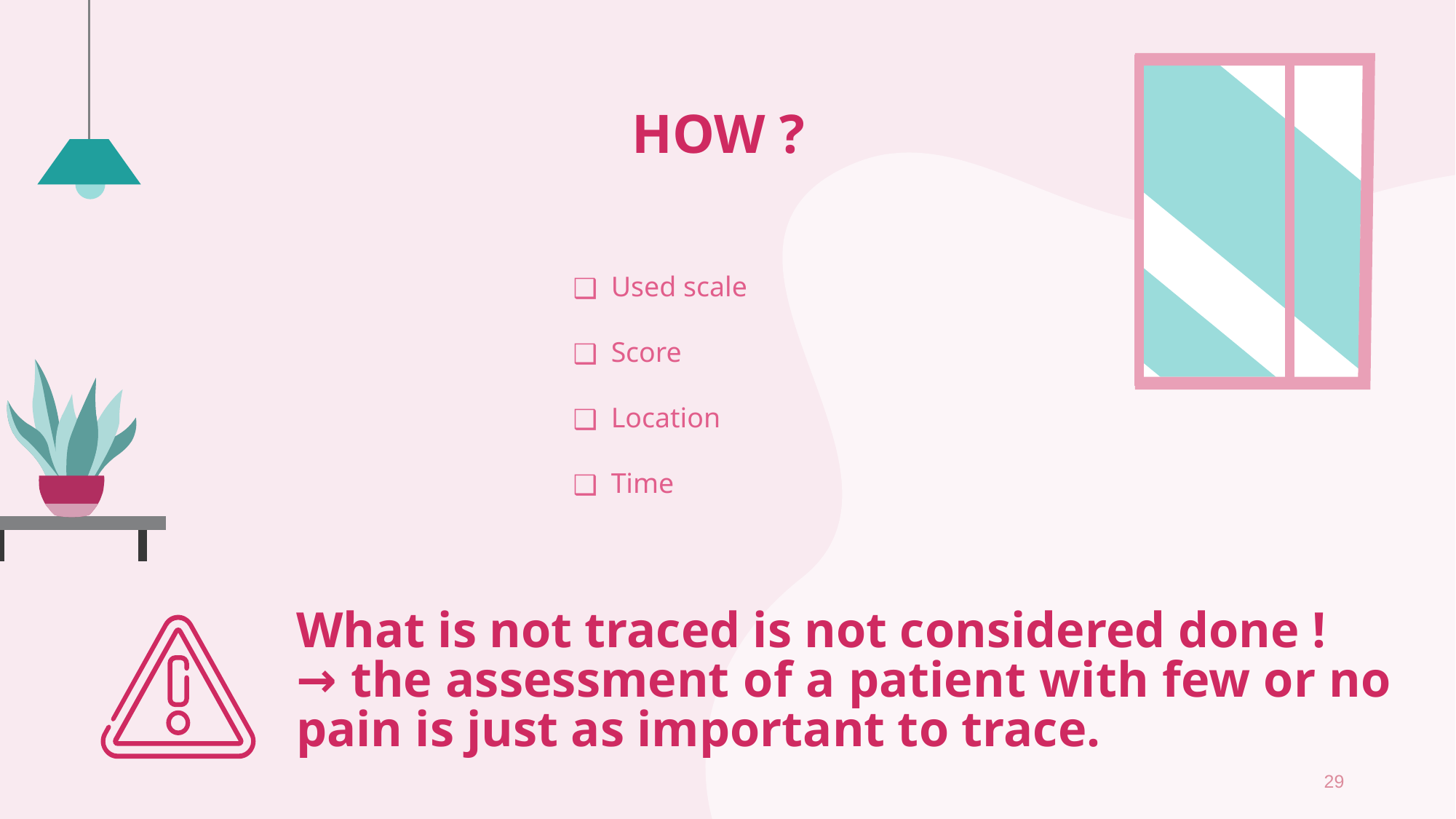

# HOW ?
Used scale
Score
Location
Time
What is not traced is not considered done !
→ the assessment of a patient with few or no pain is just as important to trace.
29

## Slide 30
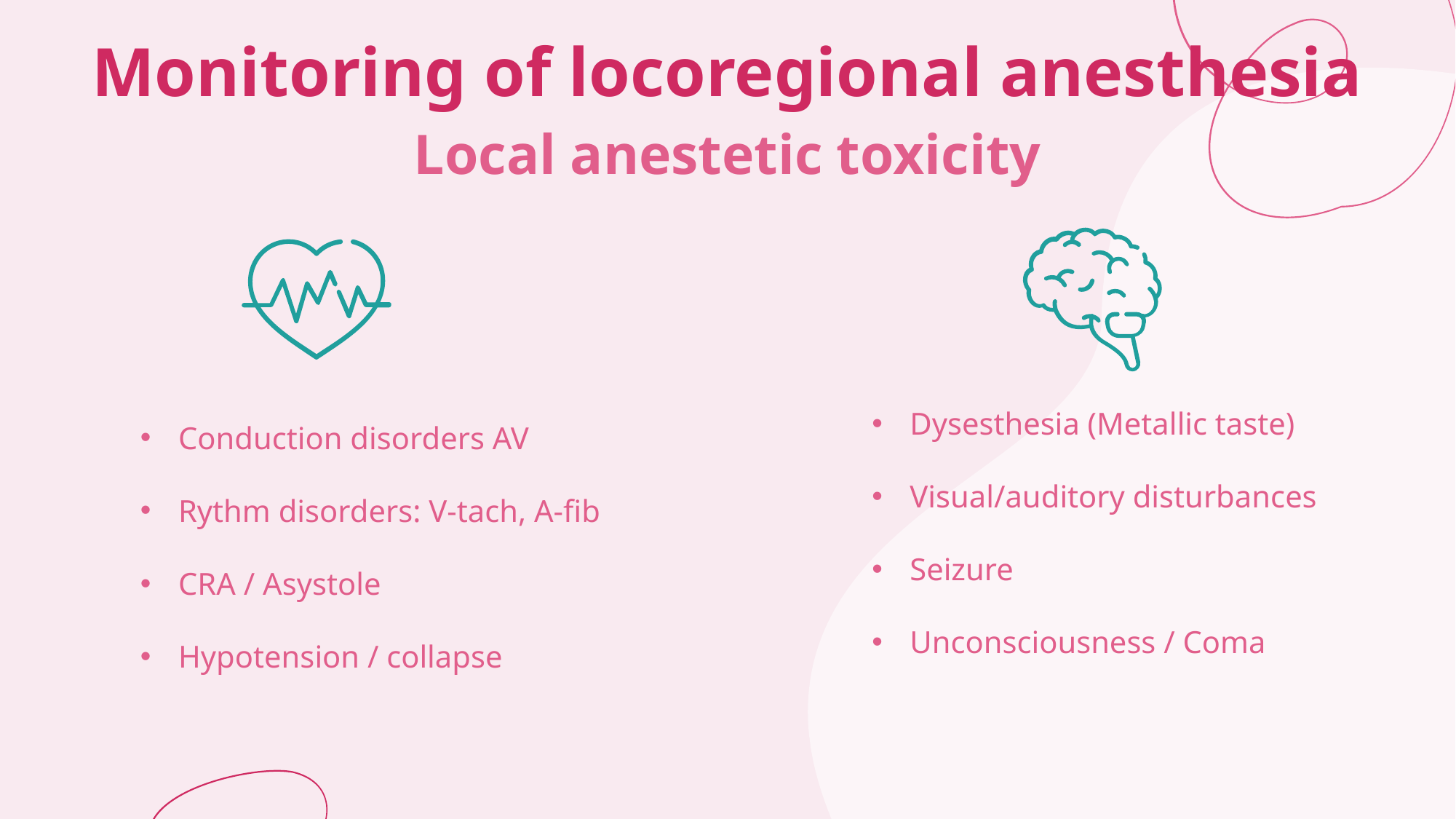

Monitoring of locoregional anesthesia
Local anestetic toxicity
Dysesthesia (Metallic taste)
Visual/auditory disturbances
Seizure
Unconsciousness / Coma
Conduction disorders AV
Rythm disorders: V-tach, A-fib
CRA / Asystole
Hypotension / collapse

## Slide 31
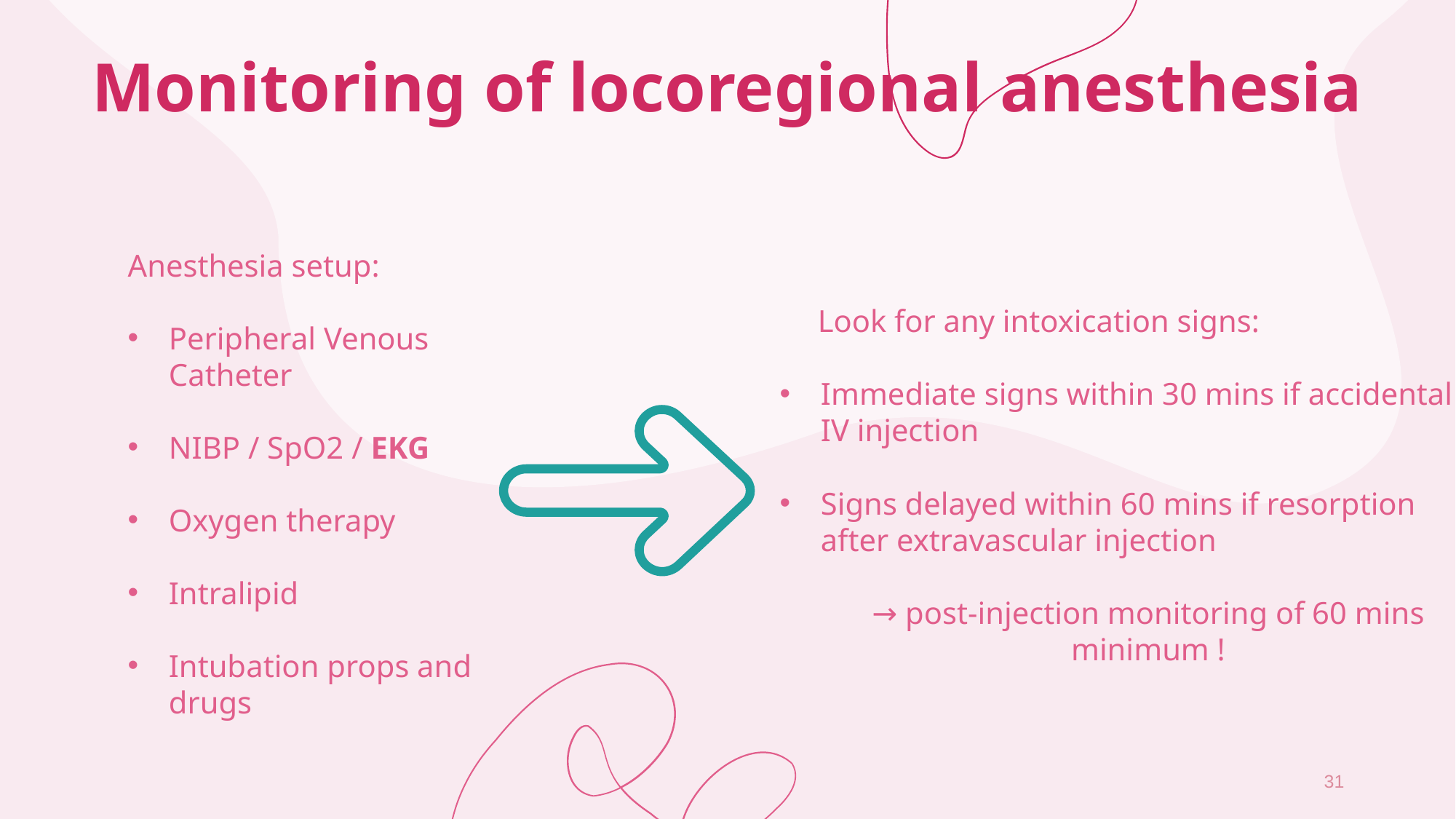

Monitoring of locoregional anesthesia
Anesthesia setup:
Peripheral Venous Catheter
NIBP / SpO2 / EKG
Oxygen therapy
Intralipid
Intubation props and drugs
	Look for any intoxication signs:
Immediate signs within 30 mins if accidental IV injection
Signs delayed within 60 mins if resorption after extravascular injection
→ post-injection monitoring of 60 mins minimum !
31

## Slide 32
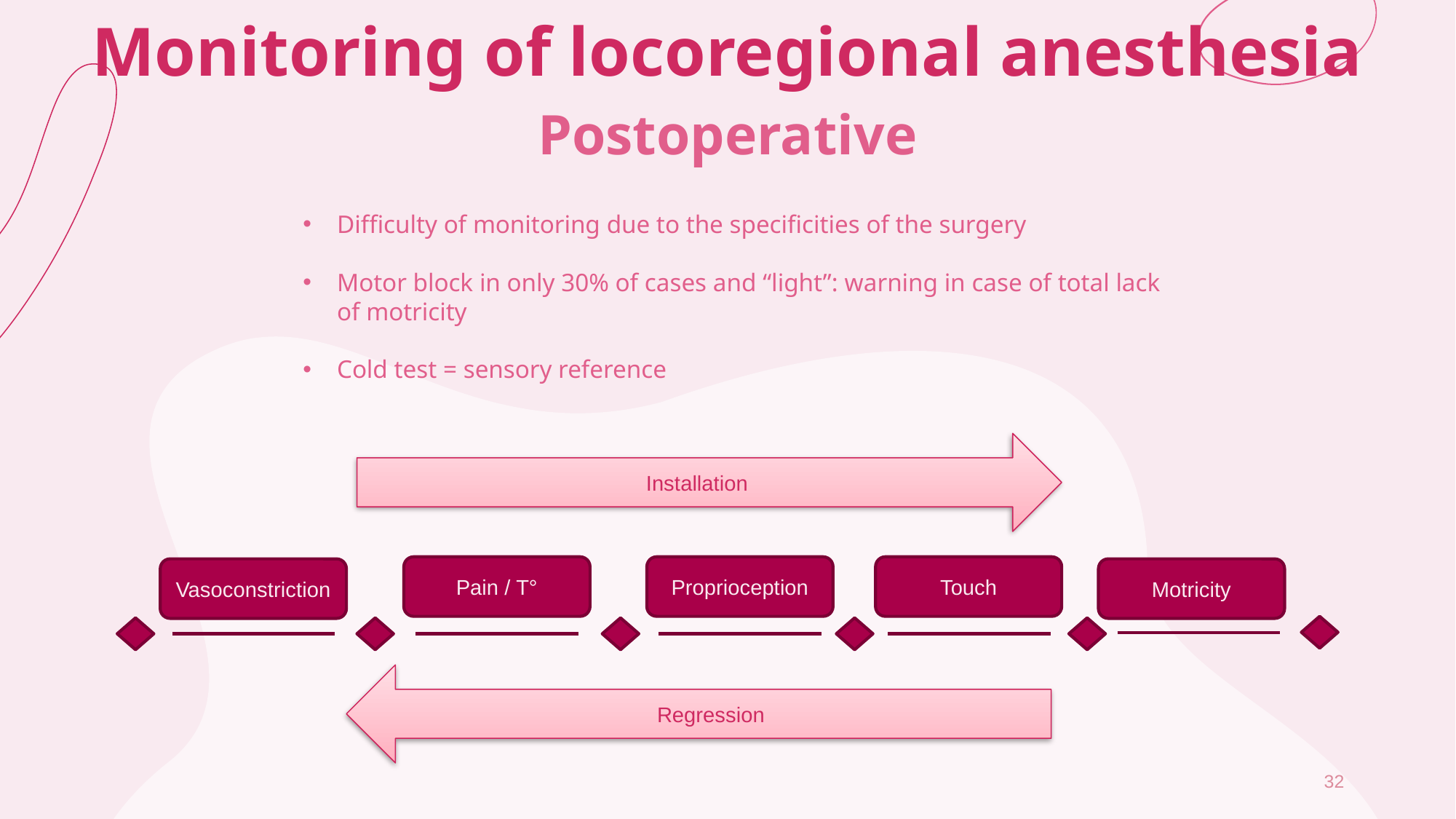

Monitoring of locoregional anesthesia
Postoperative
Difficulty of monitoring due to the specificities of the surgery
Motor block in only 30% of cases and “light”: warning in case of total lack of motricity
Cold test = sensory reference
Installation
Touch
Proprioception
Pain / T°
Vasoconstriction
Motricity
Regression
32

## Slide 33
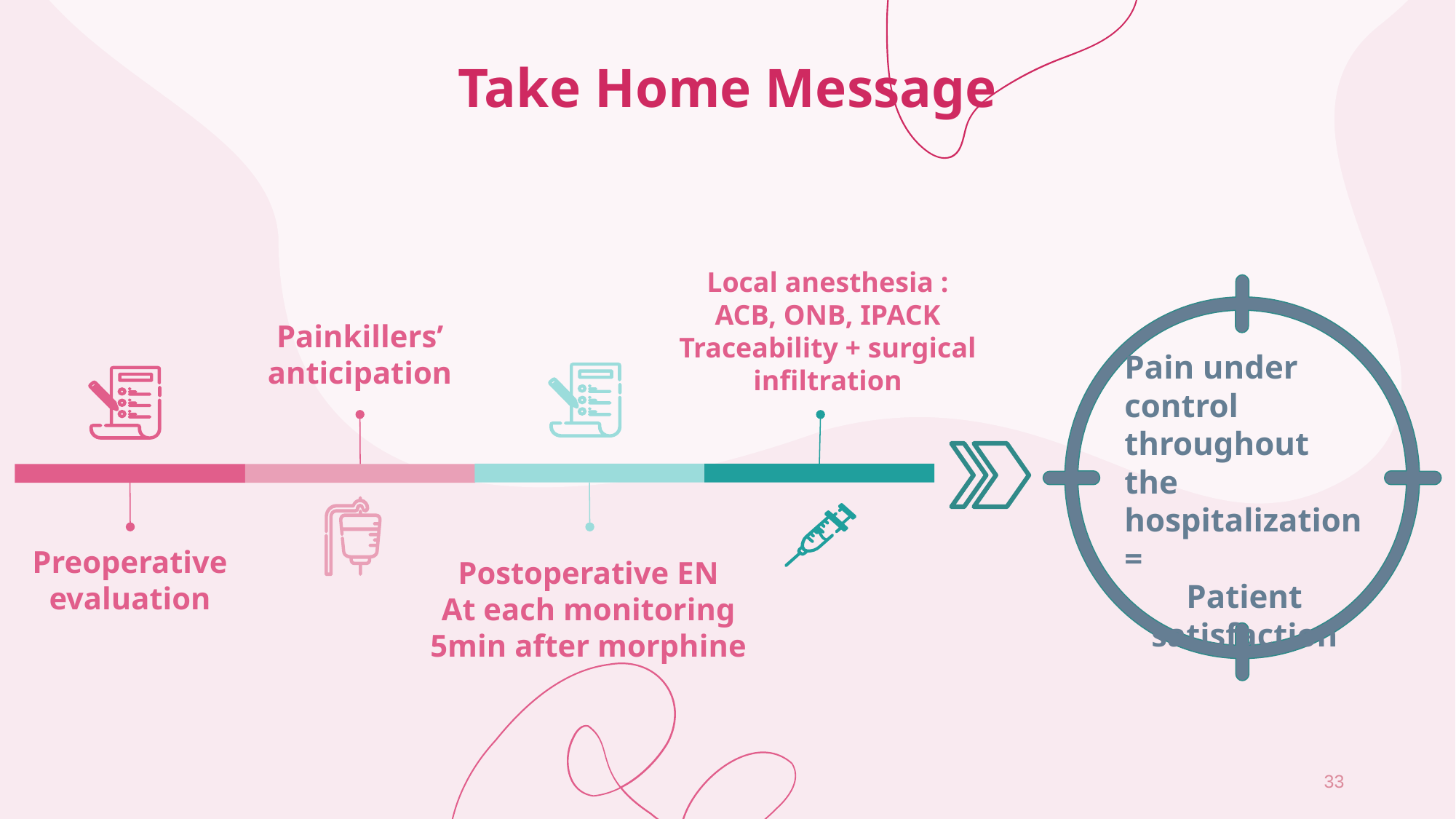

# Take Home Message
Local anesthesia :
ACB, ONB, IPACK
Traceability + surgical infiltration
Painkillers’ anticipation
Pain under control throughout the hospitalization
=
Patient satisfaction
Preoperative evaluation
Postoperative EN
At each monitoring
5min after morphine
33

## Slide 34
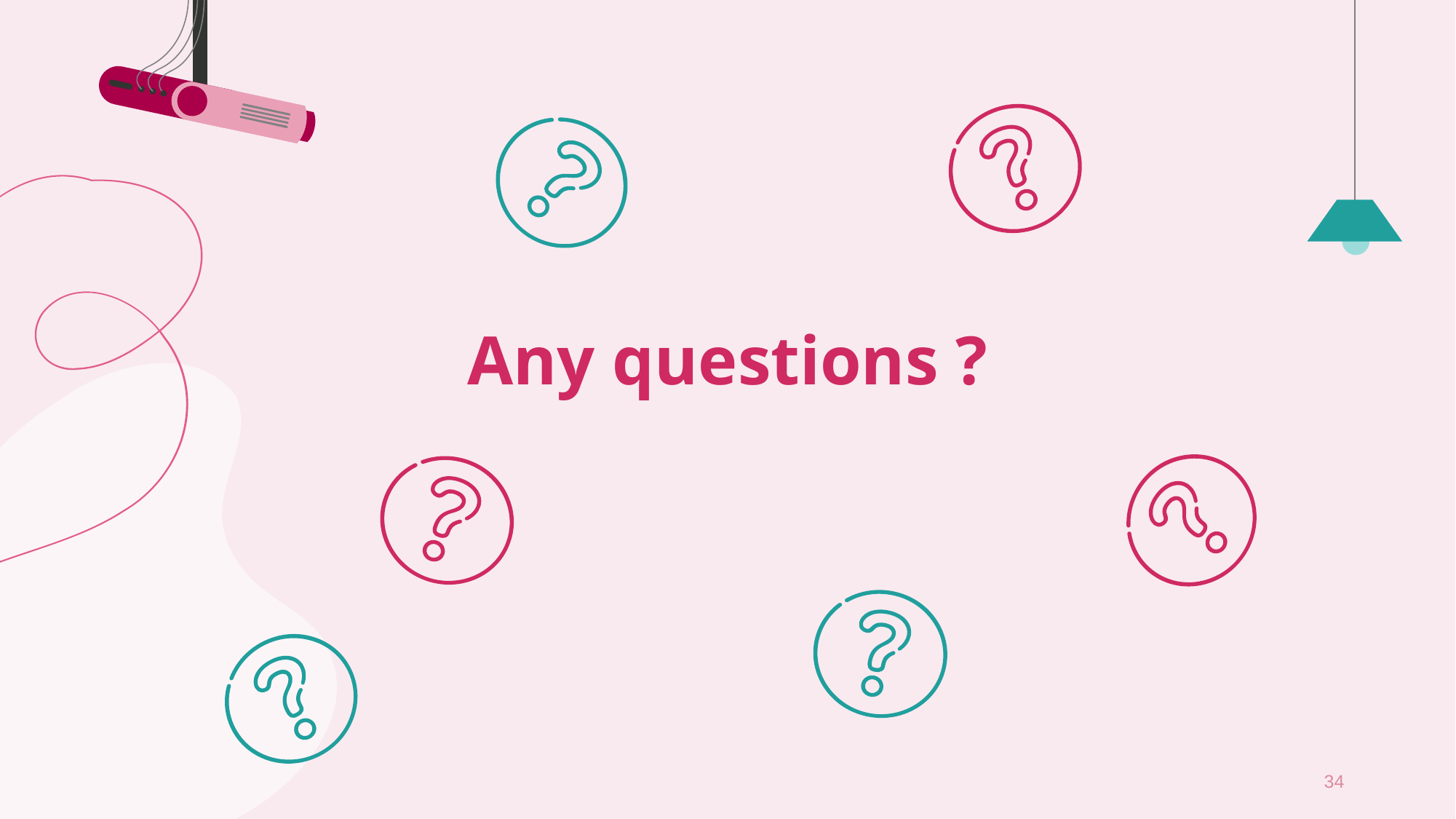

# Any questions ?
34

## Slide 35
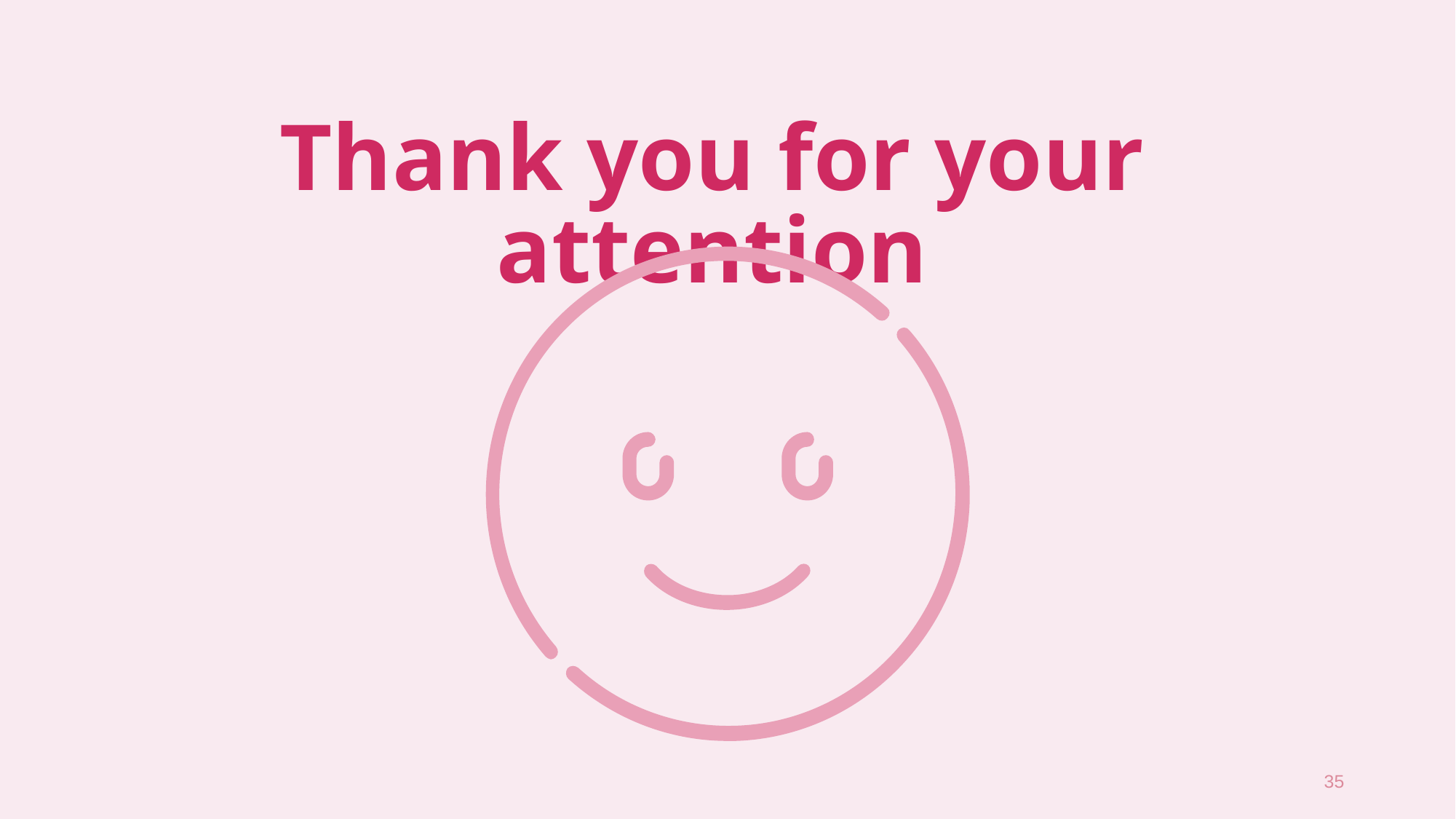

# Thank you for your attention
35

## Slide 36
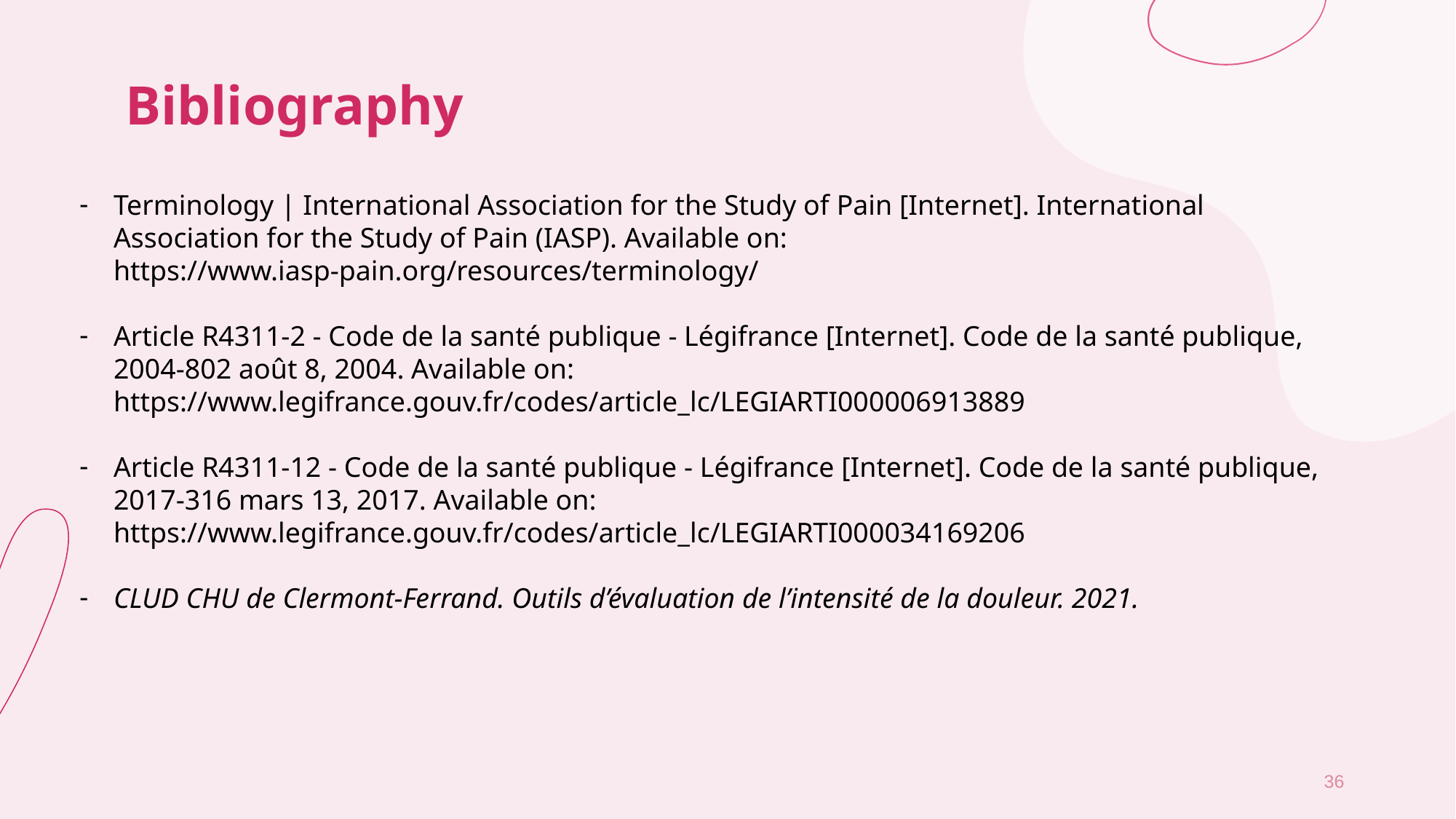

# Bibliography
Terminology | International Association for the Study of Pain [Internet]. International Association for the Study of Pain (IASP). Available on: https://www.iasp-pain.org/resources/terminology/
Article R4311-2 - Code de la santé publique - Légifrance [Internet]. Code de la santé publique, 2004‑802 août 8, 2004. Available on: https://www.legifrance.gouv.fr/codes/article_lc/LEGIARTI000006913889
Article R4311-12 - Code de la santé publique - Légifrance [Internet]. Code de la santé publique, 2017‑316 mars 13, 2017. Available on: https://www.legifrance.gouv.fr/codes/article_lc/LEGIARTI000034169206
CLUD CHU de Clermont-Ferrand. Outils d’évaluation de l’intensité de la douleur. 2021.
36
